# Improving the sustainability of the ruthenium-catalysed *N*-directed C–H arylation of arenes with aryl halides†

**DOI:** 10.1039/d2gc03860a

**Published:** 2023-03-07

**Authors:** Michael T. Findlay, Ashley S. Hogg, James J. Douglas, Igor Larrosa

**Affiliations:** a Department of Chemistry, School of Natural Sciences, University of Manchester Oxford Road Manchester M13 9PL UK igor.larrosa@manchester.ac.uk; b Early Chemical Development, Pharmaceutical Sciences, R&D, AstraZeneca Macclesfield UK

## Abstract

Direct C–H functionalisation methodologies represent an opportunity to improve the overall ‘green’ credentials of organic coupling reactions, improving atom economy and reducing overall step count. Despite this, these reactions frequently run under reaction conditions that leave room for improved sustainability. Herein, we describe a recent advance in our ruthenium-catalysed C–H arylation methodology that aims to address some of the environmental impacts associated with this procedure, including solvent choice, reaction temperature, reaction time, and loading of the ruthenium catalyst. We believe that our findings demonstrate a reaction with improved environmental credentials and showcase it on a multi-gram scale within an industrial setting.

## Abstract 2

A ruthenium-catalysed C–H arylation procedure that utilises a range of green solvents in place of the undesirable solvent NMP is presented. Examples of fast reaction time, low catalyst loading and large-scale reactivity are also shown.

## Graphical abstract

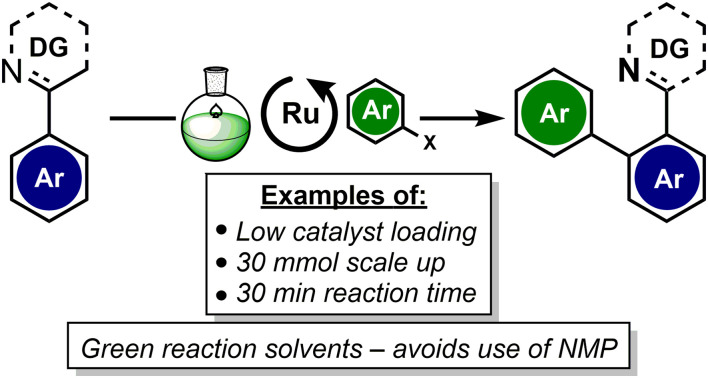

## Introduction

Given the ubiquitous nature of C–C bonds in organic molecules, the development of new methods for their construction is fundamental to the field of organic synthesis. Typical methods for the construction of these bonds involve the use of transition-metal-catalysed (typically palladium) cross-coupling reactions.^1,2^ While these reactions offer facile access to a diverse array of molecular frameworks, the requirement for pre-functionalisation of starting materials reduces the attractiveness of this route. Not only is excess metal–halide waste generated during the catalytic process, but also the extra steps required to install functional handles generate further excess waste, resulting in decreased atom economy of the overall process and unnecessary use of time and energy.^3,4^

**Fig. 1:**
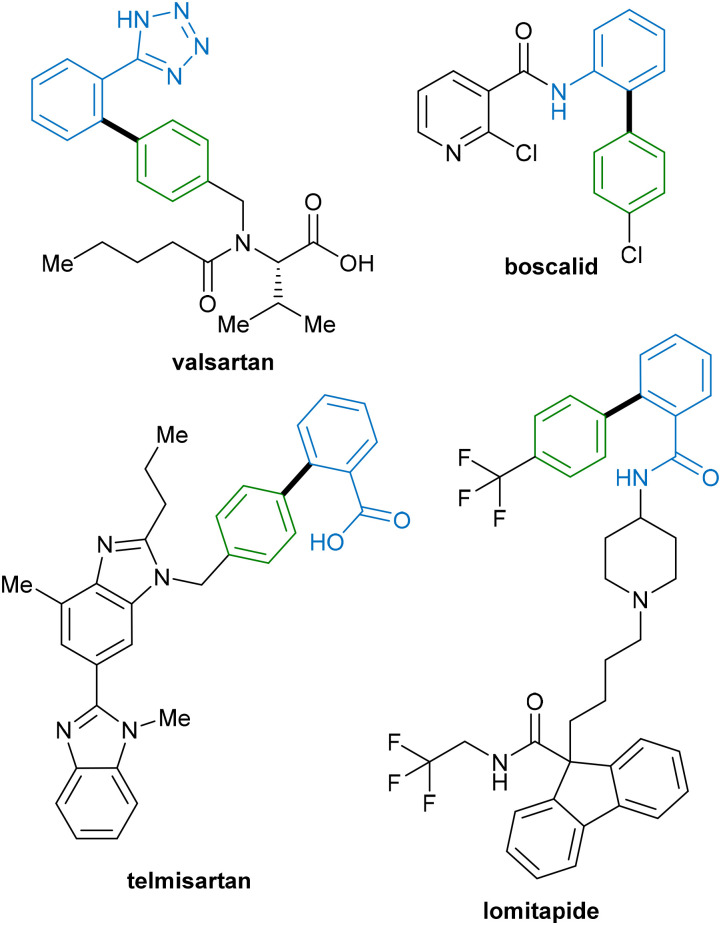
Drugs containing biphenyl groups currently synthesised by cross coupling.

An alternative method that can provide a more economical approach to C–C bond formation is the direct functionalisation of C–H bonds, which has emerged as a popular method in the last few decades.^5–7^ Indeed, the development of equally robust direct C–H functionalization methodologies, which are capable of forming new functional groups without requiring prior functionalisation of one or both starting materials, represents an attractive target for organic methodologies. In addition, in the future these methods could provide a more streamlined approach to the synthesis of drugs and their analogues that are currently synthesised by cross-coupling (Fig. 1). In contrast to cross-coupling approaches, C–H functionalisation allows the direct construction of functionalised molecules from abundant and relatively inert C–H bonds, reducing the overall step count and reaction waste, and improving overall reaction efficiency.^8^

However, sustainability is still a challenge in the C–H functionalization area. Firstly, these procedures often employ second and third row transition metals that can be both hazardous to the environment and harmful to health. These metals also have a finite supply, and as such, their use is unsustainable in the long term.^9^ Due to these issues, efforts should be made to improve the activity of these catalysts, allowing the development of protocols that utilise low catalyst loadings. In the long term, we should seek to replace these metals with more abundant and less toxic alternatives, and recycle catalysts where possible. Secondly, the high C–H bond dissociation enthalpy (≈110 kcal mol^−1^ for C(aryl)–H bonds) frequently results in harsh reaction conditions for these transformations. This often limits the functional group tolerance observed, as many of the sensitive groups present in complex molecules are unstable under these conditions. High temperatures also lead to increased energy consumption that can become prohibitively expensive and environmentally impactful at the process scale. Finally, C–H activation reactions commonly require the use of solvents that are classed as hazardous to either people or the environment, by being volatile, toxic, flammable, and/or explosive.^10,11^ Solvents also often account for a large portion of the cost, waste generated, and CO_2_ footprint of a reaction, as they are commonly used in a large excess relative to the substrate.^12–14^ As such, synthetic organic chemistry would benefit greatly from the development of procedures that work efficiently in non-hazardous, renewable, and environmentally friendly solvents.

Unfortunately, the selection of an optimal ‘green’ solvent for a chemical reaction is not trivial, as no standardised rating exists that holds true for all aspects of interest. Many pharmaceutical companies have developed solvent selection guides, which enable users to select solvents based on their reaction considerations.^15–20^ Solvents are classified based on multiple data points, which can be contradictory, and include, but are not limited to, reaction efficiency, safety, environment, quality, practicality, availability, and cost. Therefore, an ideal chemical reaction would perform consistently in multiple different solvents, allowing the end user to select a reaction solvent consistent with the requirements of their process.

**Scheme 1:**
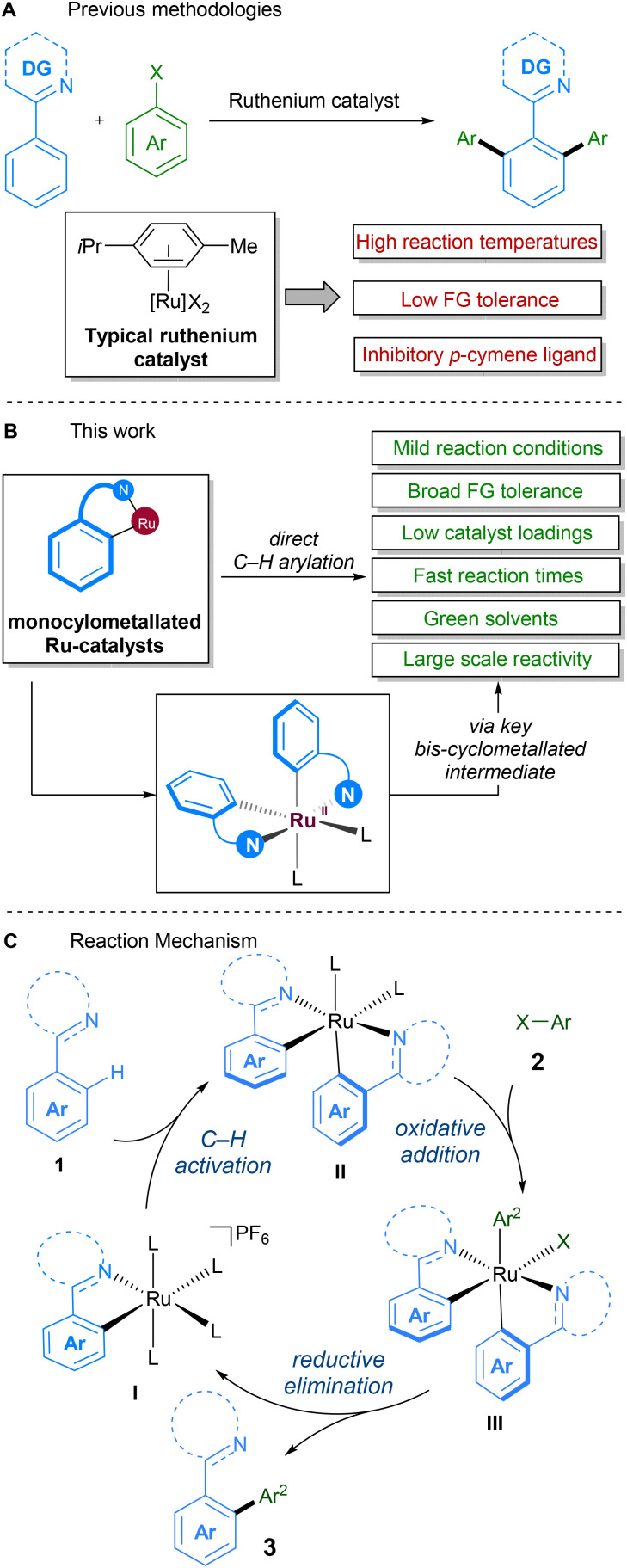
(A). Previous ruthenium-catalysed direct arylations using *para*-cymene ligated catalyst; (B). This work: improved sustainability of ruthenium-catalysed direct-arylations using latest class of mono-cyclometallated complexes. (C). Proposed reaction mechanism. *L* = one or more of acetonitrile or solvent as neutral ligands.

Due to the historically lower cost of ruthenium *vs.* other commonly used precious metals and the complementary reactivity it displays, ruthenium catalysts have become popular in the development of C–H activation procedures.^21^ Early work on direct alkylations with ruthenium by Murai^22^ was followed up in 2001, in the seminal report by Oi and Inoue on the ruthenium-catalysed C–H arylation and allylation of directing group-containing arenes.^23^ Further work on ruthenium-catalysed C–H arylation by other groups since then has demonstrated the feasibility of replacing classical solvents with green variants (Scheme 1A).^24^ Particular attention has been focussed on reactions in 2-methyltetrahydrofuran,^25–28^ PEG-400,^29,30^ diethyl carbonate,^31^ deep-eutectic solvents,^32^ and water.^33,34^

In 2018, our group reported a direct C–H arylation procedure that was able to proceed at ambient temperature.^35–37^ These studies revealed that the *p*-cymene ligand commonly found on ruthenium prevents formation of the necessary bis-cyclometallated Ru(ii) species. As such, the use of arene-ligated-free mono-cyclometallated catalyst, RuBnN, led to a significant improvement in catalytic activity, allowing the use of mild reaction conditions, and compatibility with a wide range of coupling partners. This was the first report of ruthenium catalysed C–H arylation performed at low temperatures, and has since been reported under photochemical conditions by the groups of Greaney,^38^ Ackermann^39^ and Zhang.^40^

Following on from this, we set about further developing a C–H arylation procedure that demonstrated improved sustainability when compared to previous methods (Scheme 1B). To this end, we aimed to engineer a system capable of both operating at ambient temperatures and with a variety of different ‘green’ solvents. We also aimed to improve other aspects of the procedure – catalyst loading, reaction time, and reaction scale – to further enhance its sustainability. The mechanism for our procedure was proposed to proceed *via* a different pathway to that proposed for over two decades for directed C–H arylation (Scheme 1C). Instead of the previously proposed oxidative addition directly to a monocyclometallated intermediate, we found that a second C–H activation event to I leading to the formation of bis-cyclometallated intermediate II was required in order to facilitate the oxidative addition step. Subsequent reductive elimination from ruthenium(iv) intermediate III then led to the arylated product and reformed the mono-cyclometallated ruthenium complex I, that can undergo further catalytic turnover.

## Results and discussion

We started our screen by employing the initial conditions from our previously reported C–H arylation procedure, using 2-phenylpyridine 1a, 3,5-bromo-*meta*-xylene 2a as substrates, and *N*-methylpyrrolidone (NMP) as solvent (Table 1, entry 1). NMP is known to have adverse effects on human health, have issues regarding negative environmental impact from its waste, and since 2011, has been listed on the ECHA's list of ‘substances of very high concern’ (SVHC) due to being toxic for reproduction – limiting its industrial applications.^41^ For these reasons, in order to avoid its use, we switched to dimethyl carbonate (DMC), previously reported by Dixneuf for ruthenium catalysed C–H arylation,^31^ as an alternative green solvent. Comparison of these two solvents at a reduced reaction time of 100 min demonstrated a significant deceleration in rate when performed in DMC (entry 2), generating only a combined 5% of products 3aa and 4aa, *vs.* 26% for when using NMP. A small screen of various additives was then performed in order to accelerate the rate of the reaction in DMC. Replacing KOAc with other alkyl and aryl carboxylates, including levulinic acid, which has shown to be beneficial in a previous procedure,^42^ appeared to have little influence on the reaction rate, performing equally badly (entries 3–5). Addition of pivalamide^31^ as an additive (entry 6) that has shown beneficial effects in a previous report by Dixneuf, was also found to have little effect. Gratifyingly, we found that higher yields of products 3aa and 4aa were achieved when using tetrabutylammonium acetate (TBAOAc) as additive (entry 7), which has been previously shown to facilitate these types of reaction by acting as both base and solvent when using high reaction temperatures.^43^ Interestingly, switching to the structurally related additives tetraethylammonium acetate (TEAOAc, entry 8) and tetramethylammonium acetate (TMAOAC, entry 9) also resulted in an increase in reactivity compared to KOAc, albeit inferior to TBAOAc. Under these conditions full conversion of 1a into 4aa could be achieved in less than 8 h (entry 10) without requiring an excess of either coupling partner.

**Table 1:** Additive optimisation of reaction using green solvent

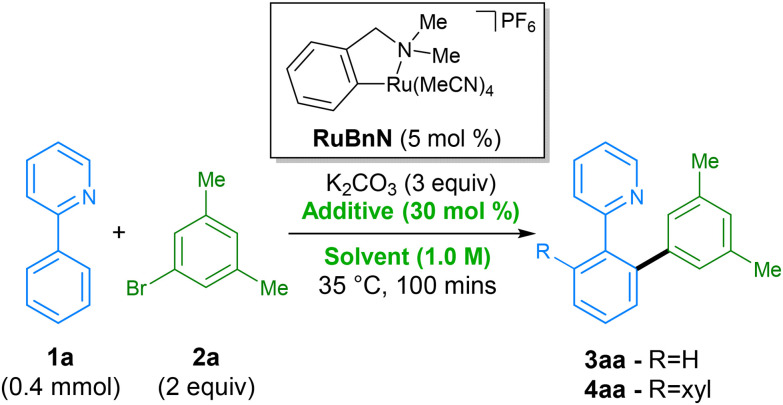				
Entry	Solvent	Additive	3aa (%)	4aa (%)
1	NMP	KOAc	7	19
2	DMC	KOAc	3	2
3	DMC	KCO_2_Ad	2	2
4	DMC	KO_2_CMes	3	1
5	DMC	Levulinic acid	4	2
6^a^	DMC	KOAc	2	2
7	DMC	TBAOAc	4	21
8	DMC	TEAOAc	5	17
9	DMC	TMAOAc	6	16
10^b^	DMC	TBAOAc	0	97

a Pivalamide (30 mol%) added as additive.

b Reaction run for 8 h.

**Scheme 2:**
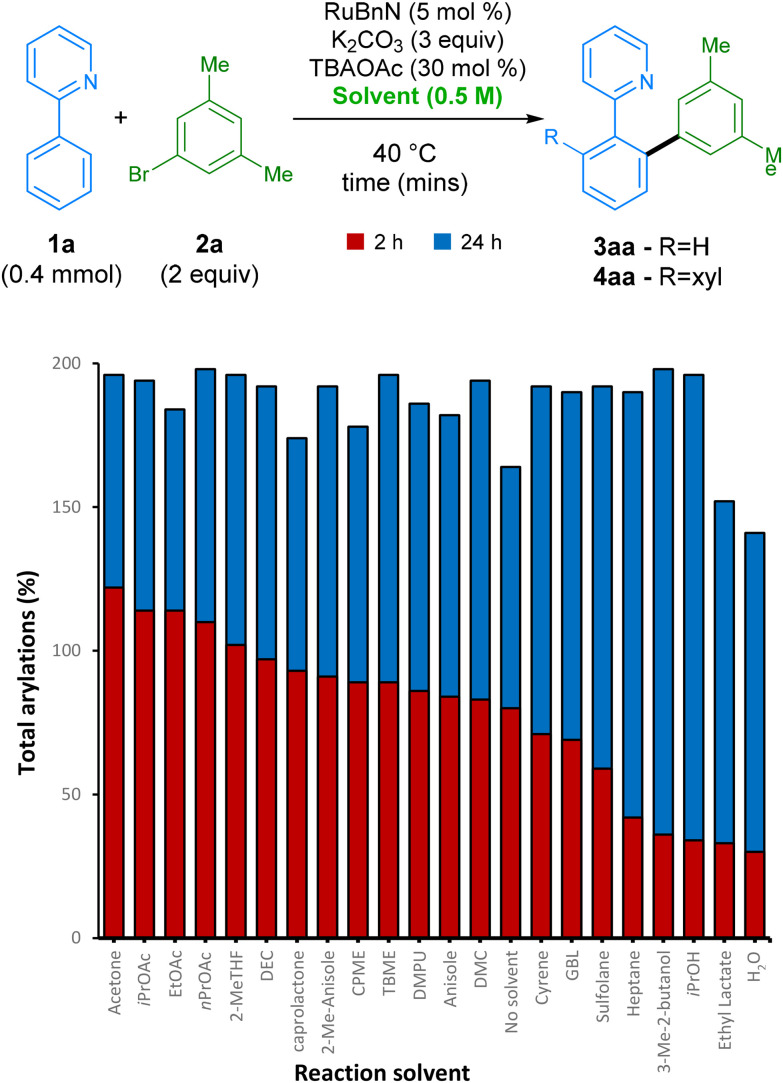
Screen of green solvents at different reaction times. Yields were measured by GC-FID analysis of the crude reaction mixture using hexadecane as the internal standard. Solvent acronyms can be found in endnotes.^46^

After finding our optimal choice of additive, we investigated the ability of this reaction to proceed in a wide range of green solvents (Scheme 2). Under these conditions, over 90% yield was achieved in 16 different reaction solvents at 35 °C. Carbonate derived solvents dimethyl- (DMC) and diethyl- carbonate (DEC) worked well, along with structurally similar acetate esters. Lactone solvents ε-caprolactone and γ-butyrolactone (GBL) also functioned well, delivering product 4aa in high yields. Alcohol solvents iPrOH and 3-methyl-butan-1-ol were also proficient at facilitating the reaction, along with industrially preferred ethereal solvents TBME and CPME. A range of other industrially preferred solvents, such as sulfolane, DMPU, 2-methylanisole, and 2-Me-THF, also delivered arylation product 4aa in good yields, the latter being favourable due to its low carbon footprint.^44^

As perhaps the ideal ‘green’ solvent, water is also capable of acting as a medium for this arylation procedure, despite giving a slightly lower yield of 70%. Interestingly, the reaction even achieves a high conversion to products when run under the same conditions but in the absence of any formal solvent. However, this is not always favourable in the interest of safety, and its efficiency in this case is likely to be highly dependent on the nature of the substrates employed.^45^

**Scheme 3:**
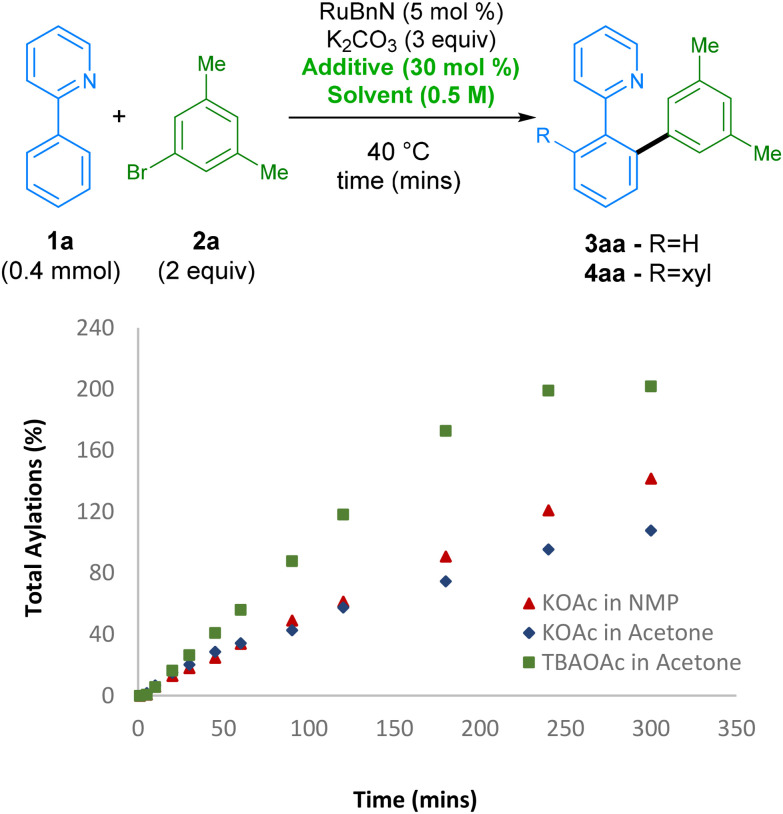
Comparison of reaction rates varying solvent and additive. Reactions monitored by GC-FID analysis of the crude reaction mixture using hexadecane as the internal standard.

Next,^46^ we further investigated the effect of the TBAOAc additive on the reaction rate by monitoring the reaction profile under various reaction conditions (Scheme 3). Initially, using KOAc as additive and switching from NMP to acetone, which had the highest conversion after 2 h, led to a slower reaction rate. However, switching the additive from KOAc to TBAOAc had a noticeable effect on the reaction rate, with the reaction reaching ccomplete conversion to the bis-arylated product 4aa in under 4 h, revealing a set of conditions that are not only greener than the original set, but also considerably more reactive. The origin of the effect possibly arises from the change in solubility of KOAc when switching solvent. TBAOAc, commonly employed as a phase transfer catalyst, is likely to be present in a higher effective concentration when compared with KOAc.

**Scheme 4:**
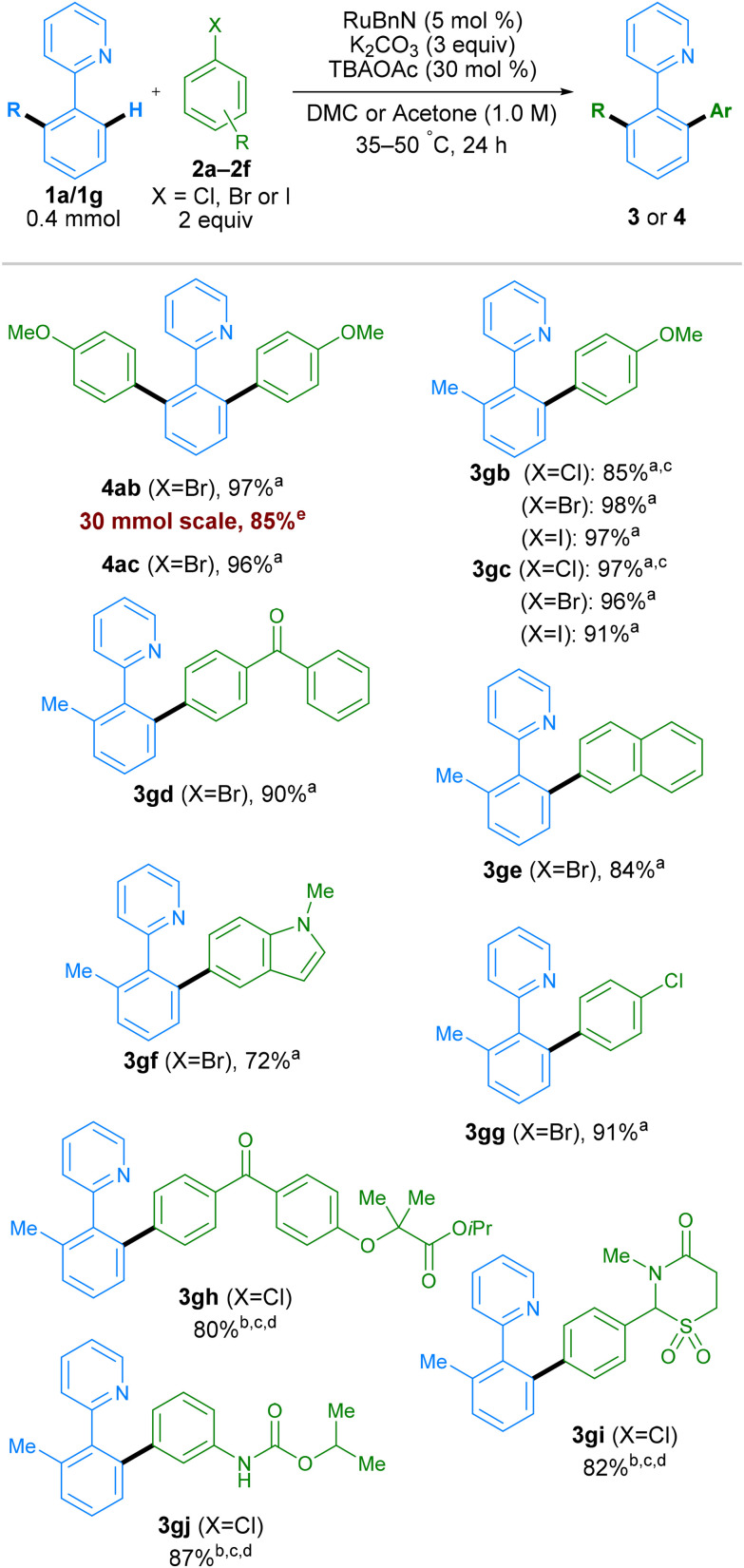
Scope of aryl halide coupling partner. All yields are isolated yields. ^a^DMC as solvent. ^b^Acetone as solvent. ^c^50 °C. ^d^RuBnN (10 mol%). ^e^30 mmol scale reaction performed in the laboratory of AstraZeneca at 0.5 M for 4.5 h.

To determine if the new conditions were compatible with a broad range of substrates, we next investigated the scope of the reaction with a variety of coupling partners (Scheme 4). In addition to bromo-*meta*-xylene, other bromoarenes, bearing either electron donating (–OMe) or electron withdrawing (–CF_3_) substituents in the *para*-position, delivered products 4ab and 4ac in high yields. Mono-arylation was possible when using 2-tolylpyridine 1g as a substrate, working with both electron-withdrawing and electron-donating groups on the electrophilic coupling partner, using aryl chlorides, bromides or iodides, in all cases with excellent yields. Examples aryl bromides containing ketones, naphthalene, *N*-Me-protected indole, and aryl chloride groups also worked well, generating products 3gd, 3ge, 3gf, and 3gg respectively in good yields. The reaction conditions were assessed at multi-gram scale within the industrial laboratory of AstraZeneca employing apparatus representative of large-scale manufacture. A 5-gram reaction (30 mmol) using DMC (0.5 M) as solvent, proceeded to give 4ab in 85% yield, demonstrating the reproducibility and scalability of this method. More complex molecules containing aryl halide moieties were also shown to react smoothly, allowing coupling between 1g and fenofibrate, chlormezanone and chlorpropham, to generate coupled products 3gh, 3gi and 3gj.

**Scheme 5:**
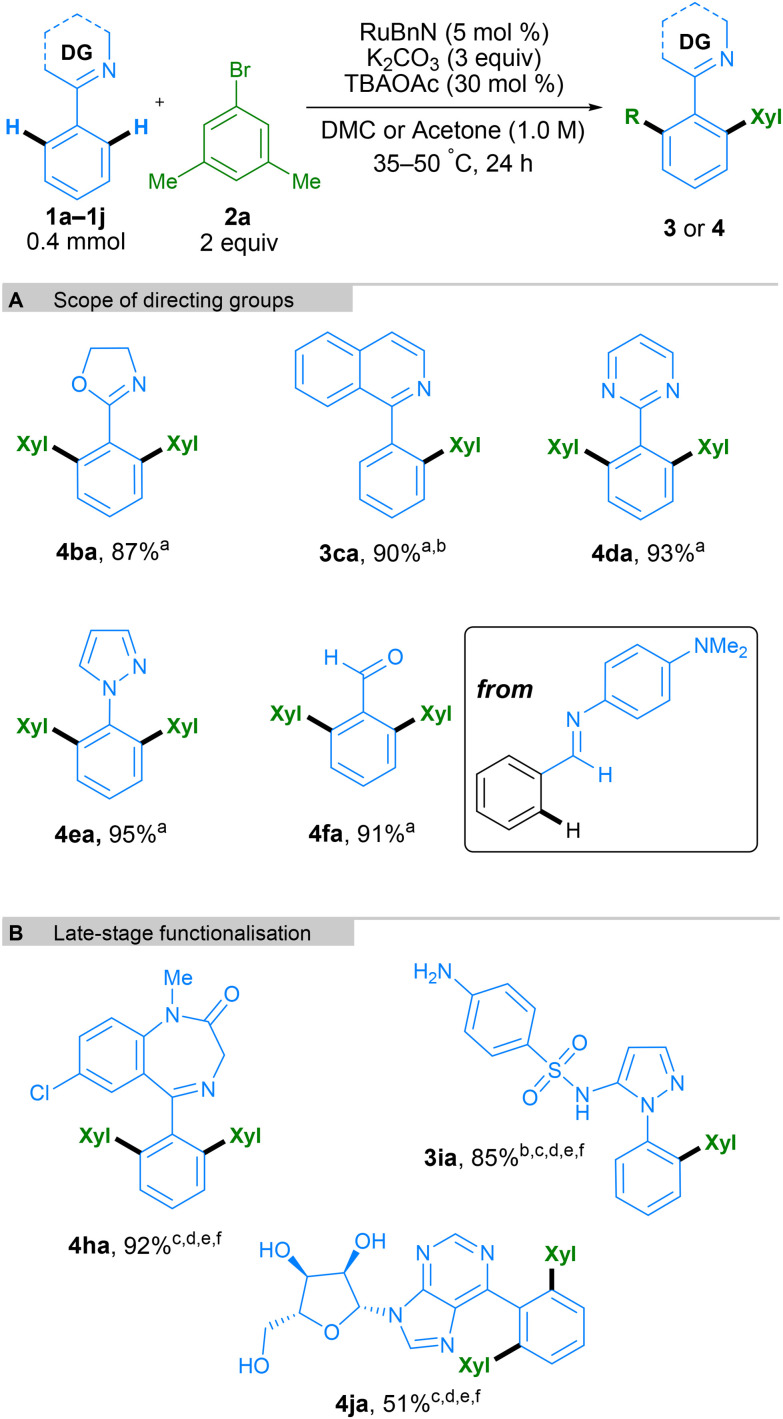
Scope of *N*-directing group containing arenes and late-stage functionalization. All yields are isolated yields. Xyl = 3,5-dimethylphenyl. ^a^DMC as solvent. ^b^2 (1 equiv.). ^c^Acetone as solvent. ^d^50 °C. ^e^RuBnN (10 mol%). ^f^48 h.

Next, we tested a range of nitrogen-based directing groups to demonstrate the versatility of this methodology (Scheme 5A). In addition to pyridine as directing group, oxazoline (4ba), isoquinoline (3ca), pyrimidine (4da), and pyrazole (4ea) groups were all able to direct the C–H functionalisation successfully. Functionalisation *ortho* to an aldehyde on the phenyl ring was also possible through the use of an *N*-aryl imine directing group, generating the final product after acidic workup (Scheme 5A, 4fa). To further exhibit the utility of this method, it was applied to the late-stage functionalisation of some complex molecules (Scheme 5B). Diazepam, an anxiolytic medicine from the benzodiazepine family, was successfully bis-arylated using 10 mol% Ru catalyst and acetone as the solvent. Similarly, Sulfaphenazole, a sulfonamide antibacterial drug containing a pyrazole directing group, led to mono-arylation to form 3ia in a high yield. Finally, 6-phenylpurineriboside, containing an unprotected sugar moiety and a purine directing group, also led to efficient bis-arylation to form product 4ja. It is interesting to note that whilst bis-arylation is generally observed, 3ca and 3ia lead to exclusively mono-arylated products. In these cases, the second C–H activation needed to form the bis-arylated product is slower, likely due to their structure, and hence the mono-arylated product is obtained selectively.

**Scheme 6:**
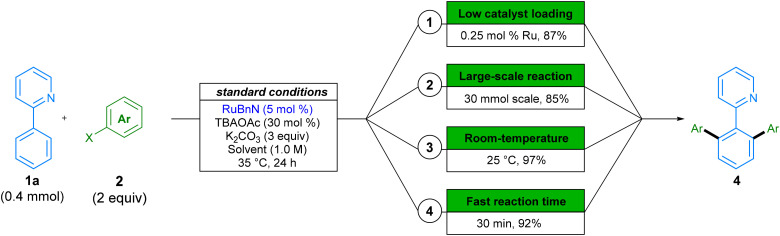
Examples of targeted tuning of reaction conditions. Variations from standard conditions shown: (1) 2a, RuBnN (0.25 mol%), 70 °C, DMC; (2) 2b, 30 mmol scale, 4.5 h, 50 °C, DMC; (3) 2a, 25 °C, DMC; (4) 2a, 70 °C, DMC, 30 min. Yields are of isolated compounds.

In addition to the wide variety of solvents and substrates that the reaction tolerates, we investigated further modifications to the reaction conditions that would improve the environmental impact of this procedure (Scheme 6). Heavy metal transition metal catalysts are a finite resource, and as such, it is necessary to use them in a sustainable fashion. The relative scarcity and global market trends can lead to fluctuations in price and availability, and their toxicity requires a reduction in the waste generated. Accordingly, we aimed to provide an example in which we reduce the catalyst loading of our procedure. This was achieved by increasing the reaction temperature from 35 °C to 70 °C in the reaction forming product 4aa, and we were able to obtain 87% in 24 h, by using only 0.25 mol% of our ruthenium catalyst (Scheme 6.1). To the best of our knowledge, this example is the lowest catalyst loading of any ruthenium-catalysed C–H arylation with aryl halide coupling partners reported to-date, corresponding to a TON of 348.^47^ Next, we provide an example of reaction scale-up by running the reaction on a 30 mmol, in a set-up representative of large-scale manufacture. A yield of 85% demonstrates the reproducibility and scalability of this method (Scheme 6.2). Subsequently, as energy costs can contribute significantly to the cost of chemical processes,^13^ we showed that dropping the temperature to 25 °C still affords high conversion to product 4aa within 24 hours (Scheme 6.3). Similarly, for early-stage target optimisation processes, time can be an influential factor in reaction choice, and longer reactions can present a bottleneck in linear syntheses.^48^ By simply increasing the reaction temperature to 70 °C, we can obtain 92% of product 4aa in just 30 min of reaction time (Scheme 6.4).

## Conclusions

In conclusion, we have developed a C–H functionalisation procedure that allows the *ortho*-arylation of arenes containing *N*-directing groups, using conditions that possess a marked improvement in sustainability as well as efficiency. This method avoids the use of NMP, a common solvent in Ru-catalyzed arylation. Instead, this procedure is compatible with a wide range of solvents, with enhanced green credentials, allowing the eventual user to employ a solvent which is consistent with the specific goals of their process. Conditions can be easily tuned to achieve lower catalyst loadings, performance at room temperature, or reaction times as fast as 30 min, depending on the required usage. Furthermore, the reaction was conducted on multi-gram scale within an industrial setting, operating in comparable yield.

## Conflicts of interest

There are no conflicts to declare.

## Supplementary Material

GC-025-D2GC03860A-s001
