# Supplementary material for: Improving the sustainability of the ruthenium-catalysed N-directed C–H arylation of arenes with aryl halides
Source: Green Chem. 2023 Mar 7;25(6):2394–400. doi: 10.1039/d2gc03860a (PMC10026369; doi:10.1039/d2gc03860a)
Supplement: GC-025-D2GC03860A-s001 [file GC-025-D2GC03860A-s001.pdf]

Electronic Supplementary Material (ESI) for Green Chemistry.
This journal is © The Royal Society of Chemistry 2023

Supporting Information

Improving the sustainability of the ruthenium-catalysed *N*-directed C–H arylation of arenes with aryl halides

Michael T. Findlay,[a] Ashley S. Hogg,[a] James J. Douglas[b] and Igor Larrosa[a]

[a]Department of Chemistry, School of Natural Sciences, University of Manchester, Oxford Road, Manchester, M13 9PL, UK

[b]Early Chemical Development, Pharmaceutical Sciences, R&D, AstraZeneca, Macclesfield, UK

1. General information........2
2. Preparation of Ruthenium Complexes........3
3. Starting Material Preparation........4
4. General Procedures........7
5. Product Characterisation Data........9
6. Reaction Optimization........23
7. Solvent Screen........26
8. Reaction Comparisons........29
9. Improved Reaction conditions........33
10. Copies of NMR spectra........39
11. References........62

1. General information

All the reactions were set up in an argon filled glovebox with oven-dried crimp cap microwave vials (10 mL). The reactions were then capped (using PK100 20MM BUTYL SEPTA) and taken outside the glovebox to run. All starting materials were purchased from Acros (Fisher), Aldrich (Merck), Alfa Aesar (Fisher) and Fluorochem and used without further purification unless stated otherwise. Column chromatography was carried out on silica gel (particle size 40-63 µm) using flash techniques. High resolution mass spectra were performed by the School of Chemistry Mass Spectrometry Service (University of Manchester) employing a Thermo Finnigan MAT95XP spectrometer. IR spectra were recorded using a Thermo Scientific Nicolet iS5 FTIR machine, relevant bands are quoted in cm^{-1}. ^{1}H NMR, ^{19}F NMR and ^{13}C NMR spectra were all recorded on Bruker instruments in the School of Chemistry NMR service (University of Manchester). ^{1}H NMR are referenced to the residual solvent peak at 7.26 ppm ($CDCl_3$), 1.94 ppm (CD_3CN), or 2.05 ppm ($(CD_3)_2CO$). ppm values are quoted to 2 decimal places, with coupling constants (J) to the nearest 0.1 Hz. ^{13}C NMR spectra were quoted in ppm to 1 decimal place with coupling constants (J) to the nearest 0.1 Hz. The spectra were referenced to the residual solvent peak at 77.16 ppm ($CDCl_3$), 1.32 ppm (CD_3CN), or 39.52 ppm ($(CD_3)_2CO$). ^{19}F NMR spectra are quoted in ppm to 1 decimal place with coupling constants (J) to the nearest 0.1 Hz.

2. Preparation of Ruthenium Complexes

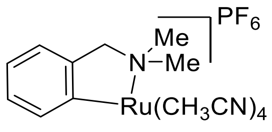

RuBnN

Figure S1. Structure of RuBnN catalyst.

RuBnN: An oven dried 100 mL Ace pressure tube equipped with a stirring bar was transferred to a glove box, then $[RuCl_2(benzene)]_2$ (440 mg, 0.88 mmol, 0.55 equiv), NaOH (96 mg, 2.4 mmol, 1.5 equiv), KPF_6 (589 mg, 3.2 mmol, 2 equiv), N,N-dimethylbenzylamine (216 mg, 1.6 mmol, 1 equiv) and MeCN (10 mL, 0.16 M) were added. The tube was sealed, transferred out of the box and placed in an oil bath at 45 °C and the reaction was stirred for 3 h. Upon completion, the reaction crude was loaded in an aluminium oxide (Al_2O_3, neutral) column conditioned with CH_2Cl_2, and quickly eluted with MeCN under N_2 collecting the yellow/orange band. The solution was concentrated under reduced pressure and then quickly precipitated with Et_2O affording a yellow solid/orange solid, which was promptly transferred to a glove box as it decomposes turning green/blue if exposed to air. This solid and MeCN (20 mL) were added to an oven dried 100 mL Ace pressure tube equipped with a stirring bar and the reaction was stirred for 24 h at 100 °C. After this time, the reaction mixture was filtered through a short plug of aluminium oxide, eluted with MeCN, concentrated under vacuum and precipitated with Et_2O/pentane (1:1) affording **RuBnN** as an off-white solid (653 mg, 75%). **RuBnN** must be kept in a glove box as it quickly decomposes turning blue/black if exposed to air. The complexes are subjected to quantitative ^1H NMR after their synthesis. They are generally in the region of 99-100% pure by this measure. If they are of lower purity, then the complex should be dissolved in MeCN inside the glovebox and filtered through a small plug of alumina. Then it should be concentrated under vacuum and crashed out with Et_2O/Pentane (1:1).

^{1}H NMR (400 MHz, CD_3CN) δ 7.56 (d, J = 7.6 Hz, 1H), 6.88-6.95 (m, 2H), 6.76 (t, J = 7.4 Hz, 1H), 3.64 (s, 2H), 2.46 (s, 6H), 2.42 (s, 3H), 2.23 (s, 6H), 1.97 (s, 3H); **^{13}C NMR** (100 MHz, CD_3CN) δ 175.2, 149.6, 138.6, 125.3, 123.3, 123.1, 121.5, 121.2, 73.6, 53.7, 4.4, 4.2; **^{19}F NMR** (376 MHz, CD_3CN) δ –72.9 (d, J = 705.4 Hz) ppm; IR ν_{max} (neat/cm^{-1}): 3048, 2977, 2926, 2897, 2855, 2265, 1575, 1472, 1442, 831, 748; **HRMS** calcd for $C_{15}H_{21}N_4Ru^+$ $[M–MeCN– PF_6]^+$: 359.0804. Mass found: 359.0798.

3. Starting Material Preparation

3.1 General notes

Additives and bases were all bought from commercial suppliers, with solid reagents ground up using a pestle and mortar and dried in a vacuum oven for a minimum of 24 h prior to being transferred into the glove box for use. All solvents were bought from commercial suppliers and degassed in J Young sample flasks using the freeze-pump-thaw method, with a minimum of 3 cycles, prior to being transferred into the glove box for use.

3.2 *N*-directing group coupling partners

Substrates **1a, 1c, and 1e** were purchased from the commercial supplier Fluorochem. Substrate **1b** was prepared from the corresponding nitrile and aminoalcohol in a literature-reported procedure.[1] Substrates **1d**[2] and **1g**[3] were prepared by Suzuki-Miyaura coupling between phenylboronic acid and the corresponding aryl halide, in literature-reported procedures. Imine substrate **1f** was prepared by a condensation reaction between the corresponding benzaldehyde and aniline substrates, in a literature reported procedure.[4] Liquid reagents were degassed using the freeze-pump-thaw method, with a minimum of 3 cycles, prior to being transferred into the glove box for use.

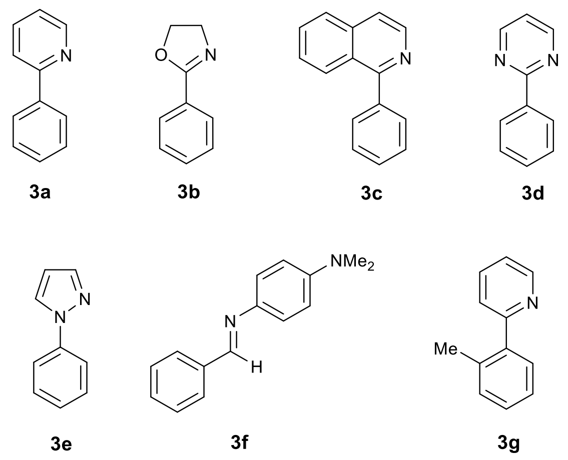

Figure S2. *N*-directing group arenes.

3.3 Aryl halide coupling partners

Substrates **2a-Cl, 2b-Cl, 2c-Cl, 2a-Br, 2b-Br, 2c-Br, 2a-I, 2b-I,** and **2c-I** were all purchased from the commercial suppliers Fluorochem, Merck, Acros and Alfa Aesar. Liquid reagents were degassed using the freeze-pump-thaw method, with a minimum of 3 cycles, prior to being transferred into the glove box for use.

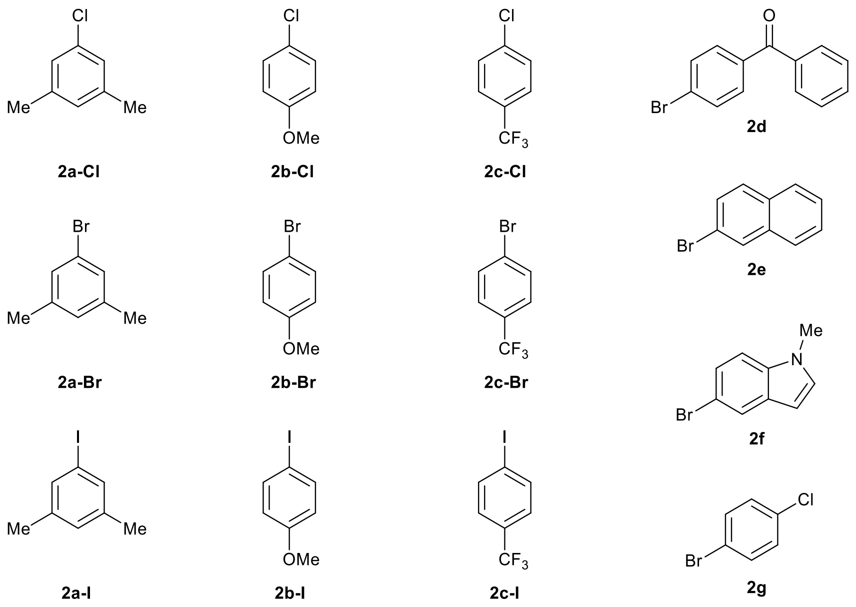

Figure S3. Simple aryl halide coupling partners.

3.4 Aryl halide coupling partners from complex molecules

Substrates **2d, 2e,** and **2f** were all purchased from commercial suppliers and used as supplied.

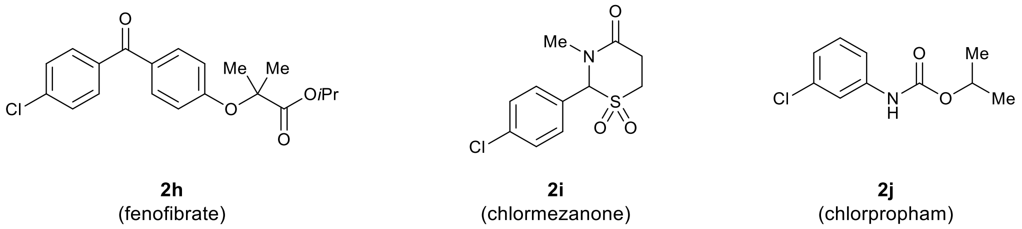

Figure S4. Bioactive aryl halide coupling partners.

3.5 Late-stage functionalisation coupling partners containing *N*-directing groups

Substrates **1h** and **1i** were purchased from Merck and Fluorochem respectively. Substrate **1j** was prepared via a Suzuki-Miyaura coupling reaction between 6-chloropurine riboside and phenylboronic acid, in a literature reported procedure.[5]

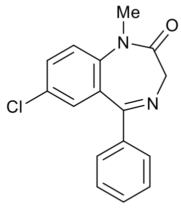

1h
(diazepam)

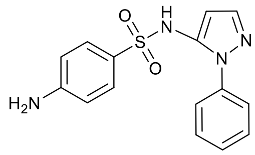

1i
(sulfaphenazole)

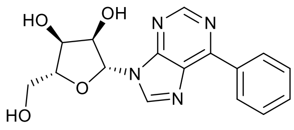

1j
(6-phenylpurine riboside)

Figure S5. Late-stage functionalisation coupling partners.

4. General Procedures

General Procedure A: Ru-catalyzed arylation of DG-containing arenes with aryl halides

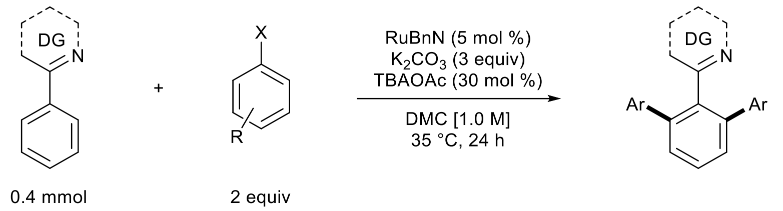

Scheme S1. Reaction scheme for General Procedure A.

All liquid reagents were degassed using the freeze-pump-thaw method, with a minimum of three cycles, prior to being transferred into the glove box for use. The ruthenium catalyst (RuBnN) was prepared using the procedure described above, and stored in the glove box for its lifetime. Potassium carbonate (K_2CO_3) and tetrabutylammonium acetate (TBAOAc) were ground using a pestle and mortar and dried in the vacuum oven for a minimum of 24 h, prior to being transferred into the glove box and stored there.

Unless otherwise stated, all reactions were set up in the glove box using 10 mL crimp-cap microwave vials, to which were added: RuBnN (10.8 mg, 0.02 mmol, 5 mol %), TBAOAc (36.2 mg, 0.12 mmol, 30 mol %), and K_2CO_3 (165.9 mg, 1.2 mmol, 3 equiv). The appropriate directing-group-containing arene (0.4 mmol, 1 equiv) and aryl halide (0.8 mmol, 2 equiv) were then added by weight (for solids) or by volume (for liquids – using an appropriate volume micro-syringe), followed by dimethyl carbonate (0.4 mL). The vials were then crimp-capped and transferred outside the glove box to be stirred on a hotplate at 35 °C for 24 hours. Upon completion, these reactions were opened to air and filtered through a small silica pad, using Et_2O as the eluent. The crude reaction mixture was then loaded onto silica gel to be purified by flash column chromatography.

General Procedure B: Ru-catalyzed arylation of DG-containing arenes with aryl halide coupling partners involving bioactive and complex substrates

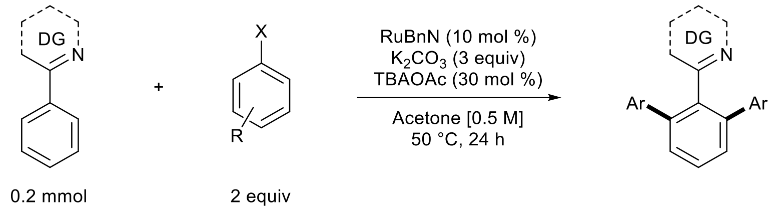

Scheme S2. Reaction scheme for General Procedure B.

All liquid reagents were degassed using the freeze-pump-thaw method, with a minimum of three cycles, prior to being transferred into the glove box for use. The ruthenium catalyst (RuBnN) was prepared using the procedure described above, and stored in the glove box for its lifetime. Potassium carbonate (K_2CO_3) and tetrabutylammonium acetate (TBAOAc) were ground using a pestle and mortar and dried in the vacuum oven for a minimum of 24 h, prior to being transferred into the glove box and stored there.

Unless otherwise stated, all reactions were set up in the glove box using 10 mL crimp-cap microwave vials, to which were added: RuBnN (10.8 mg, 0.02 mmol, 10 mol %), TBAOAc (18.1 mg, 0.06 mmol, 30 mol %), and K_2CO_3 (83.0 mg, 0.6 mmol, 3 equiv). The appropriate directing-group-containing arene (0.2 mmol, 1 equiv) and aryl halide (0.2 mmol, 1 equiv) were then added by weight (for solids) or by volume (for liquids – using an appropriate volume micro-syringe), followed by acetone (0.4 mL). The vials were then crimp-capped and transferred outside the glove box to be stirred on a hotplate at 50 °C for 24 hours. Upon completion, these reactions were loaded directly onto silica gel to be purified by flash column chromatography.

5. Product Characterisation Data

2-(3,3'',5,5''-tetramethyl-[1,1':3',1''-terphenyl]-2'-yl)pyridine, 4aa

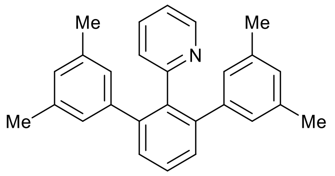

4aa

The reaction was carried out following General Procedure A, with the use of 2-phenylpyridine **1a** (62.1 mg, 0.40 mmol) and 1-bromo-3,5-dimethylbenzene **2a** (148.1 mg, 0.80 mmol, 2 equiv). The crude reaction mixture was purified by column chromatography (eluent: Hexane:EtOAc with gradient from 100:0 to 95:5) to give 2-(3,3'',5,5''-tetramethyl-[1,1':3',1''-terphenyl]-2'-yl)pyridine **4aa** (141.0 mg, 97%) as a white solid.

^{1}H NMR (500 MHz, $CDCl_3$): δ 8.34 (app. d, *J* = 4.8 Hz, 1H), 7.48 (dd, *J* = 8.7, 6.4 Hz, 1H), 7.45 – 7.40 (m, 2H), 7.32 (app. td, *J* = 7.7, 1.8 Hz, 1H), 6.93 – 6.88 (m, 2H), 6.77 (s, 2H), 6.73 (s, 4H), 2.16 (s, 12H).

^{13}C NMR (126 MHz, $CDCl_3$): δ 159.5, 148.3, 142.0, 141.6, 138.5, 137.0, 134.9, 129.3, 128.0, 127.9, 127.8, 126.9, 120.8, 21.4.

Spectroscopic data matched those previously reported.[6]

2-(3,3'',5,5''-tetramethyl-[1,1':3',1''-terphenyl]-2'-yl)-4,5-dihydrooxazole, 4ba

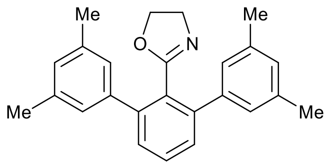

4ba

The reaction was carried out following General Procedure A, with the use of 2-phenyl-4,5-dihydrooxazole **1b** (58.9 mg, 0.40 mmol) and 1-bromo-3,5-dimethylbenzene **2a** (148.1 mg, 0.80 mmol, 2 equiv). The crude reaction mixture was purified by column chromatography (eluent: Hexane:EtOAc

with gradient from 100:0 to 90:10) to give 2-(3,3'',5,5''-tetramethyl-[1,1':3',1''-terphenyl]-2'-yl)-4,5-dihydrooxazole **3ba** (123.7 mg, 87%) as a white solid.

^{1}H NMR (400 MHz, $CDCl_3$): δ 7.50 (dd, *J* = 8.2 Hz, *J* = 7.1 Hz, 1H), 7.39 (d, *J* = 7.6 Hz, 2H), 7.12 (s, 4H), 7.00 (s, 2H), 3.95 (t, *J* = 9.4 Hz, 2H), 3.64 (t, *J* = 9.4 Hz, 2H), 2.36 (s, 12H).

^{13}C NMR (101 MHz, $CDCl_3$): δ 164.3, 142.5, 140.9, 137.4, 129.5, 128.9, 128.7, 127.4, 126.6, 67.4, 55.2, 21.4.

HRMS Mass calculated for $C_{25}H_{26}ON$ $[M+H]^+$: 356.2009, mass found: 356.2014.

1-(3',5'-dimethyl-[1,1'-biphenyl]-2-yl)isoquinoline, 3ca

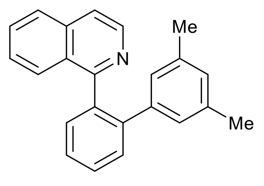

4ca

The reaction was carried out following General Procedure A, with the use of 1-phenylisoquinoline **1c** (82.1 mg, 0.40 mmol) and 1-bromo-3,5-dimethylbenzene **2a** (74.0 mg, 0.40 mmol, 1 equiv). The crude reaction mixture was purified by column chromatography (eluent: Hexane:EtOAc with gradient from 100:0 to 95:5) to give 1-(3',5'-dimethyl-[1,1'-biphenyl]-2-yl)isoquinoline **3ca** (111.4 mg, 90%) as an off-white solid.

^{1}H NMR ^{1}H NMR (400 MHz, $CDCl_3$): δ 8.53 (d, *J* = 5.7 Hz, 1H), 7.76 – 7.70 (m, 1H), 7.62 – 7.57 (m, 1H), 7.56 – 7.45 (m, 6H), 7.31 (ddd, *J* = 8.3, 6.9, 1.2 Hz, 1H), 6.68 (s, 2H), 6.61 (s, 1H), 2.07 – 1.95 (m, 6H).

^{13}C NMR (101 MHz, $CDCl_3$): δ 161.7, 142.0, 141.9, 140.9, 138.4, 137.1, 136.1, 130.6, 130.0, 129.8, 128.8, 128.2, 127.7, 127.5, 127.2, 127.2, 126.8, 126.6, 119.8, 21.1.

HRMS Mass calculated for $C_{23}H_{19}NNa$ $[M+Na]^+$: 332.1410, mass found: 332.1417.

2-(3,3'',5,5''-tetramethyl-[1,1':3',1''-terphenyl]-2'-yl)pyrimidine, 4da

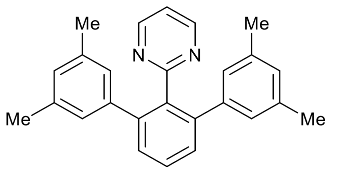

4da

The reaction was carried out following General Procedure A, with the use of 2-phenylpyrimidine **1d** (62.5 mg, 0.40 mmol) and 1-bromo-3,5-dimethylbenzene **2a** (148.1 mg, 0.80 mmol, 2 equiv). The crude reaction mixture was purified by column chromatography (eluent: Hexane:EtOAc with gradient from 100:0 to 90:10) to give 2-(3,3'',5,5''-tetramethyl-[1,1':3',1''-terphenyl]-2'-yl)pyrimidine **4da** (135.6 mg, 93%) as a white solid.

^{1}H NMR (400 MHz, $CDCl_3$): δ 8.49 (d, *J* = 5.0 Hz, 2H), 7.51 (dd, *J* = 8.6, 6.5 Hz, 1H), 7.46 – 7.41 (m, 2H), 6.93 (t, *J* = 4.9 Hz, 1H), 6.78 (s, 2H), 6.75 (s, 4H), 2.15 (s, 12H).

^{13}C NMR (126 MHz, $CDCl_3$): δ 168.6, 156.0, 141.7, 141.4, 137.7, 137.3, 129.1, 128.7, 128.2, 127.3, 117.9, 21.3.

HRMS Mass calculated for $C_{26}H_{25}N_2$ $[M+H]^+$: 365.2012, mass found: 365.2009.

1-(3,3'',5,5''-tetramethyl-[1,1':3',1''-terphenyl]-2'-yl)-1*H*-pyrazole, 4ea

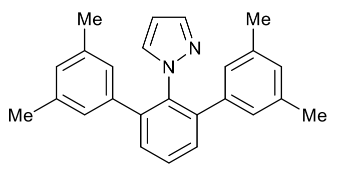

4ea

The reaction was carried out following General Procedure A, with the use of 1-phenyl-1*H*-pyrazole **1e** (57.7 mg, 0.40 mmol) and 1-bromo-3,5-dimethylbenzene **2a** (148.1 mg, 0.80 mmol, 2 equiv). The crude reaction mixture was purified by column chromatography (eluent: Hexane:EtOAc with gradient from 100:0 to 95:5) to give 1-(3,3'',5,5''-tetramethyl-[1,1':3',1''-terphenyl]-2'-yl)-1*H*-pyrazole **4ea** (133.9 mg, 95%) as a colourless oil.

^{1}H NMR (500 MHz, $CDCl_3$): δ 7.55 – 7.50 (m, 1H), 7.49 – 7.45 (m, 2H), 7.40 (d, *J* = 1.8 Hz, 1H), 7.10 (d, *J* = 2.2 Hz, 1H), 6.87 (s, 2H), 6.74 (s, 4H), 6.09 – 6.06 (m, 1H), 2.22 (s, 12H).

^{13}C NMR (126 MHz, $CDCl_3$): δ 140.7, 139.1, 138.7, 137.5, 136.5, 132.7, 129.9, 129.0, 128.9, 126.2, 105.9, 21.3.

HRMS Mass calculated for $C_{25}H_{25}N_2$ $[M+H]^+$: 353.2012, mass found: 353.2021.

3,3'',5,5''-tetramethyl-[1,1':3',1''-terphenyl]-2'-carbaldehyde, 4fa

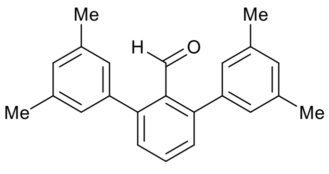

4fa

The reaction was carried out following General Procedure A, with the use of (*E*)-4-(benzylideneamino)-*N*,*N*-dimethylaniline **1f** (89.7 mg, 0.40 mmol) and 1-bromo-3,5-dimethylbenzene **2a** (148.1 mg, 0.80 mmol, 2 equiv). After 24 hours, HCl (aq. 2N, 2 mL) was added to the reaction mixture, and stirred for a further 2 h minutes, before being washed with ether. The crude reaction mixture was purified by column chromatography (eluent: Hexane:EtOAc with gradient from 100:0 to 95:5) to give 3,3'',5,5''-tetramethyl-[1,1':3',1''-terphenyl]-2'-carbaldehyde **4fa** (114.5 mg, 91%) as a white solid.

^{1}H NMR ^{1}H NMR (400 MHz, $CDCl_3$): δ 9.96 (s, 1H), 7.54 (dd, J = 8.0, 7.3 Hz, 1H), 7.35 (app. d, J = 7.6 Hz, 2H), 7.03 (s, 2H), 6.96 (s, 4H), 2.36 (s, 12H).

^{13}C NMR (101 MHz, $CDCl_3$): δ 194.0, 144.6, 139.8, 137.8, 133.4, 131.4, 130.3, 129.4, 127.6, 21.5.

Spectroscopic data matched those previously reported.[7]

2-(4,4''-dimethoxy-[1,1':3',1''-terphenyl]-2'-yl)pyridine, 4ab

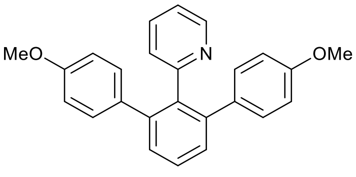

4ab

The reaction was carried out following General Procedure A, with the use of 2-phenylpyridine **1a** (62.1 mg, 0.40 mmol) and 1-bromo-4-methoxybenzene **2b-Br** (149.6 mg, 0.80 mmol, 2 equiv). The crude reaction mixture was purified by column chromatography (eluent: Hexane:EtOAc with gradient from

100:0 to 95:5) to give 2-(4,4''-dimethoxy-[1,1':3',1''-terphenyl]-2'-yl)pyridine **4ab** (142.6 mg, 97%) as a white solid.

^{1}H NMR ^{1}H NMR (400 MHz, $CDCl_3$): δ 8.35 (ddd, *J* = 4.9, 1.8, 1.0 Hz, 1H), 7.48 (dd, *J* = 8.5, 6.6 Hz, 1H), 7.43 – 7.37 (m, 2H), 7.33 (app. td, *J* = 7.7, 1.9 Hz, 1H), 7.04 – 6.98 (m, 4H), 6.92 (ddd, *J* = 7.6, 4.9, 1.2 Hz, 1H), 6.88 (app. dt, *J* = 7.8, 1.1 Hz, 1H), 6.72 – 6.66 (m, 4H), 3.74 (s, 6H).

^{13}C NMR (101 MHz, $CDCl_3$): δ 159.4, 158.2, 148.7, 141.5, 138.6, 135.1, 134.2, 130.8, 129.3, 128.2, 126.9, 120.9, 113.2, 55.2.

Spectroscopic data matched those previously reported.[8]

This reaction has also been performed on a 30 mmol scale, with the results and procedure described in section 9 of this supporting information.

2-(4,4''-bis(trifluoromethyl)-[1,1':3',1''-terphenyl]-2'-yl)pyridine, 4ac

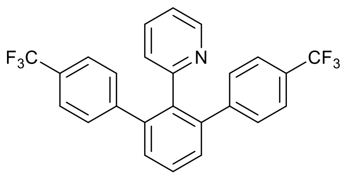

4ac

The reaction was carried out following General Procedure A, with the use of 2-phenylpyridine **1a** (62.1 mg, 0.40 mmol) and 1-bromo-4-(trifluoromethyl)benzene **2c-Br** (180.0 mg, 0.80 mmol, 2 equiv). The crude reaction mixture was purified by column chromatography (eluent: Hexane:EtOAc with gradient from 100:0 to 95:5) to give 2-(4,4''-bis(trifluoromethyl)-[1,1':3',1''-terphenyl]-2'-yl)pyridine **4ac** (170.3 mg, 96%) as a white solid.

^{1}H NMR (500 MHz, $CDCl_3$): δ 8.33 (ddd, *J* = 5.0, 1.8, 1.0 Hz, 1H), 7.58 (dd, *J* = 8.2, 7.1 Hz, 1H), 7.48 (app. d, *J* = 7.5 Hz, 2H), 7.43 (d, *J* = 8.1 Hz, 4H), 7.35 (app. td, *J* = 7.7, 1.8 Hz, 1H), 7.21 (d, *J* = 8.3 Hz, 4H), 6.98 (ddd, *J* = 7.6, 4.9, 1.2 Hz, 1H), 6.88 – 6.84 (m, 1H).

^{13}C NMR (126 MHz, $CDCl_3$): δ 157.9, 149.0, 145.1, 140.8, 138.6, 135.5, 130.1, 130.0, 128.8 (q, *J* = 32.3 Hz), 128.7, 126.8, 124.8 (q, *J* = 3.7 Hz), 124.3 (q, *J* = 271.9 Hz), 121.7.

^{19}F NMR (471 MHz, $CDCl_3$): δ -62.4 (s).

Spectroscopic data matched those previously reported.[8]

2-(4'-methoxy-3-methyl-[1,1'-biphenyl]-2-yl)pyridine, 3gb

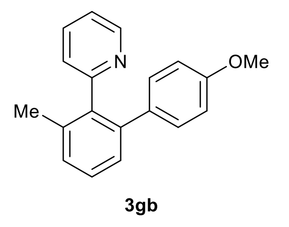

The reaction was carried out following General Procedure A, with the use of 2-(*o*-tolyl)pyridine **1g** (67.7 mg, 0.40 mmol) and either 1-chloro-4-methoxybenzene **2b-Cl** (57.0 mg, 0.40 mmol, 1 equiv), 1-bromo-4-methoxybenzene **2b-Br** (74.8 mg, 0.40 mmol, 1 equiv), or 1-iodo-4-methoxybenzene **2b-I** (93.6 mg, 0.40 mmol, 1 equiv). The crude reaction mixture was purified by column chromatography (eluent: Hexane:EtOAc with gradient from 100:0 to 95:5) to give 2-(4'-methoxy-3-methyl-[1,1'-biphenyl]-2-yl)pyridine **3gb** (93.6 mg, 85% (with **2b-Cl)**; 107.9 mg, 98% (with **2b-Br)**; and 106.7 mg, 97% (with **2b-I)**) as a viscous colourless oil.

^{1}H NMR ^{1}H NMR (500 MHz, $CDCl_3$): δ 8.63 (ddd, *J* = 4.8, 1.8, 0.9 Hz, 1H), 7.47 (app. td, *J* = 7.7, 1.8 Hz, 1H), 7.34 (app. t, *J* = 7.6 Hz, 1H), 7.28 – 7.23 (m, 2H), 7.10 (ddd, *J* = 7.6, 4.9, 1.2 Hz, 1H), 7.01 – 6.96 (m, 2H), 6.88 (app. dt, *J* = 7.8, 1.1 Hz, 1H), 6.69 – 6.65 (m, 2H), 3.73 (s, 3H), 2.17 (s, 3H).

^{13}C NMR (126 MHz, $CDCl_3$): δ 159.9, 158.2, 149.0, 140.9, 139.5, 136.8, 135.9, 134.2, 130.8, 129.2, 128.1, 127.7, 125.8, 121.4, 113.2, 55.2, 20.6.

Spectroscopic data matched those previously reported.[9]

2-(3-methyl-4'-(trifluoromethyl)-[1,1'-biphenyl]-2-yl)pyridine, 3gc

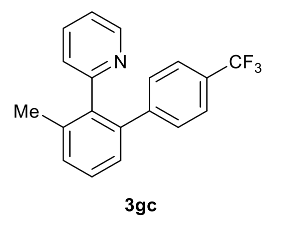

The reaction was carried out following General Procedure A, with the use of 2-(*o*-tolyl)pyridine **1g** (67.7 mg, 0.40 mmol) and either 1-chloro-4-(trifluoromethyl)benzene **2c-Cl** (72.2 mg, 0.40 mmol, 1 equiv), 1-bromo-4-(trifluoromethyl)benzene **2c-Br** (90.0 mg, 0.40 mmol, 1 equiv), or 1-iodo-4-

(trifluoromethyl)benzene **2c-I** (108.8 mg, 0.40 mmol, 1 equiv). The crude reaction mixture was purified by column chromatography (eluent: Hexane:EtOAc with gradient from 100:0 to 95:5) to give 2-(3-methyl-4'-(trifluoromethyl)-[1,1'-biphenyl]-2-yl)pyridine **3gc** (121.6 mg, 97% (with **2c-Cl)**; 120.3 mg, 96% (with **2c-Br)**; and 114.0 mg, 91% (with **2c-I)**) as an off-white solid.

^{1}H NMR ^{1}H NMR (500 MHz, $CDCl_3$): δ 8.63 (ddd, *J* = 5.0, 2.0, 1.0 Hz, 1H), 7.49 (app. td, *J* = 7.7, 1.8 Hz, 1H), 7.41 – 7.36 (m, 3H), 7.34 (d, *J* = 6.6 Hz, 1H), 7.28 – 7.22 (m, 1H), 7.19 (d, *J* = 8.0 Hz, 2H), 7.13 (ddd, *J* = 7.6, 4.9, 1.2 Hz, 1H), 6.91 – 6.87 (m, 1H), 2.19 (s, 3H).

^{13}C NMR (126 MHz, $CDCl_3$): δ 159.0, 149.1, 145.4, 139.8, 139.3, 137.0, 136.0, 130.1, 129.9, 128.4 (q, *J* = 32.4 Hz), 128.2, 127.5, 125.5, 124.6 (q, *J* = 3.9 Hz), 124.2 (q, *J* = 272.1 Hz), 121.6, 20.4.

^{19}F NMR (471 MHz, $CDCl_3$): δ -62.4 (s).

Spectroscopic data matched those previously reported.[10]

(3'-Methyl-2'-(pyridin-2-yl)-[1,1'-biphenyl]-4-yl)(phenyl)methanone, 3gd

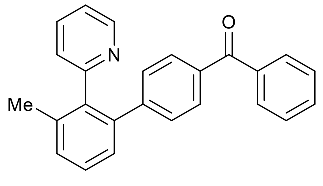

3gd

The reaction was carried out following General Procedure A, with the use of 2-(*o*-tolyl)pyridine **1g** (67.7 mg, 0.40 mmol) and (4-bromophenyl)(phenyl)methanone **2d** (104.4 mg, 0.40 mmol, 1 equiv). The crude reaction mixture was purified by column chromatography (eluent: Hexane:EtOAc with gradient from 100:0 to 95:5) to give (3'-methyl-2'-(pyridin-2-yl)-[1,1'-biphenyl]-4-yl)(phenyl)methanone **3gd** (125.8 mg, 90%).

^{1}H NMR ^{1}H NMR (500 MHz, $CDCl_3$): δ 8.62 (ddd, J = 4.9, 1.9, 0.9 Hz, 1H), 7.76 – 7.69 (m, 2H), 7.63 – 7.58 (m, 2H), 7.57 – 7.51 (m, 1H), 7.50 – 7.40 (m, 3H), 7.38 (d, J = 7.5 Hz, 1H), 7.36 – 7.27 (m, 2H), 7.23 – 7.16 (m, 2H), 7.11 (ddd, J = 7.7, 4.9, 1.3 Hz, 1H), 6.91 (d, J = 7.8 Hz, 1H), 2.20 (s, 3H).

^{13}C NMR (126 MHz, $CDCl_3$): δ 196.5, 159.2, 149.1, 146.3, 140.2, 139.4, 137.8, 137.1, 136.0, 135.3, 132.3, 130.2, 130.0, 129.7, 129.6, 128.3, 127.5, 125.7, 121.7, 20.5.

HRMS Mass calculated for $C_{25}H_{20}NO$ $[M+H]^+$: 350.1539, mass found: 350.1536.

2-(2-Methyl-6-(naphthalen-2-yl)phenyl)pyridine, 3ge

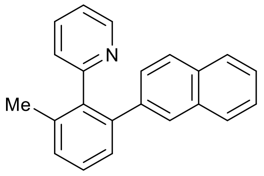

3ge

The reaction was carried out following General Procedure A, with the use of 2-(*o*-tolyl)pyridine **1g** (67.7 mg, 0.40 mmol) and 2-bromonaphthalene **2e** (82.8 mg, 0.40 mmol, 1 equiv). The crude reaction mixture was purified by column chromatography (eluent: Hexane:EtOAc with gradient from 100:0 to 95:5) to give 2-(2-methyl-6-(naphthalen-2-yl)phenyl)pyridine **3ge** (99.2mg, 84%).

^{1}H NMR ^{1}H NMR (400 MHz, $CDCl_3$): δ 8.66 (d, J = 4.9 Hz, 1H), 7.78 – 7.68 (m, 2H), 7.67 (d, J = 1.8 Hz, 1H), 7.58 (d, J = 8.5 Hz, 1H), 7.47 – 7.32 (m, 6H), 7.18 (dd, J = 8.5, 1.9 Hz, 1H), 7.09 – 7.01 (m, 1H), 6.92 (d, J = 7.8 Hz, 1H), 2.25 (s, 3H).

^{13}C NMR (101 MHz, $CDCl_3$): δ 159.6, 149.0, 141.2, 139.6, 139.4, 136.9, 135.9, 133.2, 131.9, 129.6, 128.5, 128.2, 128.1, 128.0(5), 128.0(1), 127.5, 127.0, 125.9, 125.8, 125.7, 121.4, 20.6.

HRMS Mass calculated for $C_{25}H_{25}N_2$ $[M+H]^+$: 353.2012, mass found: 353.2021.

Spectroscopic data matched those previously reported.[11]

1-Methyl-5-(3-methyl-2-(pyridin-2-yl)phenyl)-1*H*-indole, 3gf

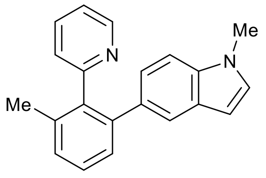

3gf

The reaction was carried out following General Procedure A, with the use of 2-(*o*-tolyl)pyridine **1g** (67.7 mg, 0.40 mmol) and 5-bromo-1-methyl-1H-indole **2f** (84.0 mg, 0.40 mmol, 1 equiv). The crude reaction mixture was purified by column chromatography (eluent: Hexane:EtOAc with gradient from 100:0 to 95:5) to give 1-methyl-5-(3-methyl-2-(pyridin-2-yl)phenyl)-1*H*-indole **3gf** (85.9mg, 72%).

^{1}H NMR ^{1}H NMR (400 MHz, $CDCl_3$): δ 8.68 (d, J = 4.3 Hz, 1H), 7.47 (s, 1H), 7.42 – 7.28 (m, 4H), 7.09 – 7.01 (m, 2H), 6.98 (d, J = 2.6 Hz, 1H), 6.93 (dd, J = 14.2, 8.0 Hz, 2H), 6.41 (d, J = 2.9 Hz, 1H), 3.68 (s, 3H), 2.25 (s, 3H).

^{13}C NMR (101 MHz, $CDCl_3$): δ ^{13}C NMR (101 MHz, $CDCl_3$) δ 160.2, 148.8, 142.4, 139.6, 136.6, 135.7, 135.4, 133.0, 129.0, 128.8, 128.3, 128.1, 127.9, 125.7, 124.0, 121.9, 121.1, 108.2, 101.0, 32.8, 20.6.

HRMS Mass calculated for $C_{21}H_{19}N_2$ $[M+H]^+$: 299.1543, mass found: 299.1553.

2-(4'-Chloro-3-methyl-[1,1'-biphenyl]-2-yl)pyridine, 3gg

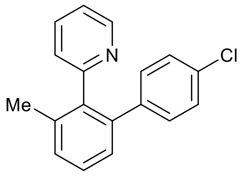

3gg

The reaction was carried out following General Procedure A, with the use of 2-(*o*-tolyl)pyridine **1g** (67.7 mg, 0.40 mmol) and 1-bromo-4-chlorobenzene **2g** (76.6 mg, 0.40 mmol, 1 equiv). The crude reaction mixture was purified by column chromatography (eluent: Hexane:EtOAc with gradient from 100:0 to 95:5) to give 2-(4'-chloro-3-methyl-[1,1'-biphenyl]-2-yl)pyridine **3gg** (101.8 mg, 91%).

^{1}H NMR ^{1}H NMR (400 MHz, $CDCl_3$) δ 8.66 – 8.58 (m, 1H), 7.48 (app. td, J = 7.7, 1.9 Hz, 1H), 7.38 – 7.27 (m, 2H), 7.23 (d, J = 1.8 Hz, 1H), 7.14 – 7.07 (m, 3H), 7.03 – 6.98 (m, 2H), 6.89 (d, J = 1.2 Hz, 1H), 2.18 (s, 3H).

^{13}C NMR (101 MHz, $CDCl_3$): δ 159.4, 149.1, 140.2, 140.0, 139.4, 136.9, 136.0, 132.4, 131.0, 129.8, 128.2, 127.9, 127.5, 125.6, 121.6, 20.5.

HRMS Mass calculated for $C_{18}H_{15}NCl$ $[M+H]^+$: 280.0888, mass found: 280.0896.

Isopropyl 2-methyl-2-(4-(3'-methyl-2'-(pyridin-2-yl)-[1,1'-biphenyl]-4-carbonyl)phenoxy)propanoate, 3gh

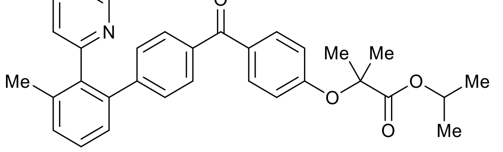

3gh

The reaction was carried out following General Procedure B, with the use of 2-(*o*-tolyl)pyridine **1g** (38.8 mg, 0.20 mmol) and isopropyl 2-(4-(4-chlorobenzoyl)phenoxy)-2-methylpropanoate (fenofibrate) **2h** (72.2 mg, 0.20 mmol, 1 equiv). The crude reaction mixture was purified by column chromatography (eluent: Hexane:EtOAc:CH_2Cl_2 with gradient from 100:0:0 to 50:25:25) to give isopropyl 2-methyl-2-(4-(3'-methyl-2'-(pyridin-2-yl)-[1,1'-biphenyl]-4-carbonyl)phenoxy)propanoate **3gh** (79.0 mg, 80%) as an off-white solid.

^{1}H NMR ^{1}H NMR (500 MHz, $CDCl_3$): δ 8.62 (ddd, *J* = 4.9, 1.8, 1.0 Hz, 1H), 7.71 – 7.66 (m, 2H), 7.57 – 7.51 (m, 2H), 7.48 (app. td, *J* = 7.7, 1.8 Hz, 1H), 7.40 (app. t, *J* = 7.6 Hz, 1H), 7.35 – 7.32 (m, 1H), 7.32 – 7.29 (m, 1H), 7.19 – 7.15 (m, 2H), 7.11 (ddd, *J* = 7.6, 4.9, 1.2 Hz, 1H), 6.91 (app. dt, *J* = 7.9, 1.1 Hz, 1H), 6.85 – 6.81 (m, 2H), 5.08 (hept, *J* = 6.3 Hz, 1H), 2.19 (s, 3H), 1.65 (s, 6H), 1.19 (d, *J* = 6.3 Hz, 6H).

^{13}C NMR (126 MHz, $CDCl_3$): δ 195.5, 173.3, 159.5, 159.2, 149.1, 145.8, 140.4, 139.4, 137.1, 136.1, 135.9, 132.1, 130.8, 130.2, 129.6, 129.5, 128.3, 127.6, 125.7, 121.7, 117.2, 79.4, 69.4, 25.5, 21.6, 20.6.

Spectroscopic data matched those previously reported.[6]

3-Methyl-2-(3'-methyl-2'-(pyridin-2-yl)-[1,1'-biphenyl]-4-yl)-1,3-thiazinan-4-one 1,1-dioxide, 3gi

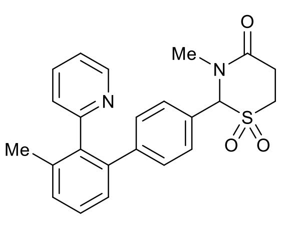

3gi

The reaction was carried out following General Procedure B, with the use of 2-(*o*-tolyl)pyridine **1g** (38.8 mg, 0.20 mmol) and 2-(4-chlorophenyl)-3-methyl-1,3-thiazinan-4-one 1,1-dioxide (chlormezanone) **2i** (54.7 mg, 0.20 mmol, 1 equiv). The crude reaction mixture was purified by column chromatography (eluent: CH_2Cl_2:EtOAc with gradient from 100:0 to 40:60) to give 3-methyl-2-(3'-methyl-2'-(pyridin-2-yl)-[1,1'-biphenyl]-4-yl)-1,3-thiazinan-4-one 1,1-dioxide **3gi** (66.7 mg, 82%) as a off-white waxy solid.

^{1}H NMR ^{1}H NMR (400 MHz, $CDCl_3$): δ 8.64 – 8.57 (m, 1H), 7.45 (app. td, *J* = 7.7, 1.8 Hz, 1H), 7.39 (app. t, *J* = 7.5 Hz, 1H), 7.33 (ddd, *J* = 7.6, 1.5, 0.8 Hz, 1H), 7.30 – 7.24 (m, 1H), 7.21 –

7.08 (m, 5H), 6.89 (d, *J* = 7.7 Hz, 1H), 5.16 (d, *J* = 2.0 Hz, 1H), 3.31 – 2.95 (m, 4H), 2.89 (s, 3H), 2.19 (s, 3H).

^{13}C NMR (101 MHz, $CDCl_3$): δ 166.2, 159.3, 149.1, 144.3, 140.0, 139.5, 137.1, 135.9, 130.6, 130.2, 128.4, 128.0, 127.5, 127.5, 125.8, 121.7, 80.5, 43.4, 36.3, 30.7, 20.6.

Spectroscopic data matched those previously reported.[6]

Isopropyl (3'-methyl-2'-(pyridin-2-yl)-[1,1'-biphenyl]-3-yl)carbamate, 3gj

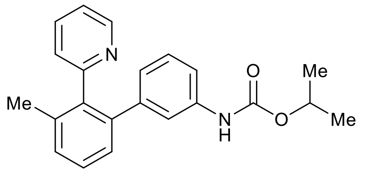

3gj

The reaction was carried out following General Procedure B, with the use of 2-(*o*-tolyl)pyridine **1g** (38.8 mg, 0.20 mmol) and isopropyl (3-chlorophenyl)carbamate (chlorpropham) **2j** (42.7 mg, 0.20 mmol, 1 equiv). The crude reaction mixture was purified by column chromatography (eluent: Hexane:EtOAc:CH_2Cl_2 with gradient from 100:0:0 to 50:25:25) to give isopropyl (3'-methyl-2'-(pyridin-2-yl)-[1,1'-biphenyl]-3-yl)carbamate **3gj** (60.2 mg, 87%) as a white solid.

^{1}H NMR ^{1}H NMR (400 MHz, $CDCl_3$): δ 8.62 (ddd, *J* = 4.9, 1.9, 1.0 Hz, 1H), 7.46 (app. td, *J* = 7.7, 1.9 Hz, 1H), 7.36 – 7.21 (m, 4H), 7.09 (ddd, *J* = 7.6, 4.9, 1.2 Hz, 1H), 7.04 – 6.98 (m, 2H), 6.90 (app. dt, *J* = 7.8, 1.1 Hz, 1H), 6.67 (app. dt, *J* = 7.8, 1.3 Hz, 1H), 6.53 (s, 1H), 4.98 (hept, *J* = 6.2 Hz, 1H), 2.17 (s, 3H), 1.27 (d, *J* = 6.3 Hz, 6H).

^{13}C NMR (126 MHz, $CDCl_3$): δ 159.6, 153.2, 149.0, 142.6, 140.9, 139.4, 137.7, 136.8, 135.9, 129.6, 128.3, 128.1, 127.7, 125.7, 124.9, 121.5, 119.8, 116.6, 68.8, 22.2, 20.6.

Spectroscopic data matched those previously reported.[6]

7-Chloro-1-methyl-5-(3,3'',5,5''-tetramethyl-[1,1':3',1''-terphenyl]-2'-yl)-1,3-dihydro-2*H*-benzo[*e*][1,4]diazepin-2-one, 4ha

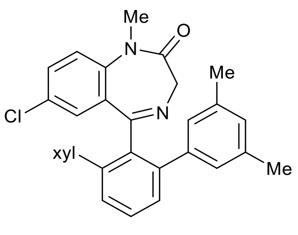

4ha

The reaction was carried out following General Procedure B, with the use of 7-chloro-1-methyl-5-phenyl-1,3-dihydro-2*H*-benzo[*e*][1,4]diazepin-2-one (diazepam) **1h** (56.9 mg, 0.20 mmol) and 1-bromo-3,5-dimethylbenzene **2a** (148.1 mg, 0.40 mmol, 2 equiv) for 48 h. The crude reaction mixture was purified by column chromatography (eluent: Hexane:Et_2O with gradient from 100:0 to 50:50) to give 7-chloro-1-methyl-5-(3,3'',5,5''-tetramethyl-[1,1':3',1''-terphenyl]-2'-yl)-1,3-dihydro-2*H*-benzo[*e*][1,4]diazepin-2-one **4ha** (90.7 mg, 92%) as a white solid.

^{1}H NMR ^{1}H NMR (400 MHz, $CDCl_3$): δ 7.52 – 7.42 (m, 2H), 7.23 (dd, *J* = 8.8, 2.5 Hz, 1H), 7.17 (dd, *J* = 6.7, 2.2 Hz, 1H), 7.08 – 7.01 (m, 3H), 6.92 (s, 1H), 6.80 (d, *J* = 8.8 Hz, 1H), 6.75 (s, 1H), 6.55 (s, 2H), 4.51 (d, *J* = 10.8 Hz, 1H), 3.31 (d, *J* = 10.8 Hz, 1H), 2.89 (s, 3H), 2.29 (s, 6H), 2.13 (s, 6H).

^{13}C NMR (101 MHz, $CDCl_3$): δ 169.3, 168.4, 142.7, 142.4, 141.4, 141.1, 140.8, 137.3, 137.1, 136.8, 132.0, 130.6, 129.7, 129.2, 128.9, 128.7, 128.6, 128.2, 127.6, 127.4, 121.5, 56.1, 34.6, 21.5, 21.3.

Spectroscopic data matched those previously reported.[6]

4-Amino-*N*-(1-(3',5'-dimethyl-[1,1'-biphenyl]-2-yl)-1*H*-pyrazol-5-yl)benzenesulfonamide, 3ia

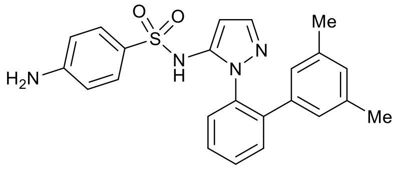

3ia

The reaction was carried out following General Procedure B, with the use of 4-amino-*N*-(1-phenyl-1*H*-pyrazol-5-yl)benzenesulfonamide (sulfaphenazole) **1i** (62.9 mg, 0.20 mmol) and 1-bromo-3,5-

dimethylbenzene **2a** (74.0 mg, 0.20 mmol, 1 equiv) for 48 h. The crude reaction mixture was purified by column chromatography (eluent: CH_2Cl_2:Et_2O with slow gradient from 100:0 to 90:10) to give 4-amino-*N*-(1-(3',5'-dimethyl-[1,1'-biphenyl]-2-yl)-1*H*-pyrazol-5-yl)benzenesulfonamide **3ia** (71.1 mg, 85%) as an off-white solid.

^{1}H NMR ^{1}H NMR (400 MHz, $CDCl_3$): δ 7.55 (d, *J* = 2.0 Hz, 1H), 7.48 – 7.42 (m, 2H), 7.29 (ddd, *J* = 7.9, 5.9, 3.0 Hz, 1H), 7.18 – 7.12 (m, 2H), 6.98 – 6.91 (m, 2H), 6.64 (s, 2H), 6.47 – 6.40 (m, 2H), 6.11 (d, *J* = 2.0 Hz, 1H), 5.50 (s, 1H), 4.13 (s, 2H), 2.22 (s, 6H).

^{13}C NMR (101 MHz, $CDCl_3$): δ 151.0, 140.1, 139.0, 138.1, 137.3, 135.3, 134.9, 130.3, 130.1, 129.5, 129.3, 128.5, 128.4, 126.6, 125.9, 113.9, 102.5, 21.4.

Spectroscopic data matched those previously reported.[6]

(2*R*,3*S*,4*R*,5*R*)-2-(hydroxymethyl)-5-(6-(3,3'',5,5''-tetramethyl-[1,1':3',1''-terphenyl]-2'-yl)-9*H*-purin-9-yl)tetrahydrofuran-3,4-diol, 4ja

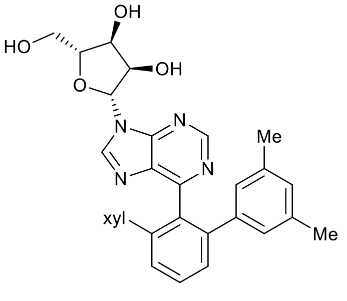

4ja

The reaction was carried out following General Procedure B, with the use of (2*R*,3*S*,4*R*,5*R*)-2-(hydroxymethyl)-5-(6-phenyl-9*H*-purin-9-yl)tetrahydrofuran-3,4-diol **1j** (65.7 mg, 0.20 mmol) and 1-bromo-3,5-dimethylbenzene **2a** (148.1 mg, 0.40 mmol, 2 equiv) for 48 h. The crude reaction mixture was purified by column chromatography (eluent: CH_2Cl_2:MeOH with slow gradient from 100:0 to 95:5) to give (2*R*,3*S*,4*R*,5*R*)-2-(hydroxymethyl)-5-(6-(3,3'',5,5''-tetramethyl-[1,1':3',1''-terphenyl]-2'-yl)-9*H*-purin-9-yl)tetrahydrofuran-3,4-diol **4ja** (54.7 mg, 51%) as a light-brown solid.

^{1}H NMR ^{1}H NMR (400 MHz, $CDCl_3$): δ 8.65 (s, 1H), 7.80 (s, 1H), 7.51 (app. t, *J* = 7.6 Hz, 1H), 7.44 – 7.38 (m, 2H), 7.26 (s, 1H), 6.76 – 6.63 (m, 6H), 5.67 (d, *J* = 6.9 Hz, 1H), 5.44 (dd, *J* = 10.8, 2.5 Hz, 1H), 4.77 – 4.69 (m, 1H), 4.31 (d, *J* = 5.1 Hz, 1H), 4.23 – 4.18 (m, 1H), 3.88 – 3.78 (m, 2H), 3.68 – 3.59 (m, 1H), 3.48 (br. s, 1H), 2.06 (s, 6H), 2.02 (s, 6H).

^{13}C NMR (101 MHz, $CDCl_3$): δ 161.1, 150.8, 149.4, 144.3, 142.31, 142.29, 140.7, 140.6, 137.2, 137.1, 135.7, 132.6, 129.4, 129.2, 128.6, 128.5, 127.2, 127.1, 91.4, 87.7, 73.9, 71.9, 62.9, 21.2, 21.1.

Spectroscopic data matched those previously reported.[6]

6. Reaction Optimization

The reactions below were set up in the glove box using 10 mL crimp-cap microwave vials, to which were added: RuBnN, TBAOAc, and K_2CO_3 in the quantities described. The directing-group-containing arene 2-phenylpyridine **1a** (1 equiv) and aryl halide 1-bromo-3,5-dimethylbenzene **2a** (2 equiv) were then added by using an appropriate volume syringe, followed by the solvent DMC. The vials were then crimp-capped and transferred outside the glove box to be stirred on a hotplate at the desired temperature for a set time period. Upon completion, the reactions were opened to air and quenched by the addition of the internal standard, hexadecane, in 2% Pyridine in Et_2O. After thorough mixing, a portion of the reaction mixture was filtered through a silica plug into a vial suitable for GC-FID, using Et_2O as the eluent. The samples were then run on the GC-FID to determine the distribution of products, which are as reported below.

Variation of catalyst loading

Table S1. Varying catalyst loading.

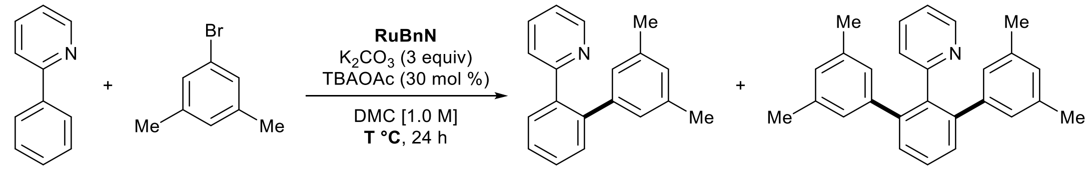

1a 0.8 mmol; **2a** 2 equiv; **3aa**; **4aa**

Entry	RuBnN (mol %)	Temp (°C)	Yield 3aa (%)	Yield 4aa (%)
**1**	5	25	0	95
**2**	2.5	25	7	63
**3**	2.5	35	0	96
**4**	1	35	4	28
**5**	1	50	1	95
**5**	0.5	50	5	76
**7**	0.25	50	8	32
**8**	0.25	70	3	87

Variation of reaction time

Table S2. Varying reaction time.

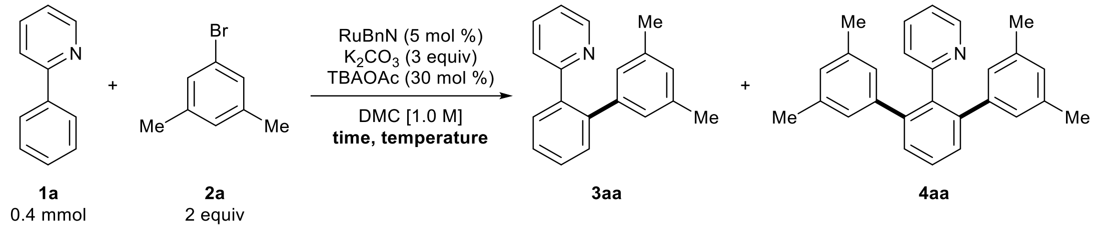

Entry	Time (h)	Temp (°C)	Yield 3aa (%)	Yield 4aa (%)
**1**	2.5	50	0	99
**2**	1.5	50	3	94
**3**	0.5	50	14	45
**4**	0.5	70	3	92

Variation of reaction concentration

Table S3. Varying reaction concetration.

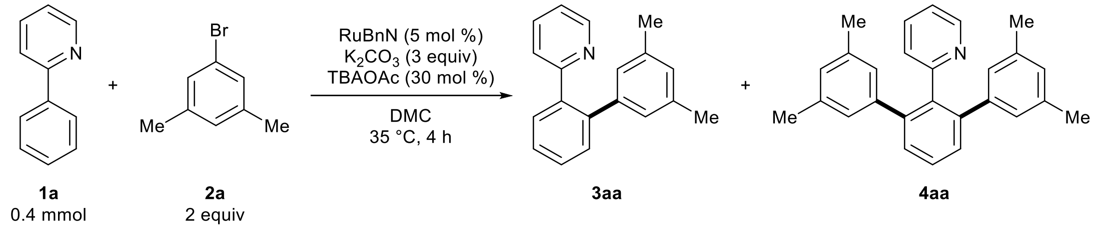

Entry	Concentration (M)	Yield 3aa (%)	Yield 4aa (%)
**1**	0.5	8	50
**2**	1.0	9	68
**3**	1.5	10	59

Variation of additive loading

Table S4. Varying additive (TBAOAc) loading.

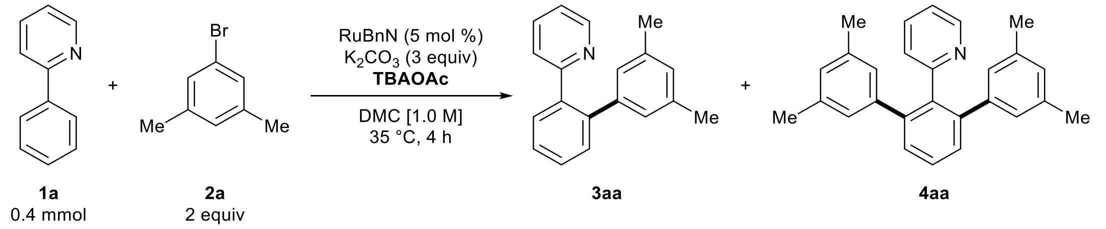

Entry	TBAOAc (mol %)	Yield 3aa (%)	Yield 4aa (%)
**1**	10	10	46
**2**	30	12	52
**3**	50	11	50

Variation of reaction additive

Table S5. Varying reaction additive.

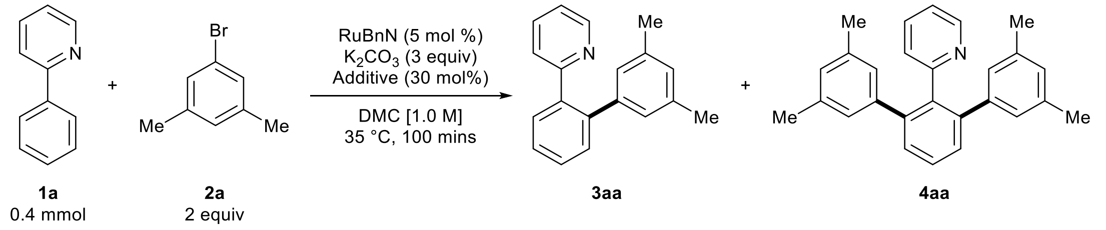

Entry	Solvent	Additive	Yield 3aa (%)	Yield 4aa (%)
**1**	NMP	KOAc	7	19
**2**	DMC	KOAc	3	2
**3**	DMC	KCO_2Ad	2	2
**4**	DMC	KCO_2Mes	3	1
**5**	DMC	Levulinic acid	4	2
**6**	DMC	KOAc (+pivalamide 30 mol %)	2	2
**7**	DMC	TBAOAc	4	21
**8**	DMC	TEAOAc	5	17
**9**	DMC	TMAOAc	6	16
**10**	DMC	TBAOAc (8 h reaction time)	0	97

7. Solvent Screen

The reactions were set up in the glove box using 10 mL crimp-cap microwave vials, to which were added: RuBnN (10.8 mg, 0.02 mmol, 5 mol %), TBAOAc (36.2 mg, 0.12 mmol, 30 mol %), and K_2CO_3 (165.9 mg, 1.2 mmol, 3 equiv). The directing-group-containing arene 2-phenylpyridine **1a** (64.1 mg, 0.4 mmol, 1 equiv) and aryl halide 1-bromo-3,5-dimethylbenzene **2a** (148.1 mg, 0.8 mmol, 2 equiv) were then added by using an appropriate volume syringe, followed by the solvent (0.4 mL). The vial was then crimp-capped and transferred outside the glove box to be stirred on a hotplate at 25 °C for either 2 or 24 hours. Upon completion, the reactions were opened to air and quenched by the addition of the internal standard hexadecane in 2% Pyridine in Et_2O. After thorough mixing, a portion of the reaction mixture was filtered through a silica plug into a vial suitable for GC-FID, using Et_2O as the eluent. The samples were then run on the GC-FID to determine the distribution of products, which are as reported below.

Table S6. Screen of green solvents and graphic representation.

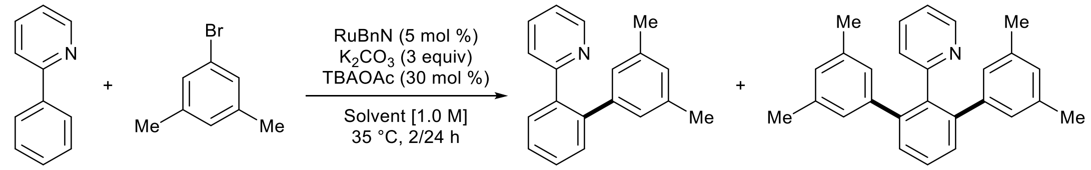

1a 0.4 mmol | **2a** 2 equiv | **3aa** | **4aa**

Entry	Solvent	Time (h)	Yield 3aa (%)	Yield 4aa (%)
**1**	Dimethyl carbonate (DMC)	2	7	38
**2**	Dimethyl carbonate (DMC)	24	0	97
**3**	Diethyl carbonate (DEC)	2	13	42
**4**	Diethyl carbonate (DEC)	24	0	96
**5**	Acetone	2	12	55
**6**	Acetone	24	0	98
**7**	Ethyl acetate (EtOAc)	2	12	51
**8**	Ethyl acetate (EtOAc)	24	0	92
**9**	*n*-Propyl acetate (*n*PrOAc)	2	12	49
**10**	*n*-Propyl acetate (*n*PrOAc)	24	0	99
**11**	*i*-Propyl acetate (*i*PrOAc)	2	14	50
**12**	*i*-Propyl acetate (*i*PrOAc)	24	0	97
**13**	γ-Butyralactone	2	3	33
**14**	γ-Butyralactone	24	0	95
**15**	ε-Caprolactone	2	11	41

**16**	ε-Caprolactone	24	0	87
**17**	Ethyl lactate	2	9	12
**18**	Ethyl lactate	24	10	71
**19**	3-Methyl-2-butanol	2	10	13
**20**	3-Methyl-2-butanol	24	0	99
**21**	*i*PrOH	2	6	14
**22**	*i*PrOH	24	0	98
**23**	Cyrene	2	9	31
**24**	Cyrene	24	0	96
**25**	Cylopentyl methyl ether (CPME)	2	13	38
**26**	Cylopentyl methyl ether (CPME)	24	0	89
**27**	*tert*-Butyl methyl ether (TBME)	2	13	38
**28**	*tert*-Butyl methyl ether (TBME)	24	0	98
**29**	2-Methyltetrahydrofuran (2-MeTHF)	2	12	45
**30**	2-Methyltetrahydrofuran (2-MeTHF)	24	0	98
**31**	Sulfolane	2	9	25
**32**	Sulfolane	24	0	96
**33**	DMPU	2	6	40
**34**	DMPU	24	0	93
**35**	Anisole	2	14	35
**36**	Anisole	24	0	91
**37**	2-Methylanisole	2	15	38
**38**	2-Methylanisole	24	0	96
**39**	Heptane	2	10	16
**40**	Heptane	24	0	95
**41**	Water (H_2O)	2	8	11
**42**	Water (H_2O)	24	27	57
**43**	Neat (No solvent)	2	14	33
**44**	Neat (No solvent)	24	16	74

The table displayed in the main manuscript contains the comparison between each solvent in terms of total arylations (ie. **3aa** + **2** × **4aa**):

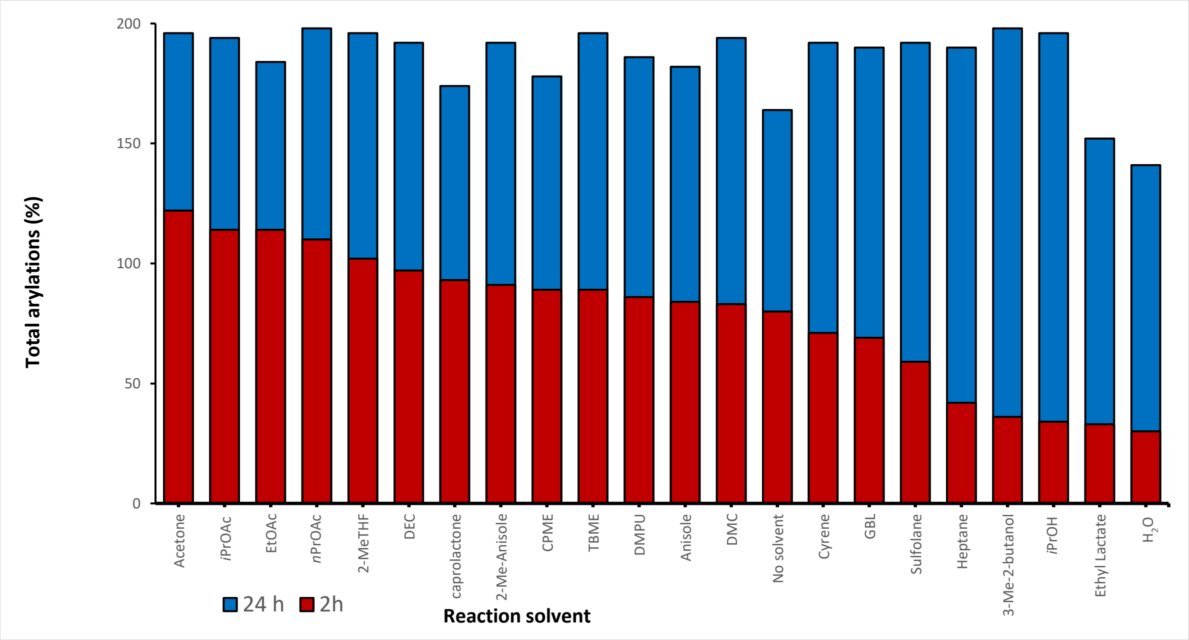

For reactions performed with no solvent (entries 43 and 44), these were performed in the same manner as described above, without the addition of solvent at the final step.

8. Reaction Comparisons

For the following reactions, liquid reagents **1a** and **2a** were degassed using the freeze-pump-thaw method, with a minimum of three cycles, prior to being transferred into the glove box for use. The ruthenium catalyst (RuBnN) was prepared using the procedure described in section 2 and stored in the glove box for use. Potassium carbonate (K_2CO_3) and tetrabutylammonium acetate (TBAOAc) were ground using a pestle and mortar and dried in the vacuum oven for a minimum of 24 h, prior to being transferred into the glove box and stored there for use. Dry DMC solvent was purchased from Merck and degassed using the freeze-pump-thaw method, with a minimum of three cycles, prior to being transferred into the glove box for use.

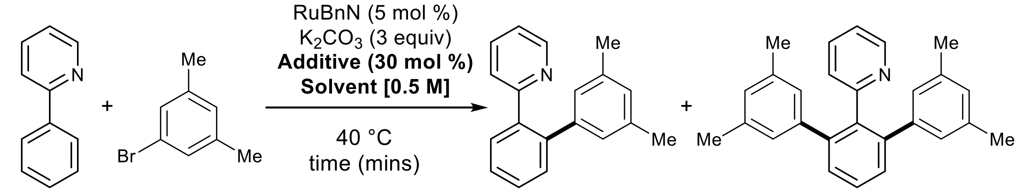

1a 0.4 mmol **2a** 2 equiv **3aa** **4aa**

Conditions	Solvent	Additive
A	NMP	KOAc
B	Acetone	KOAc
C	Acetone	TBAOAc

Scheme S3. Scheme of reaction conditions for rate comparison.

Additive TBAOAc (36.2 mg, 0.12 mmol, 30 mol %) and base K_2CO_3 (165.9 mg, 1.2 mmol, 3 equiv) were weighed out in the glovebox into 3 x 10 mL microwave vials. Stock solutions in NMP or Acetone were prepared for 2-phenylpyridine **1a**, 1-bromo-3,5-dimethylbenzene **2a,** and internal standard hexadecane, and these were added to the vial using the appropriate volume microsyringe. NMP or Acetone was then added to make the volume to 0.7 mL and the vial was capped with a B14 suba-seal. The reaction was then heated at 40 °C inside the glove box for 20 min with a stirring rate of 500 rpm, before a solution of RuBnN in NMP or Acetone (100 µL) was added at 0 min to start the reaction. Aliquots of approximately 20 µL were then taken throughout the reaction at specified time points. Each aliquot was added to approximately 0.5 mL of a solution of 2% pyridine in EtOAc (v/v), before being removed from the glove box to be passed through a short plug of silica into a GC vial ready for analysis. The samples were then analysed by GC-FID, using hexadecane as the internal standard.

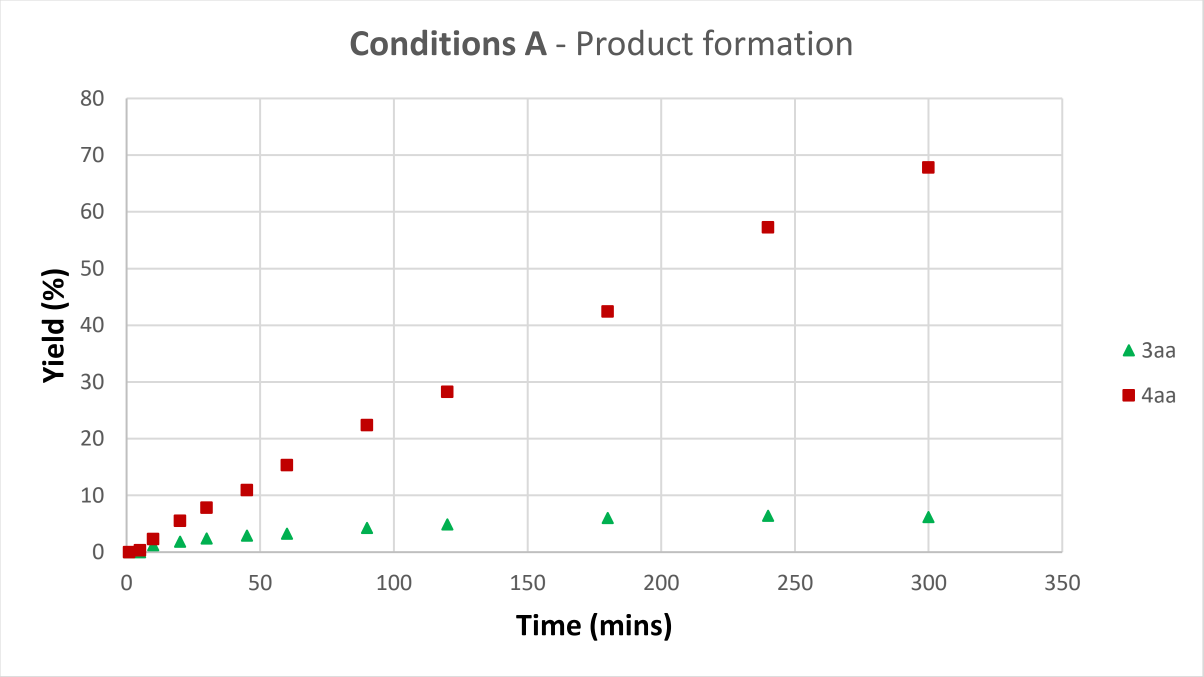

Figure S6. Graph of formation of **3aa** and **4aa** under conditions A.

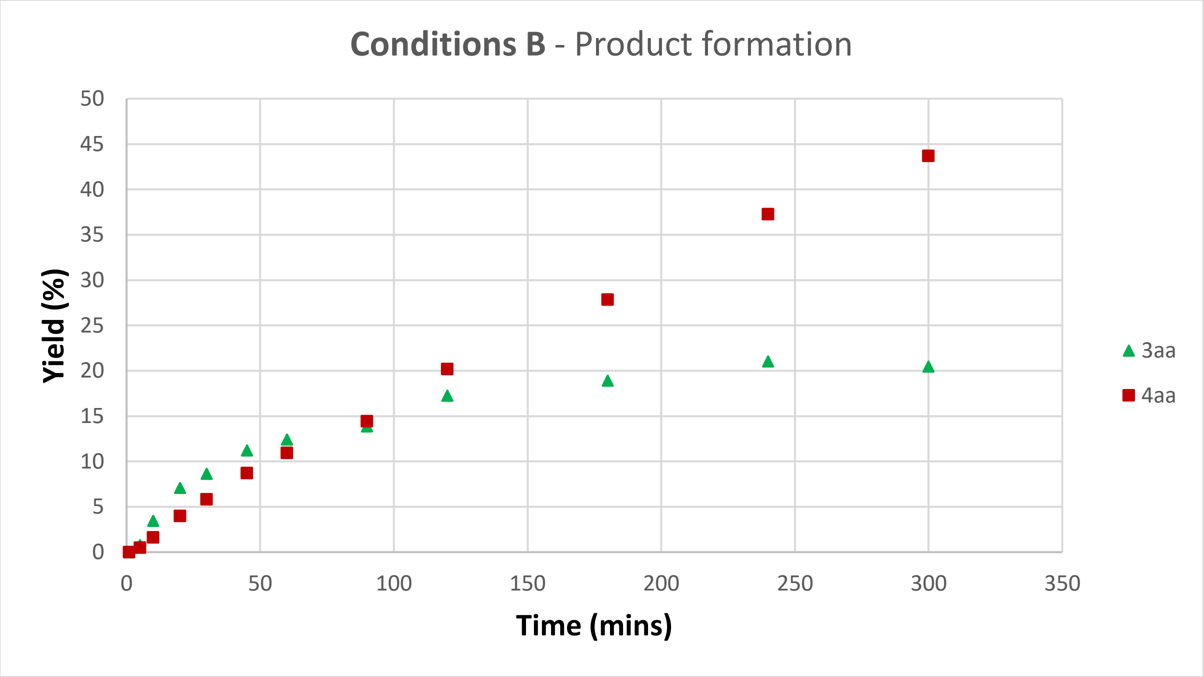

Figure S7. Graph of formation of **3aa** and **4aa** under conditions B.

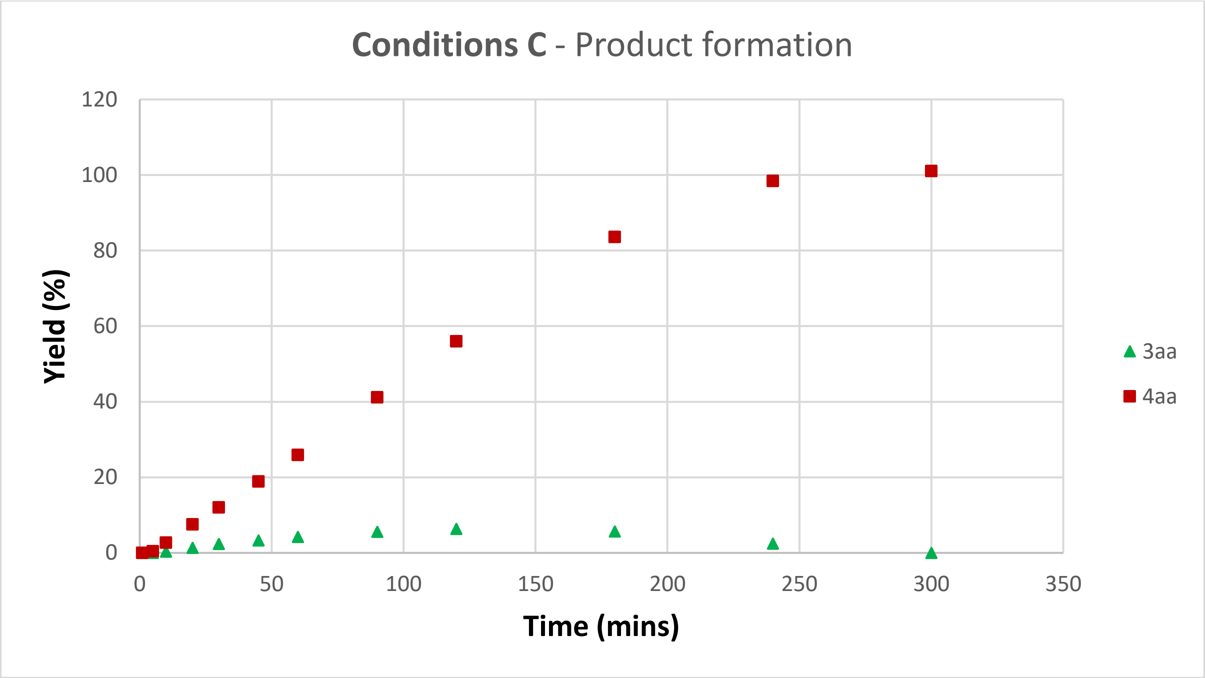

Figure S8. Graph of formation of **3aa** and **4aa** under conditions C.

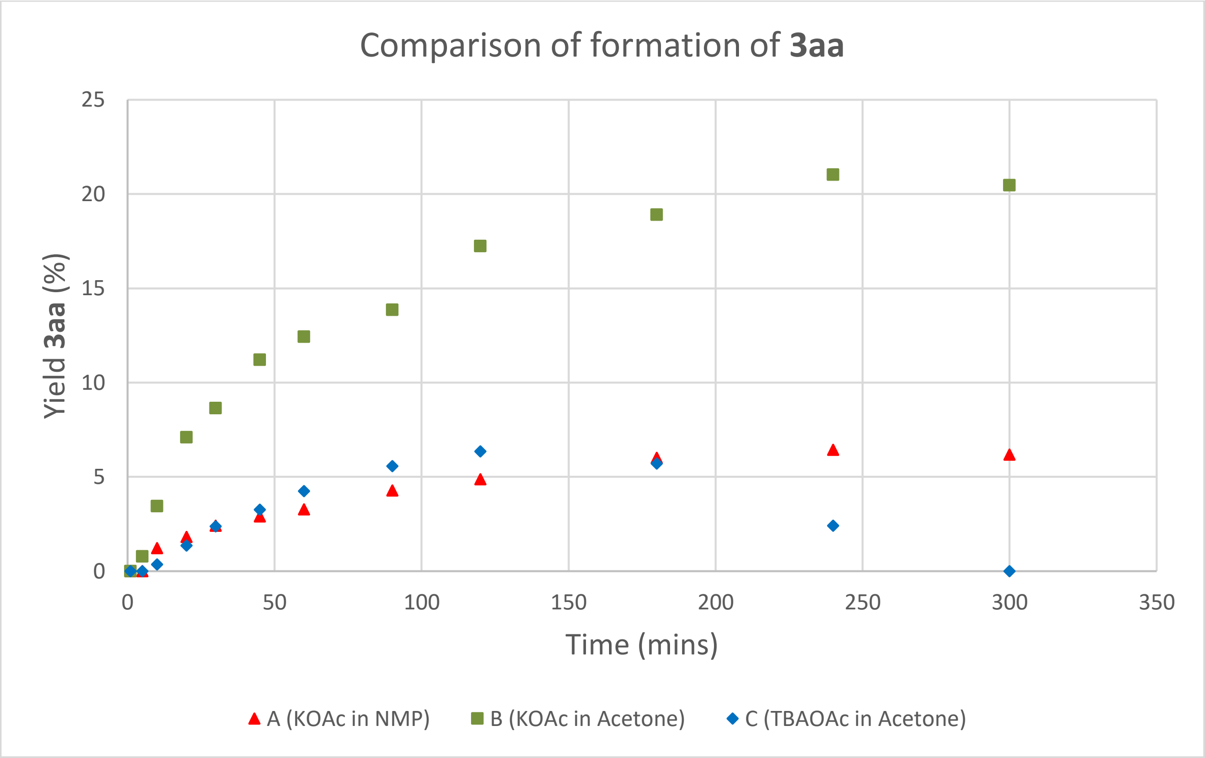

Figure S9. Graph of comparison of formation of **3aa** under conditions A, B and C.

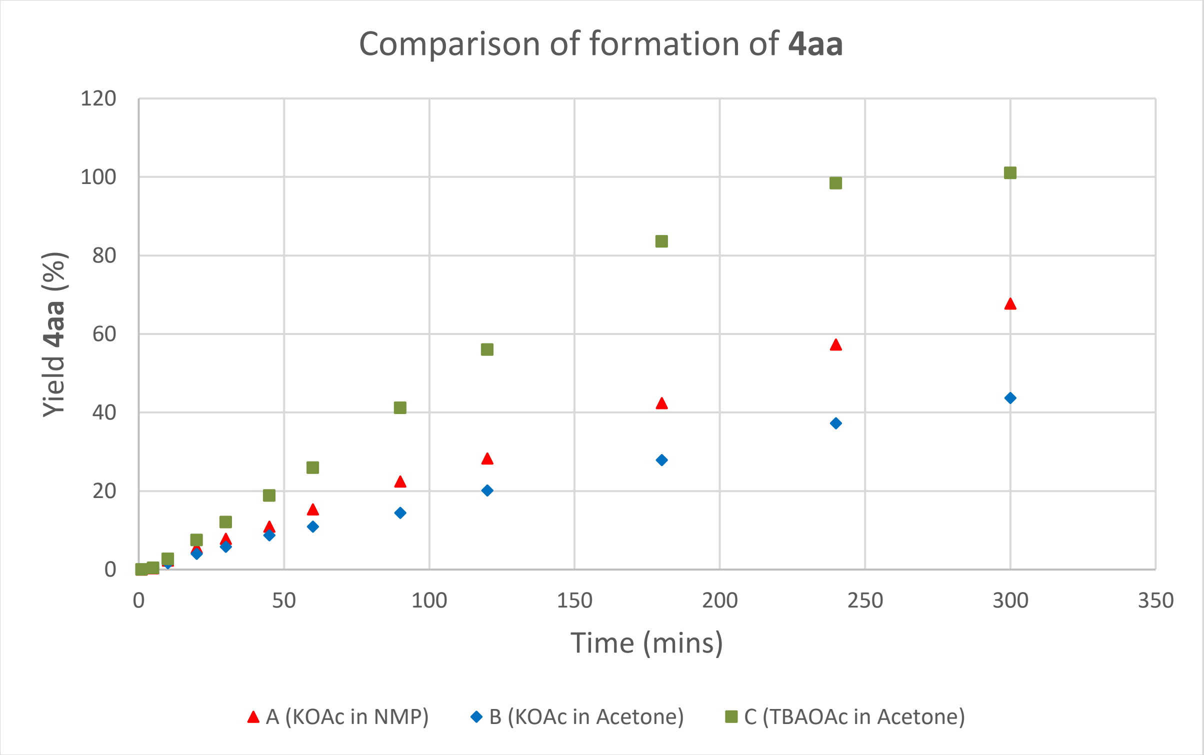

Figure S10. Graph of comparison of formation of **4aa** under conditions A, B and C.

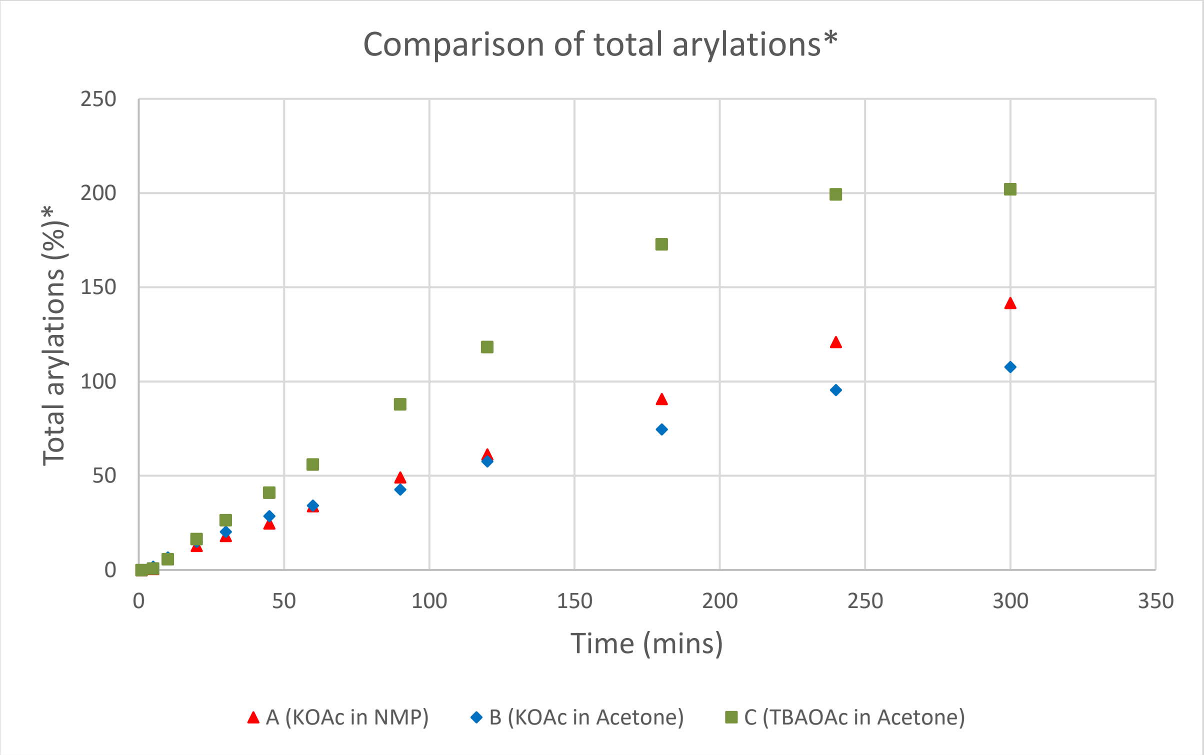

Figure S11. Graph of comparison of total arylations* under conditions A, B and C.

*Total arylations = (Yield **3aa**) + (2 x Yield **4aa**)

9. Improved Reaction conditions

For the following reactions, liquid reagents **1a** and **2a** were degassed using the freeze-pump-thaw method, with a minimum of three cycles, prior to being transferred into the glove box for use. The ruthenium catalyst (RuBnN) was prepared using the procedure described in section 2 and stored in the glove box for use. Potassium carbonate (K_2CO_3) and tetrabutylammonium acetate (TBAOAc) were ground using a pestle and mortar and dried in the vacuum oven for a minimum of 24 h, prior to being transferred into the glove box and stored there for use. Dry DMC solvent was purchased from Merck and degassed using the freeze-pump-thaw method, with a minimum of three cycles, prior to being transferred into the glove box for use.

Part 1. Low catalyst loading

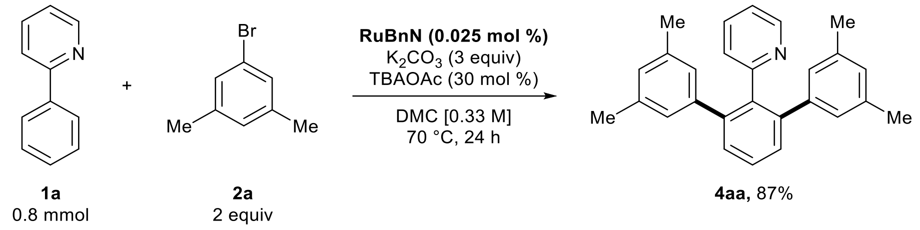

Scheme S4. Reaction conditions for low-catalyst loading reaction.

The reaction was set up in the glove box using a 10 mL crimp-cap microwave vial, to which was added: TBAOAc (72.4 mg, 0.24 mmol, 30 mol %), and K_2CO_3 (331.8 mg, 2.4 mmol, 3 equiv). The directing-group-containing arene 2-phenylpyridine **1a** (124.2 mg, 0.8 mmol, 1 equiv) and aryl halide 1-bromo-3,5-dimethylbenzene **2a** (296.2 mg, 1.60 mmol, 2 equiv) were then added by using an appropriate volume micro-syringe, followed by a stock solution of RuBnN in dimethyl carbonate (2.4 mL from a 8.33 x 10^{-4} M solution, 0.025 mol %). The vial was then crimp-capped and transferred outside the glove box to be stirred on a hotplate at 70 °C for 24 hours. Upon completion, the reaction was opened to air and filtered through a small silica pad, using Et_2O as the eluent. The crude reaction mixture was then loaded onto silica gel to be purified by flash column chromatography, to give product **4aa** as a white solid (252.8 mg, 87%).

Part 2. Large-scale reaction

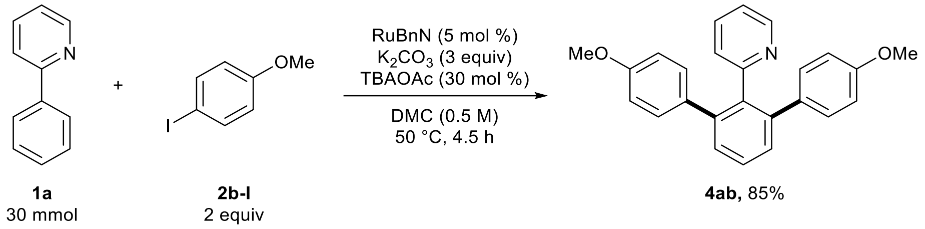

Scheme S5. Industrial large scale reaction conditions.

The following reaction was conducted within the research laboratory of AstraZeneca, Macclesfield, UK. RuBnN was prepared at the University of Manchester, shipped under an atmosphere of nitrogen and stored within a glovebox at AstraZeneca. All other reagents were purchased from the following supplier and used without purification. Tetrabutylammonium acetate TBAOAc (Fluorochem, ~90% containing AcOH, used without adjusting for purity), K_2CO_3 (Sigma-Aldrich, powdered), 2-phenylpyridine (Sigma-Aldrich), 4-iodo-anisole (Sigma-Aldrich) and dimethyl carbonate (Sigma-Aldrich). The reaction was conducted using an Easymax-402 (Mettler Toledo) fitted with a 100 mL reaction insert. A 1-piece glass reactor set fitted with a condenser, oxygen sensor, temperature probe, nitrogen inlet, charge port, and an overhead stirrer was used to ensure adequate exclusion of oxygen and mixing representative of a larger scale vessel **(Figure S12A)**. The reaction was performed at 0.5 M to ensure sufficient mixing of the heterogeneous mixture.

To the reaction vessel was added the solid reagents TBAOAc (2.71 g, 8.99 mmol, 0.300 equiv), K_2CO_3 (12.4 g, 90.0 mmol, 3.00 equiv), 4-iodoanisole **2b-I** (14.0 g, 60.0 mmol, 2.00 equiv). The reactor was flushed with N_2 to <0.05% with a high N_2 flow, before reducing the N_2 flow and partially submerging the outlet tube in water to create a positive pressure within the reactor. Dimethyl carbonate was degassed via N_2 bubbling and (45 mL, 1.5 L/mol) was added via syringe, then the stirrer set to 400 rpm to ensure good mixing of the heterogenous pale yellow mixture. 2-Phenylpyridine **1a** (4.66 g, 30.0 mmol, 1.00 equiv) was weighed into a glass vial, degassed via N_2 bubbling and maintained under N_2 atmosphere before addition via syringe to the reactor. The vial was washed with degassed dimethyl carbonate (5.0 mL, 0.17 L/mol) that was added via syringe to the reactor. A slurry of RuBnN (0.817 g, 1.50 mmol, 0.05 equiv) in degassed dimethyl carbonate (10 mL, 0.33 L/mol) was prepared in a glovebox then transferred to the reactor via a wide bore needle. The jacket temperature was set to 50 °C providing a steady internal reaction temperature of 49-50 °C during the course of the reaction. Within 5 minutes the reaction had turned dark red and remained dark red during the course of the reaction. After 4.5 h it was determined by UHPLC/MS that the reaction was complete (90% a/a 2-(4,4''-dimethoxy-[1,1':3',1''-terphenyl]-2'-yl)pyridine at 220 nm), the jacket temperature was set to 25 °C

and the reaction held overnight under N_2 atmosphere. The dark red/brown heterogenous mixture (**Figure S12B**) was diluted with MTBE (20 mL) and filtered through a ~150 mm diameter filter containing ~50 mm of silica. The reaction vessel was washed out with MTBE (3 x 100 mL) through the filter then the filter flushed with EtOAc (500 mL) to give a brown solution (**Figure S12C**). The solution was concentrated under reduced pressure to give a light brown solid 2-(4,4''-dimethoxy-[1,1':3',1''-terphenyl]-2'-yl)pyridine **4ab** (6.66 g, 90% purity, 16.3 mmol, 54% yield). The filter was further flushed with EtOAc (500 mL) and the filtrate concentrated under reduced pressure to give an off white solid 2-(4,4''-dimethoxy-[1,1':3',1''-terphenyl]-2'-yl)pyridine **4ab** (3.56 g, 95% purity, 9.21 mmol, 31% yield). Total combined material 25.5 mmol, 85% yield. Purity calculated via ^{1}H NMR vs 1,2,4,5-Tetrachloro-3-nitrobenzene and is the average of two samples. ^{1}H NMR in accordance with that previously reported (**Figure S13** and **S14**).

A

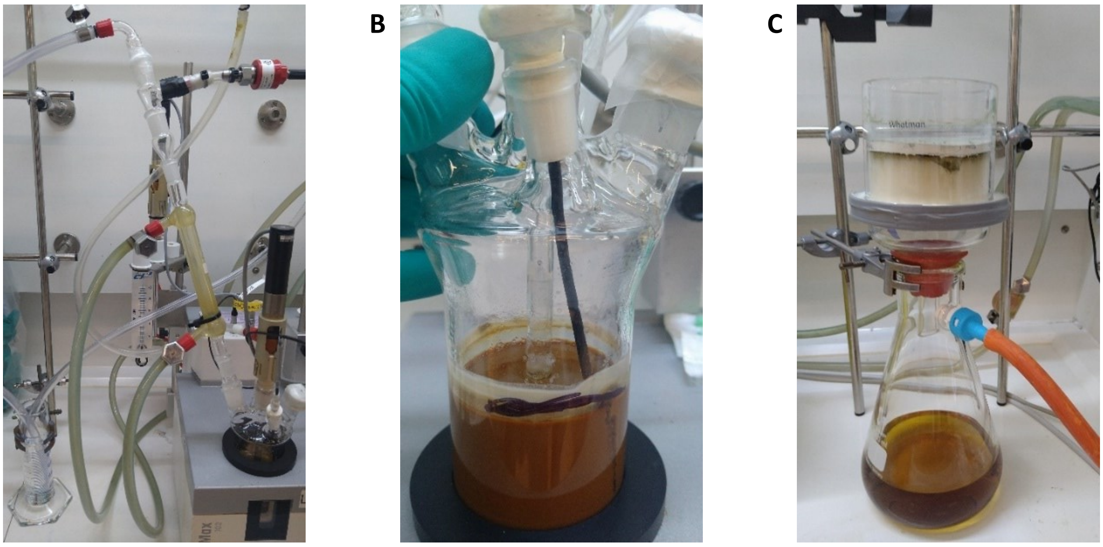

Figure S12. A. Reaction set up. B. Reaction mixture prior to filtration and C. Filter and collected filtrate.

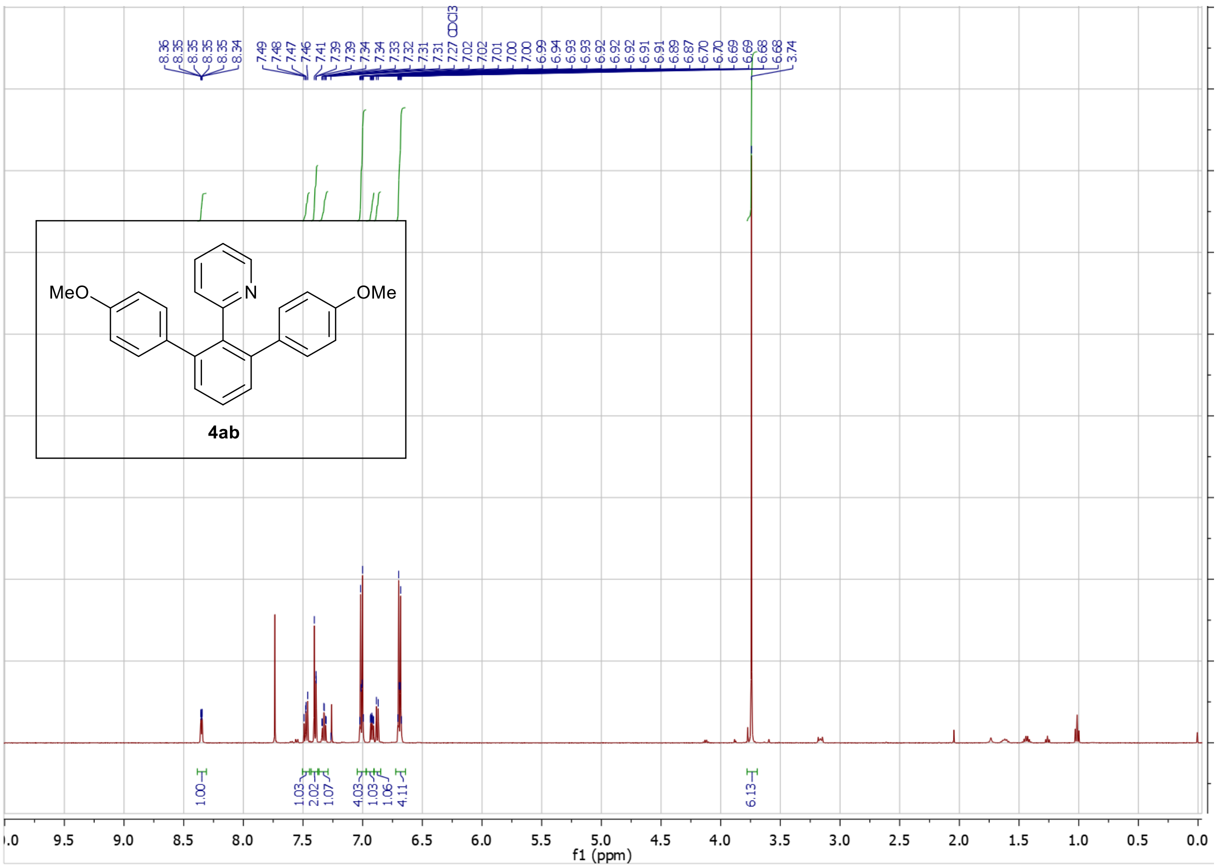

Average Purity = **90.08%**
Assuming sample weight: 33.92 mg, and mol weight: 367.44
Using Reference Compound: 2-3-5-6-TCNB (20.45 mg, 99.8% purity, Mol Weight=260.89)
Sample Integral 1: 8.31 - 8.37 ppm, value = 1.04 (1 nuclides) - Purity = 87.8%
Sample Integral 2: 7.44 - 7.5 ppm, value = 1.07 (1 nuclides) - Purity = 90.7%
Sample Integral 3: 7.29 - 7.35 ppm, value = 1.09 (1 nuclides) - Purity = 92.1%
Sample Integral 4: 6.97 - 7.04 ppm, value = 4.19 (4 nuclides) - Purity = 88.9%
Sample Integral 5: 6.89 - 6.95 ppm, value = 1.07 (1 nuclides) - Purity = 90.9%
Reference Integral: 7.71 - 7.75 ppm, value = 1 (1 nuclides)

Figure S13. ^{1}H NMR spectrum of batch 1 crude reaction product **4ab** for purity calculation ($CDCl_3$, 500 MHz).

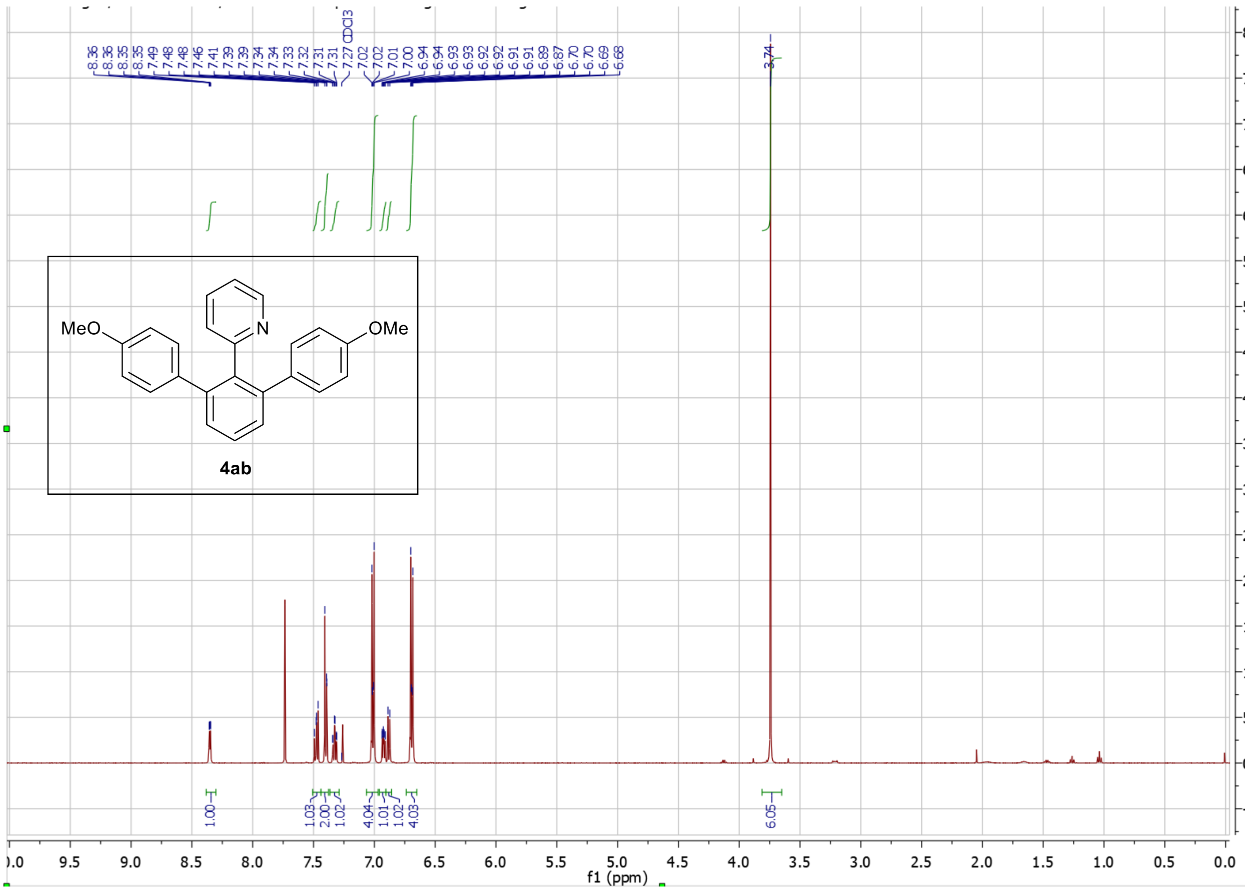

Average Purity = 94.64%
Assuming sample weight: 21.83 mg, and mol weight: 367.44
Using Reference Compound: 2-3-5-6-TCNB (20.22 mg, 99.8% purity, Mol Weight=260.89)
Sample Integral 1: 8.3 - 8.39 ppm, value = 0.72 (1 nuclides) - Purity = 93.8%
Sample Integral 2: 7.43 - 7.5 ppm, value = 0.74 (1 nuclides) - Purity = 95.8%
Sample Integral 3: 7.36 - 7.42 ppm, value = 1.44 (2 nuclides) - Purity = 93.6%
Sample Integral 4: 7.29 - 7.36 ppm, value = 0.74 (1 nuclides) - Purity = 95.8%
Sample Integral 5: 6.96 - 7.05 ppm, value = 2.89 (4 nuclides) - Purity = 94.2%
Reference Integral: 7.7 - 7.77 ppm, value = 1 (1 nuclides)

Figure S14. ^{1}H NMR spectrum of batch 2 crude reaction product **4ab** for purity calculation ($CDCl_3$, 500 MHz).

Part 3. Room temperature reactivity

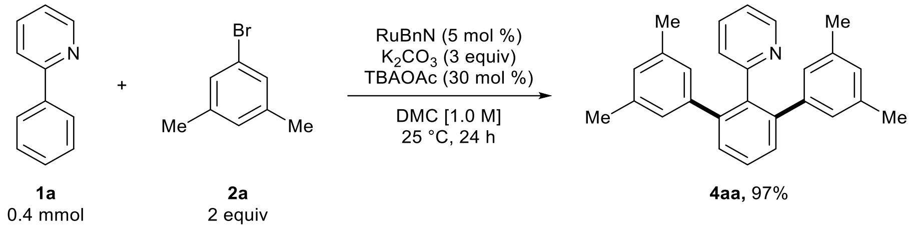

Scheme S6. Reaction conditions for room-temperature reaction.

The reaction was set up in the glove box using a 10 mL crimp-cap microwave vial, to which was added: RuBnN (10.8 mg, 0.02 mmol, 5 mol %), TBAOAc (36.2 mg, 0.12 mmol, 30 mol %), and K_2CO_3 (165.9 mg, 1.2 mmol, 3 equiv). The directing-group-containing arene 2-phenylpyridine **1a** (64.1 mg, 0.4 mmol, 1 equiv) and aryl halide 1-bromo-3,5-dimethylbenzene **2a** (148.1 mg, 0.8 mmol, 2 equiv) were then added by using an appropriate volume syringe, followed by dimethyl carbonate (0.4 mL). The vial was then crimp-capped and transferred outside the glove box to be stirred on a hotplate at 25 °C for 24 hours. Upon completion, the reaction was opened to air and filtered through a small silica pad, using Et_2O as the eluent. The crude reaction mixture was then loaded onto silica gel to be purified by flash column chromatography, to give product **4aa** as a white solid (140.6 mg, 97%).

Part 4. Fast reaction time

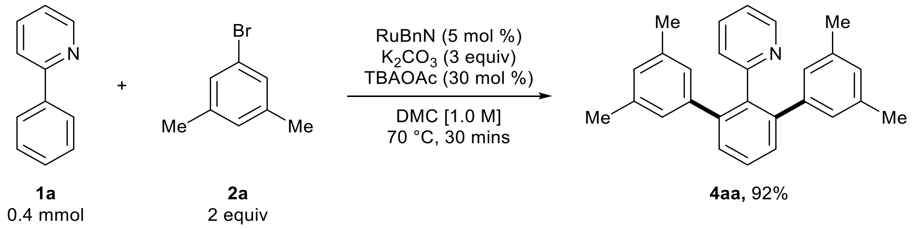

Scheme S7. Reaction conditions for fast reaction time.

The reaction was set up in the glove box using a 10 mL crimp-cap microwave vial, to which was added: RuBnN (10.8 mg, 0.02 mmol, 5 mol %), TBAOAc (36.2 mg, 0.12 mmol, 30 mol %), and K_2CO_3 (165.9 mg, 1.2 mmol, 3 equiv). The directing-group-containing arene 2-phenylpyridine **1a** (64.1 mg, 0.4 mmol, 1 equiv) and aryl halide 1-bromo-3,5-dimethylbenzene **2a** (148.1 mg, 0.8 mmol, 2 equiv) were then added by using an appropriate volume syringe, followed by dimethyl carbonate (0.4 mL). The vial was then crimp-capped and transferred outside the glove box to be stirred on a hotplate at 70 °C for 30 mins. Upon completion, the reaction was opened to air and filtered through a small silica pad, using Et_2O as the eluent. The crude reaction mixture was then loaded onto silica gel to be purified by flash column chromatography, to give product **4aa** as a white solid (134.4 mg, 92%).

10. Copies of NMR spectra

Figure S15. ^{1}H NMR ($CDCl_3$, 500 MHz) of **4aa**.

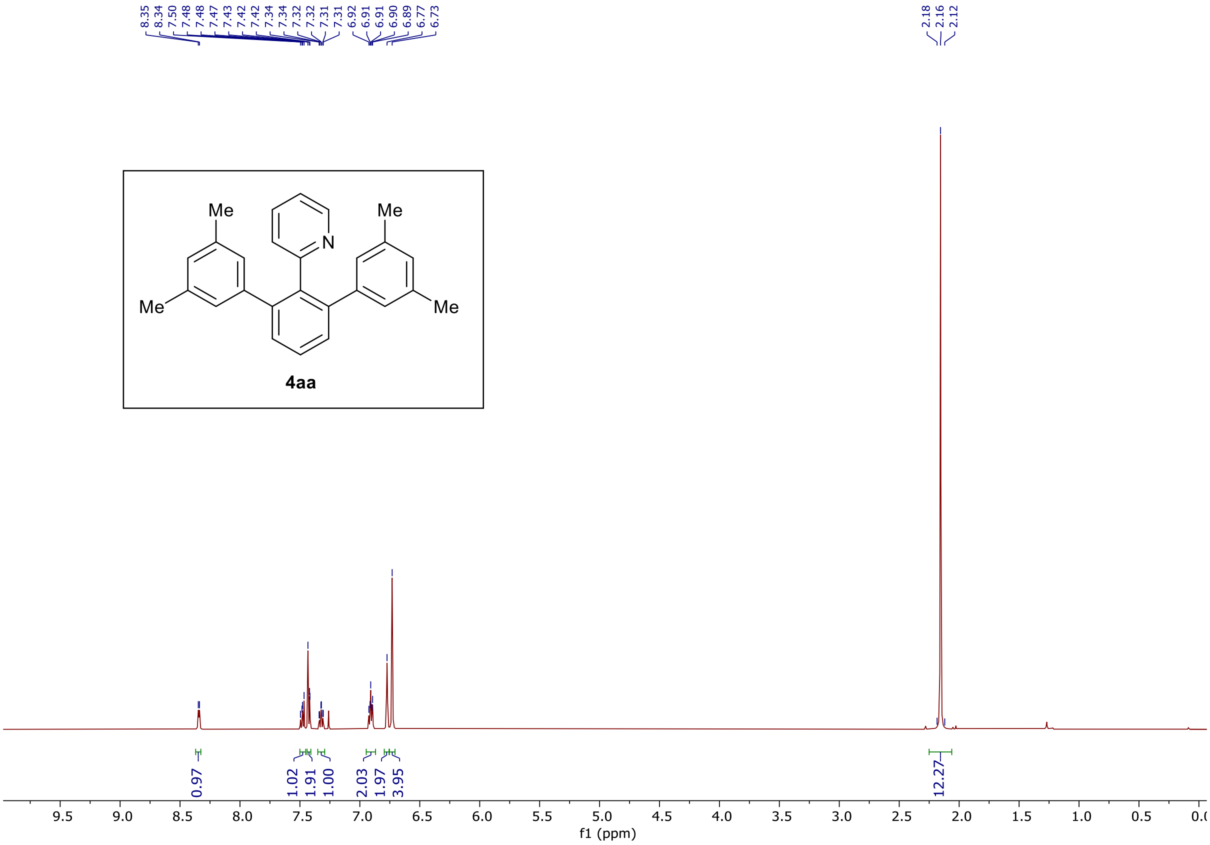

Figure S16. ^{13}C NMR ($CDCl_3$, 126 MHz) of **4aa**.

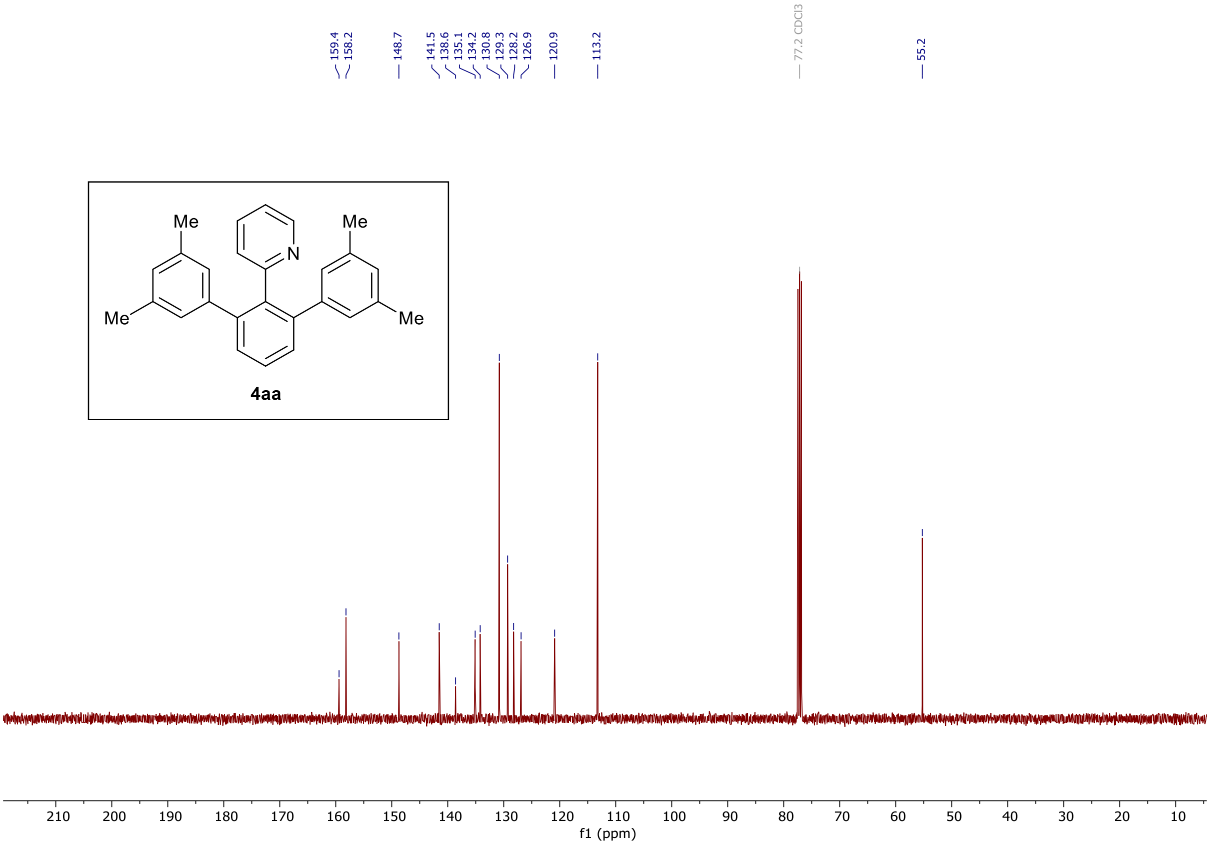

Figure S17. ^{1}H NMR ($CDCl_3$, 400 MHz) of **4ba**.

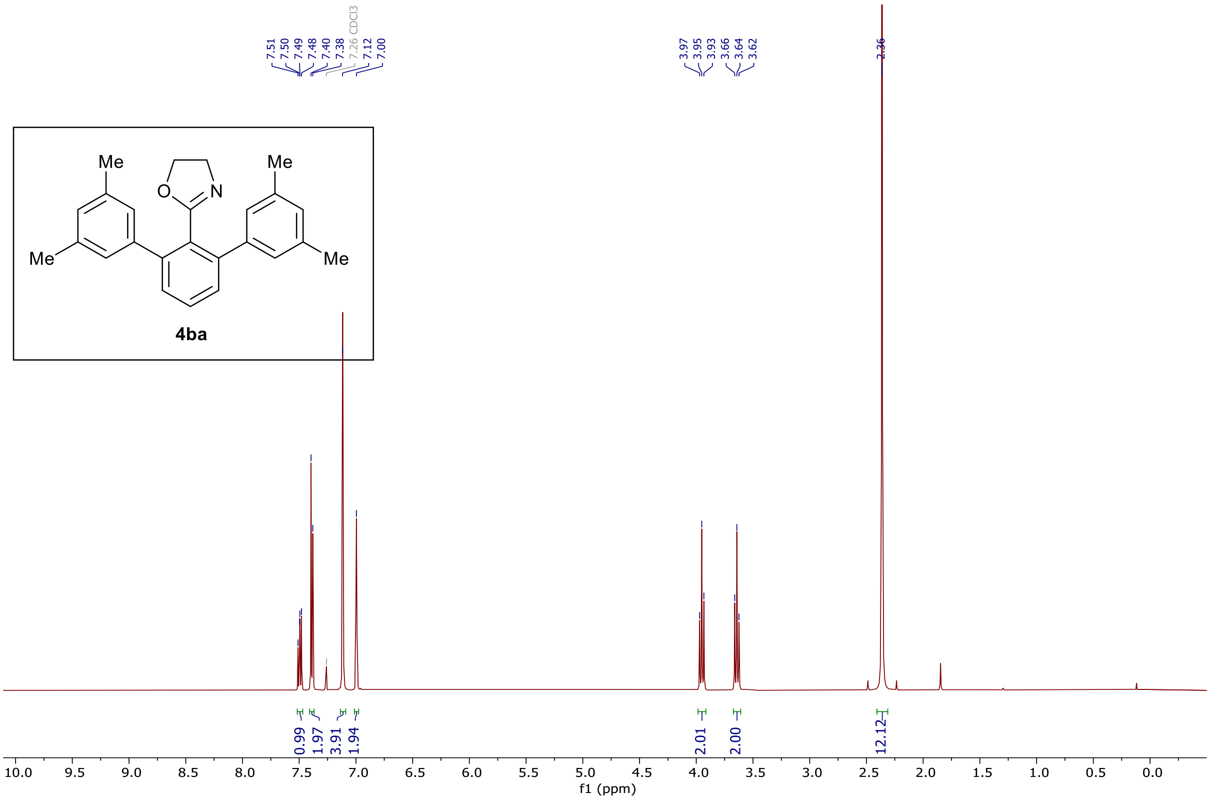

Figure S18. ^{13}C NMR ($CDCl_3$, 101 MHz) of **4ba**.

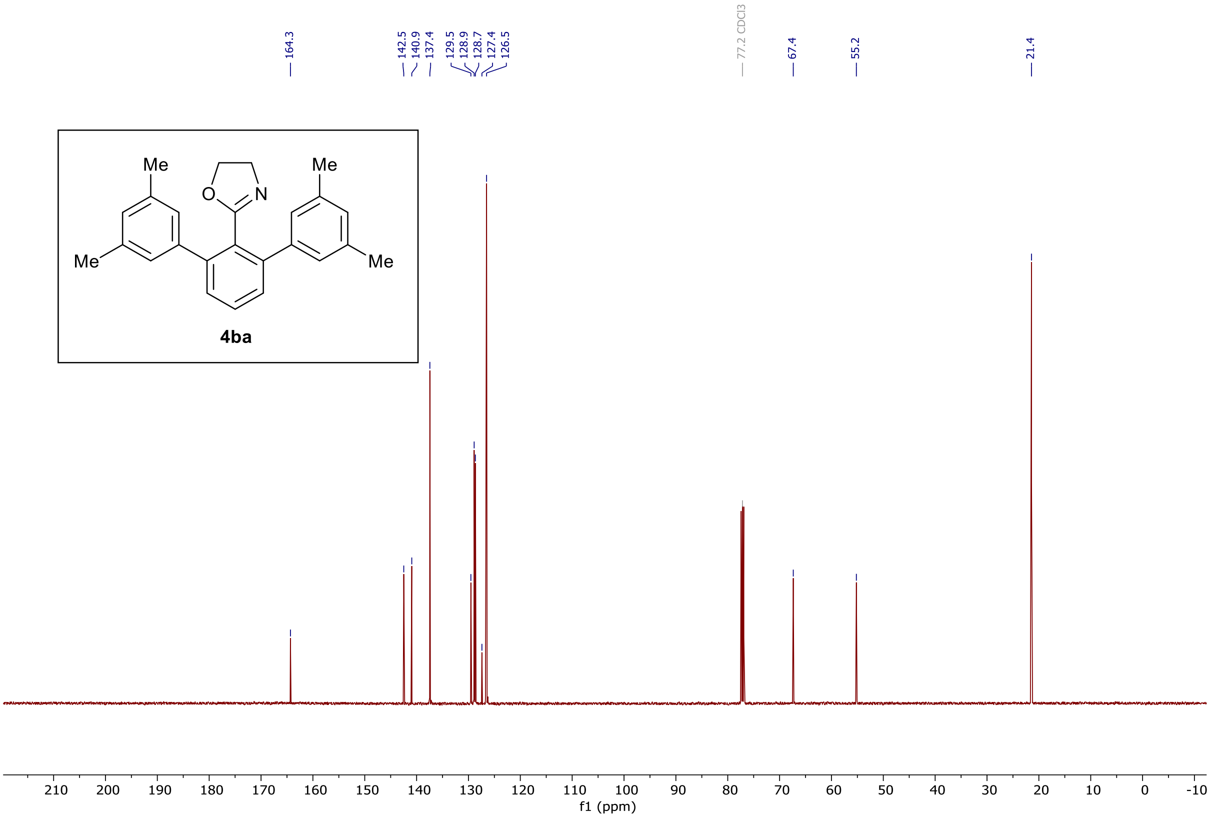

Figure S19. ^{1}H NMR ($CDCl_3$, 400 MHz) of **3ca**.

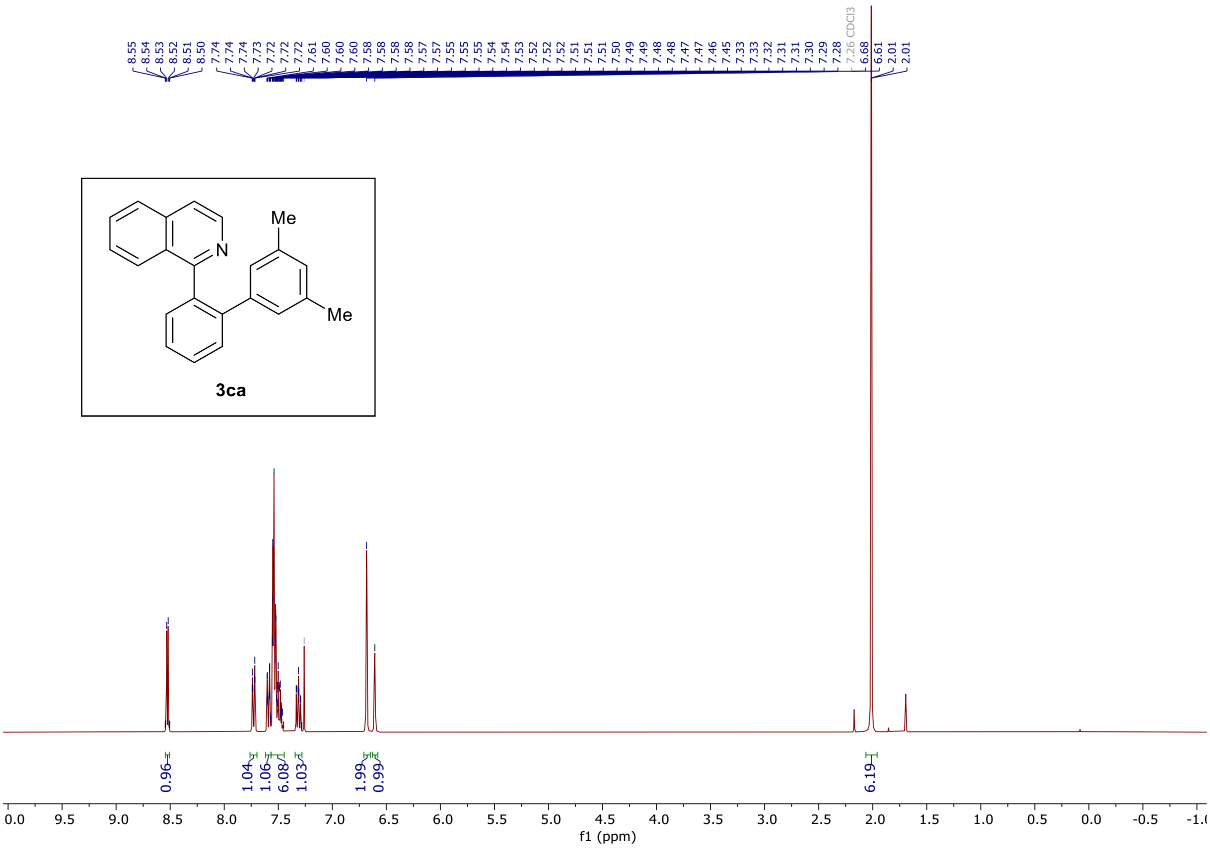

Figure S20. ^{13}C NMR ($CDCl_3$, 101 MHz) of **3ca.**

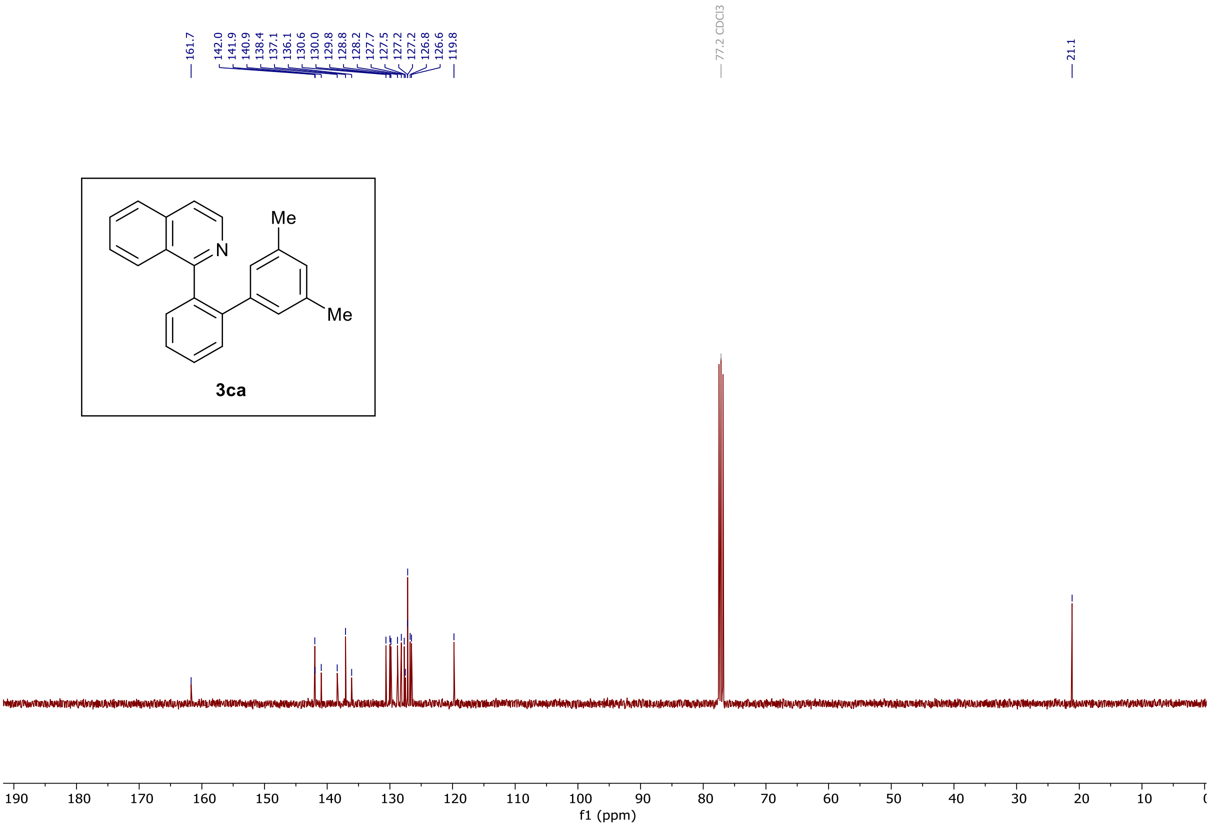

Figure S21. ^{1}H NMR ($CDCl_3$, 400 MHz) of **4da**.

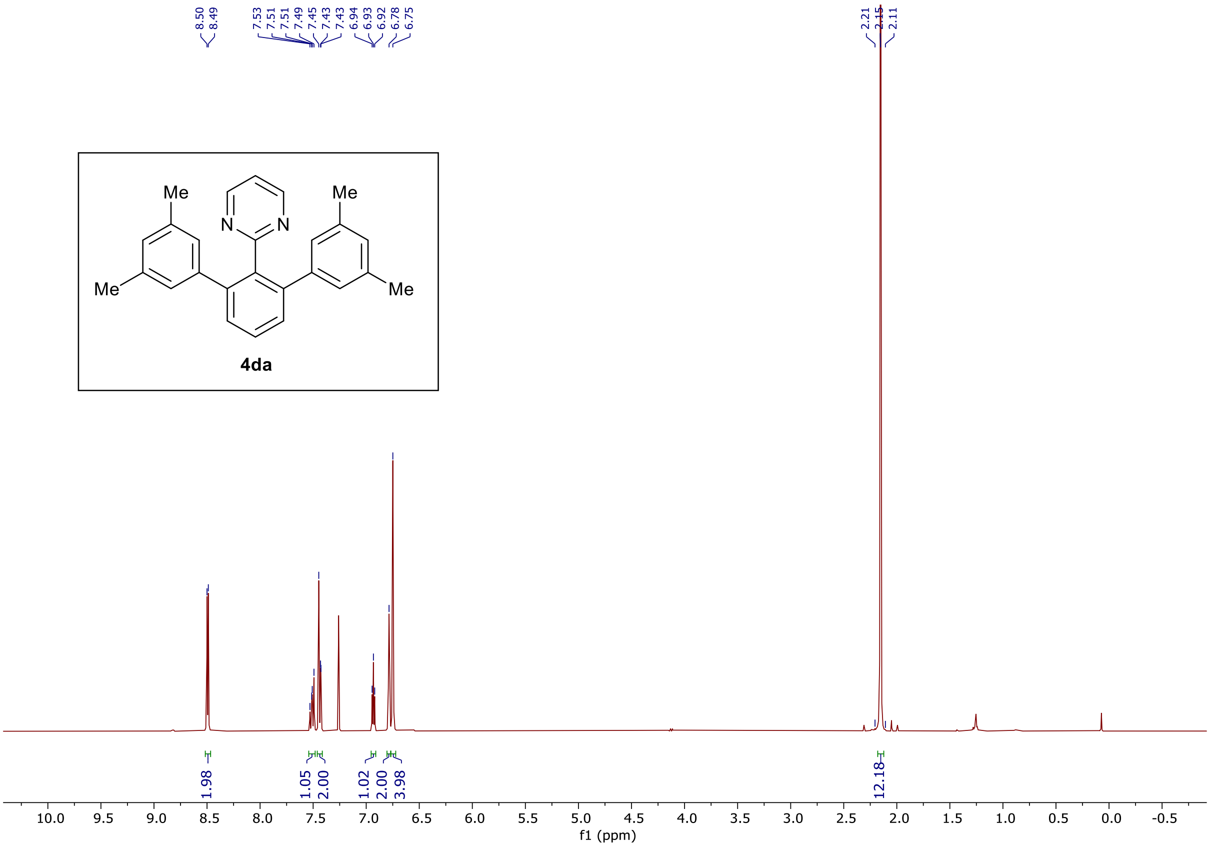

Figure S22. ^{13}C NMR ($CDCl_3$, 126 MHz) of **4da**.

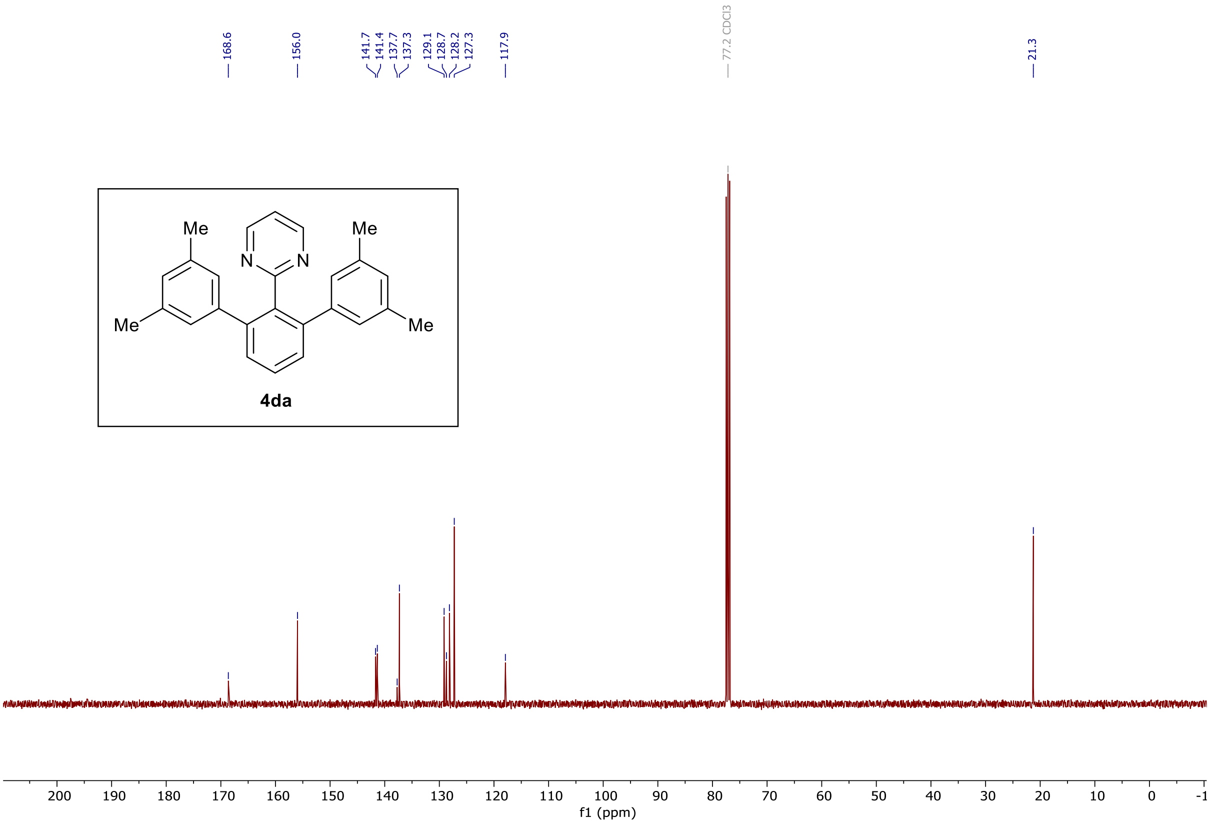

Figure S23. ^{1}H NMR ($CDCl_3$, 500 MHz) of **4ea**.

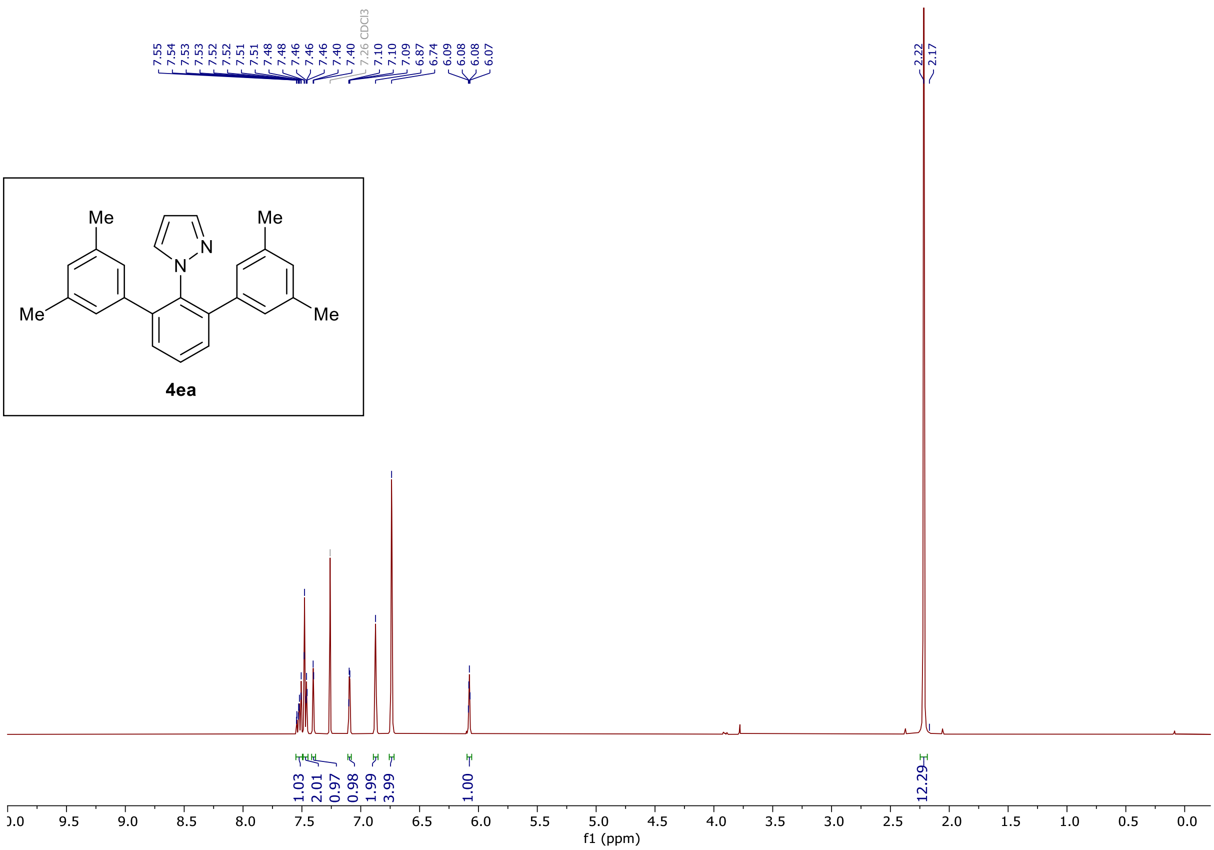

Figure S24. ^{13}C NMR ($CDCl_3$, 126 MHz) of **4ea**.

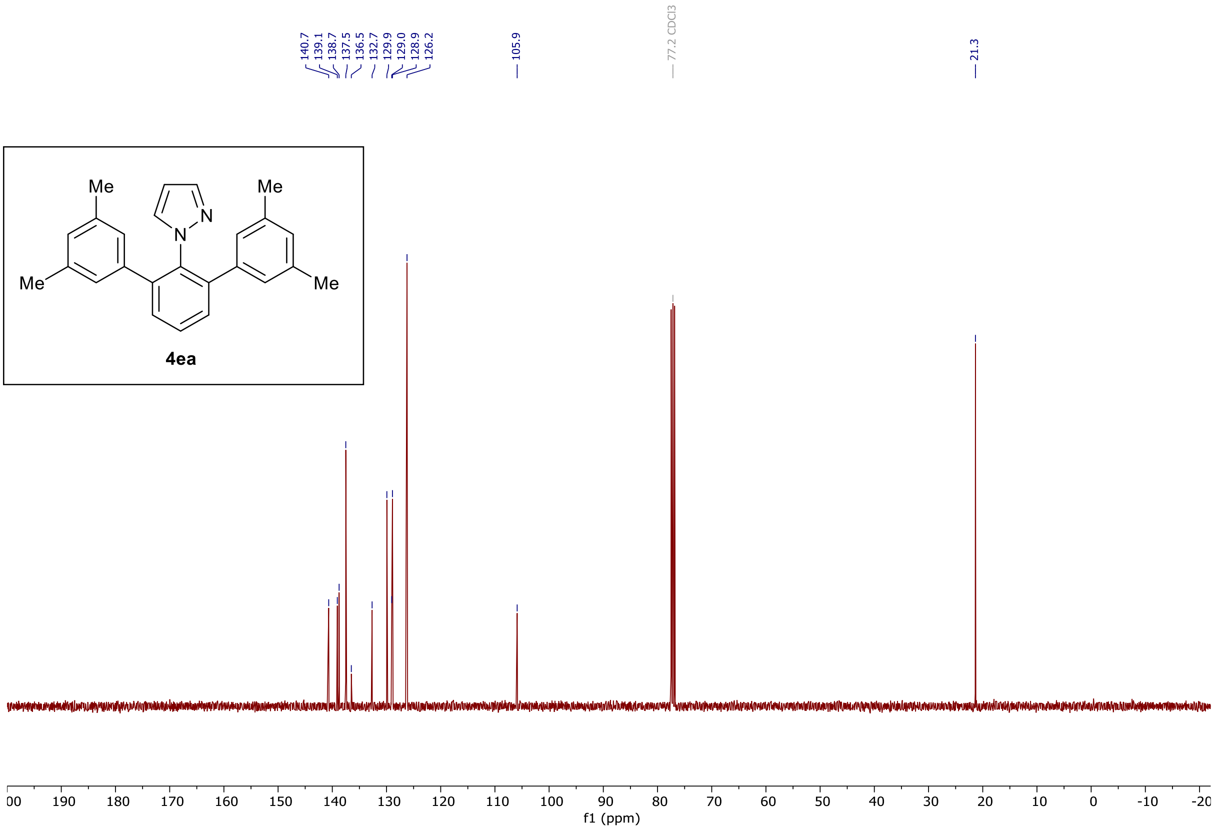

Figure S25. ^{1}H NMR ($CDCl_3$, 400 MHz) of **4fa**.

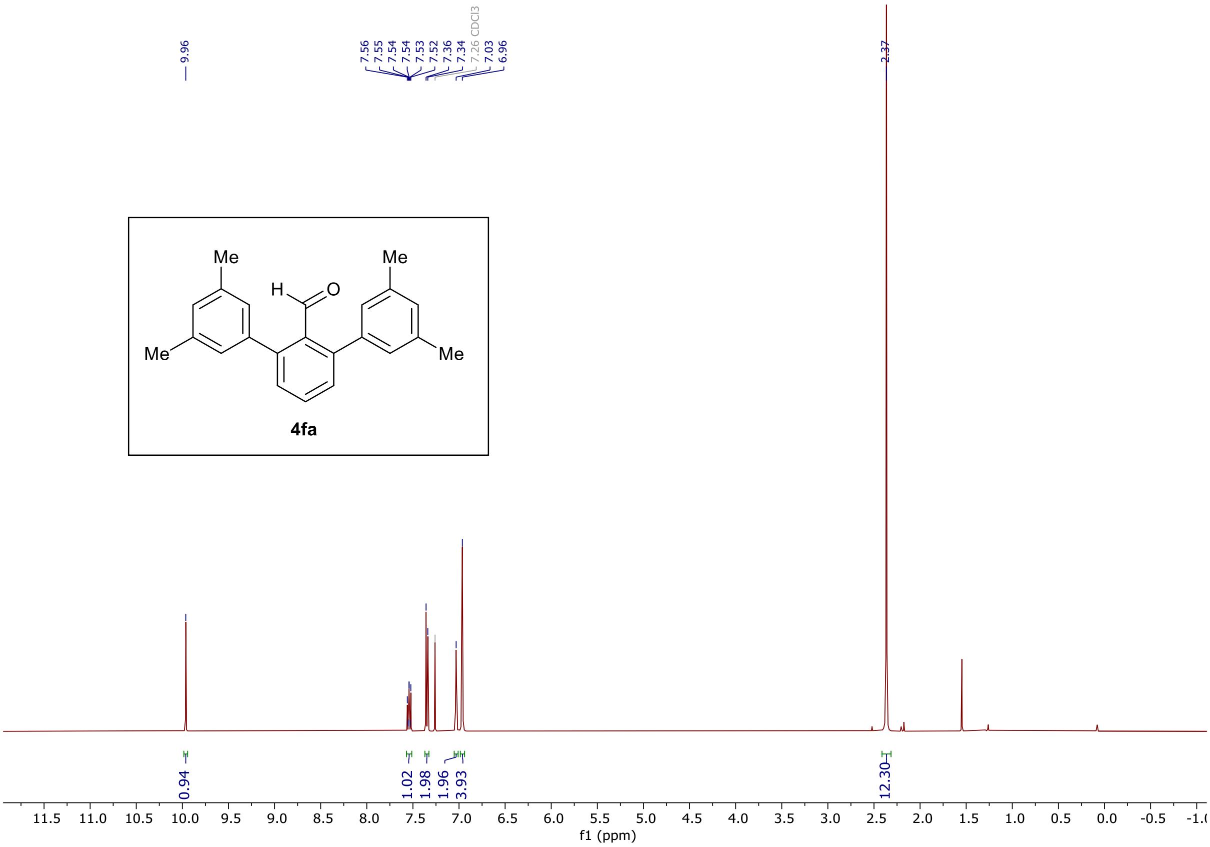

Figure S26. ^{13}C NMR ($CDCl_3$, 101 MHz) of **4fa**.

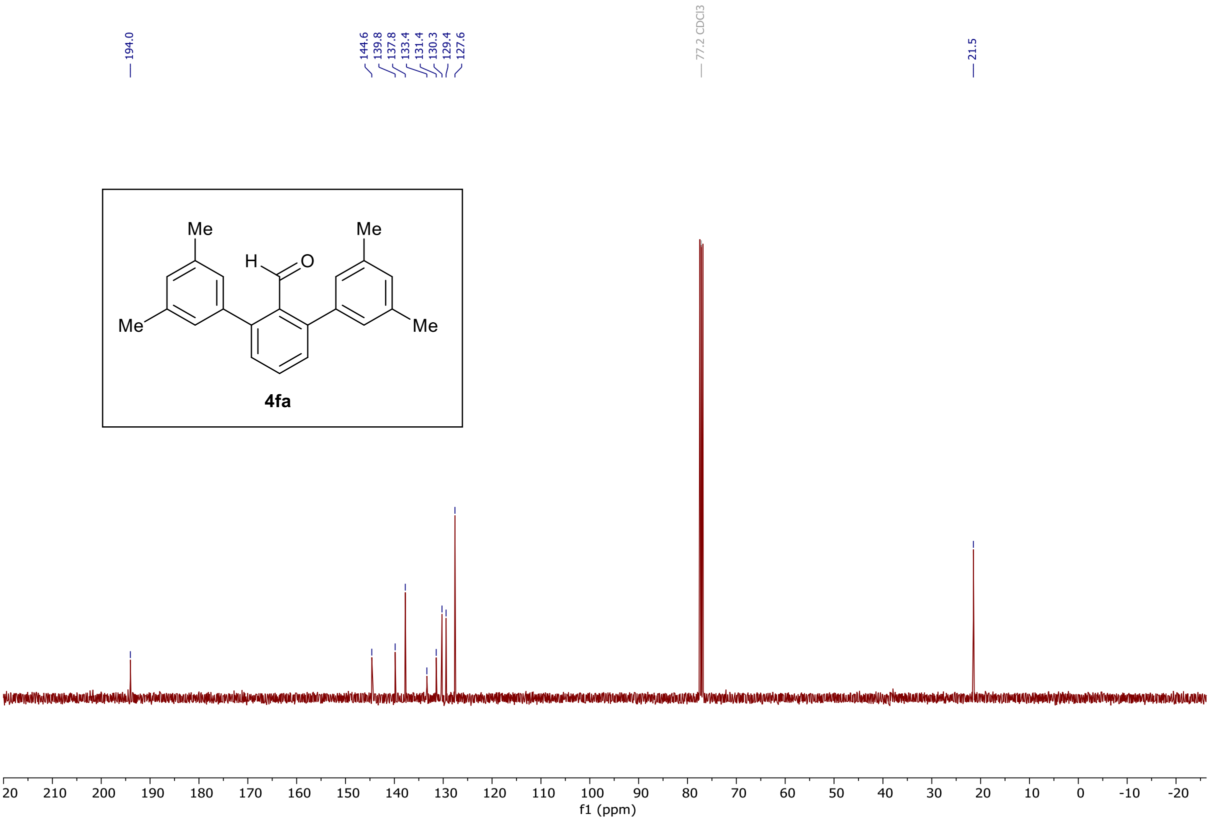

Figure S27. ^{1}H NMR ($CDCl_3$, 400 MHz) of **4ab**.

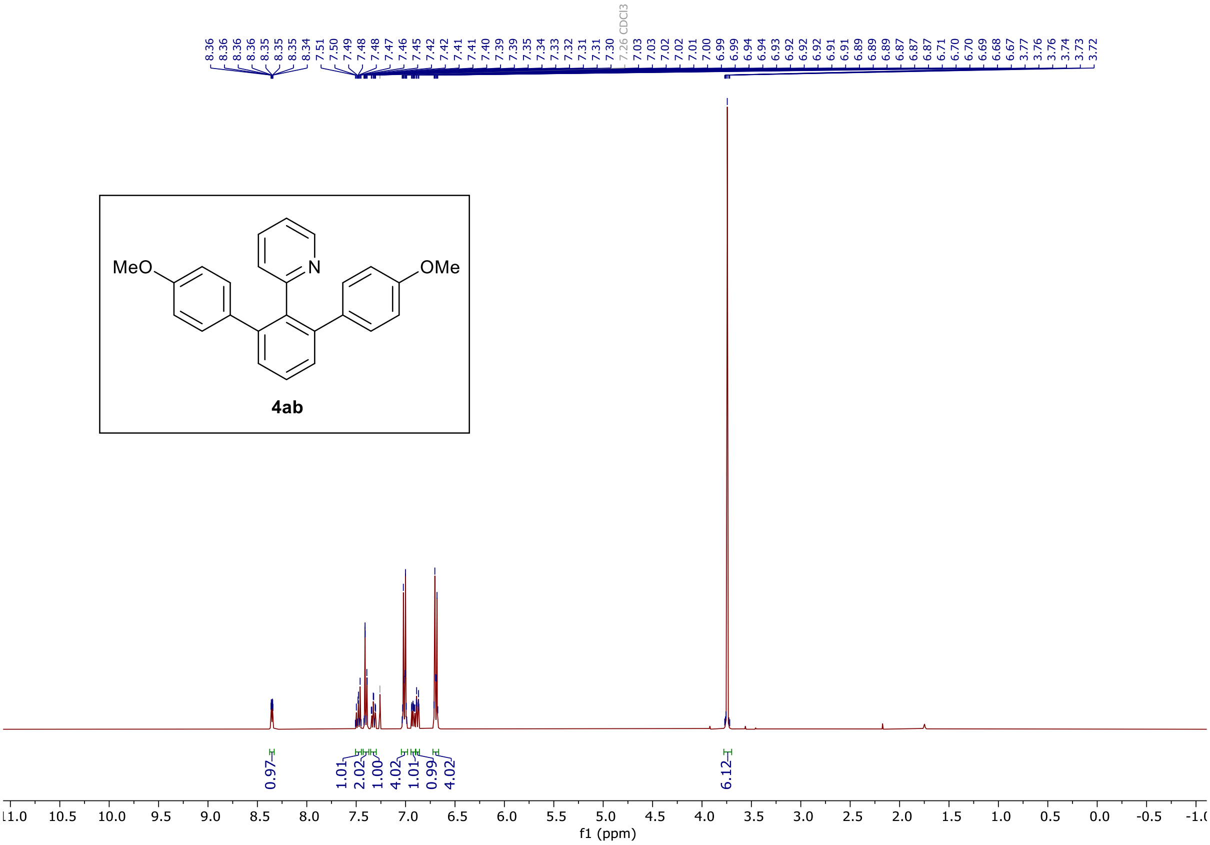

Figure S28. ^{13}C NMR ($CDCl_3$, 101 MHz) of **4ab**.

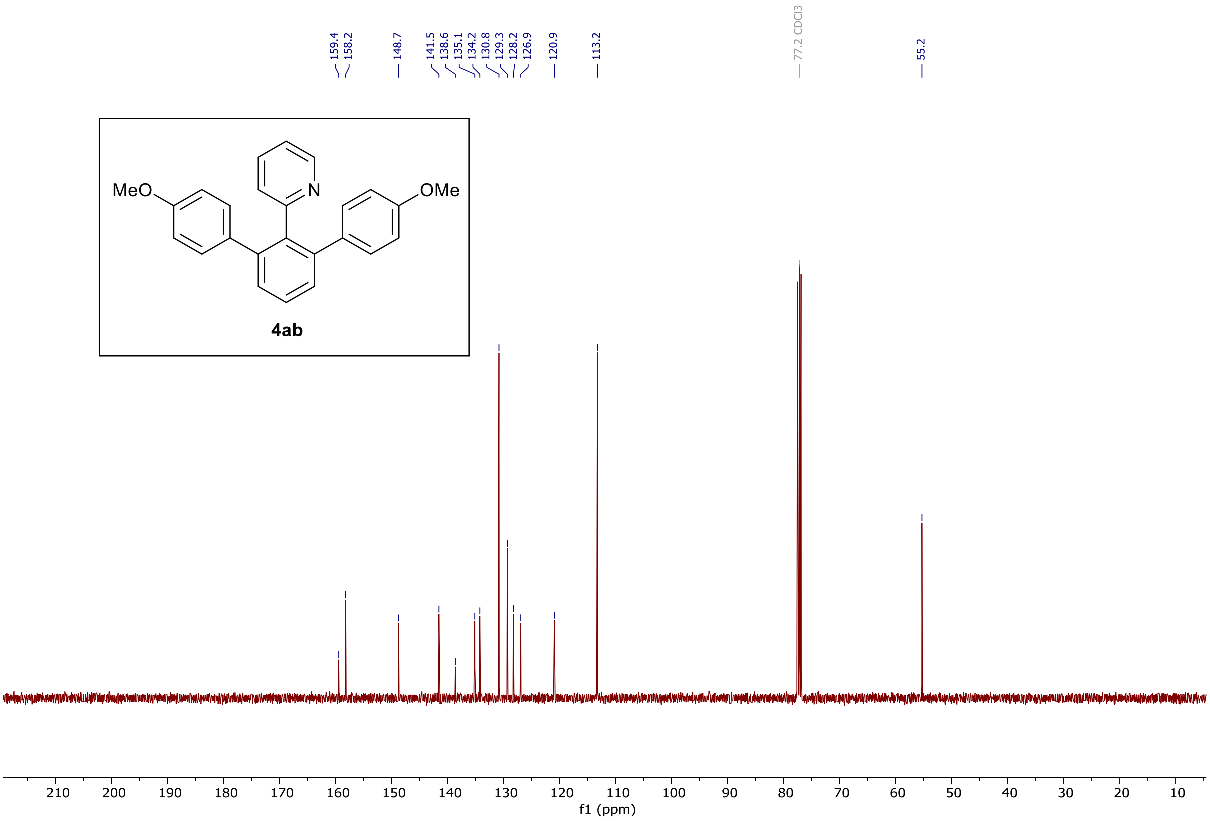

Figure S29. ^{1}H NMR ($CDCl_3$, 500 MHz) of **4ac**.

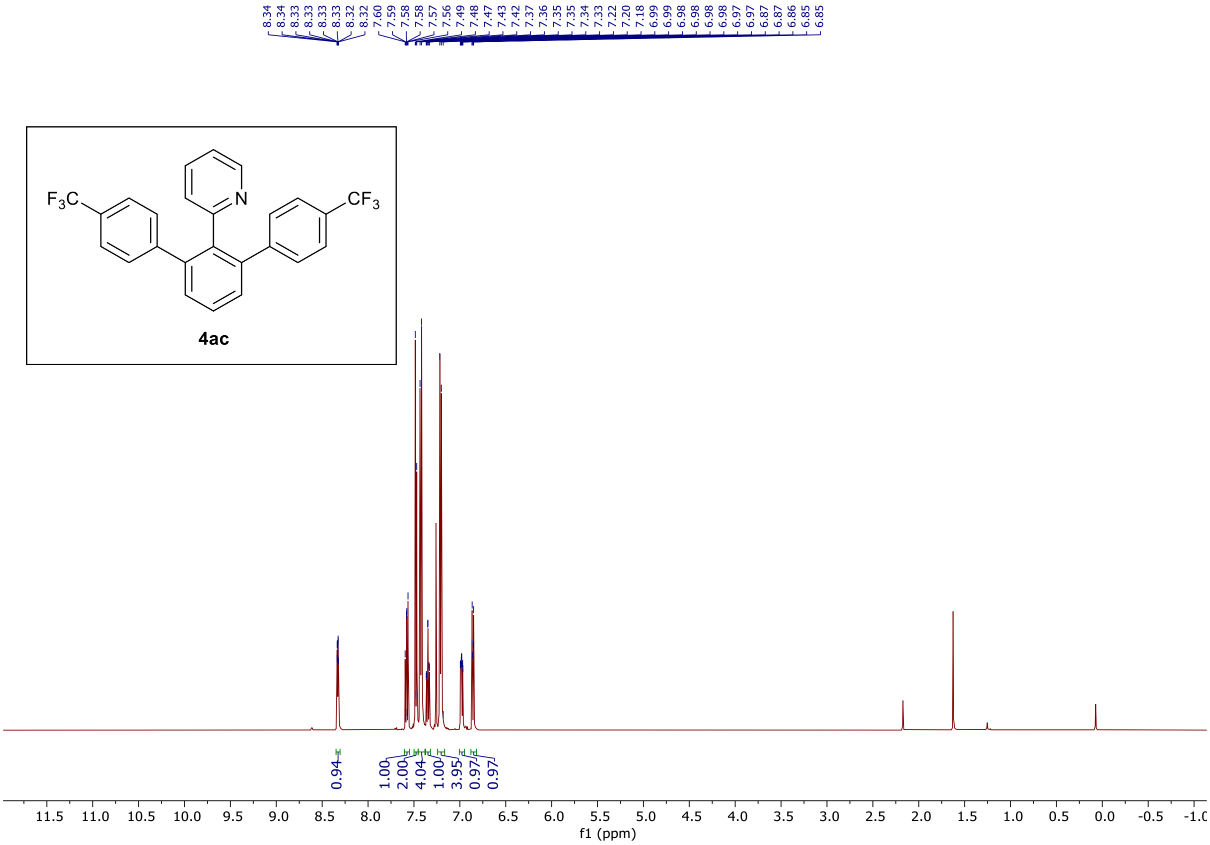

Figure S30. ^{13}C NMR ($CDCl_3$, 126 MHz) of **4ac**.

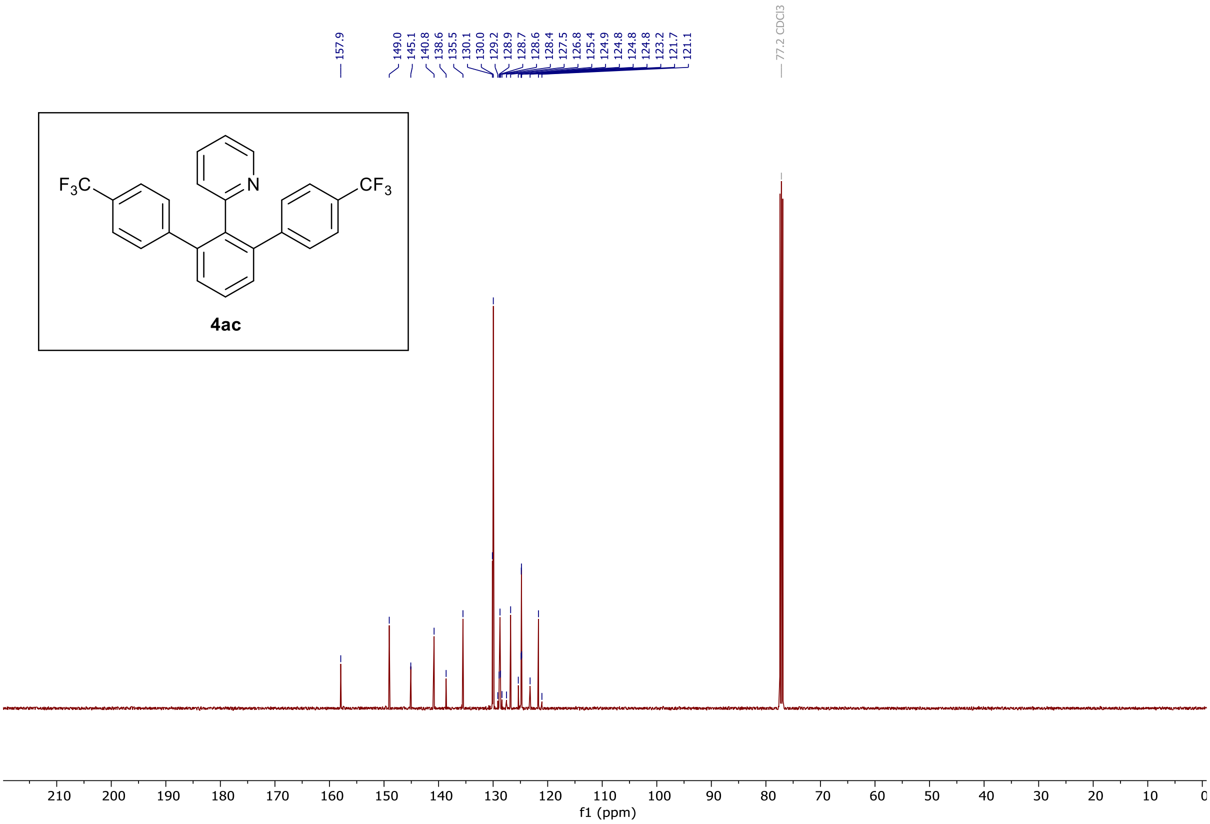

Figure S31. ^{19}F NMR ($CDCl_3$, 471 MHz) of **4ac**.

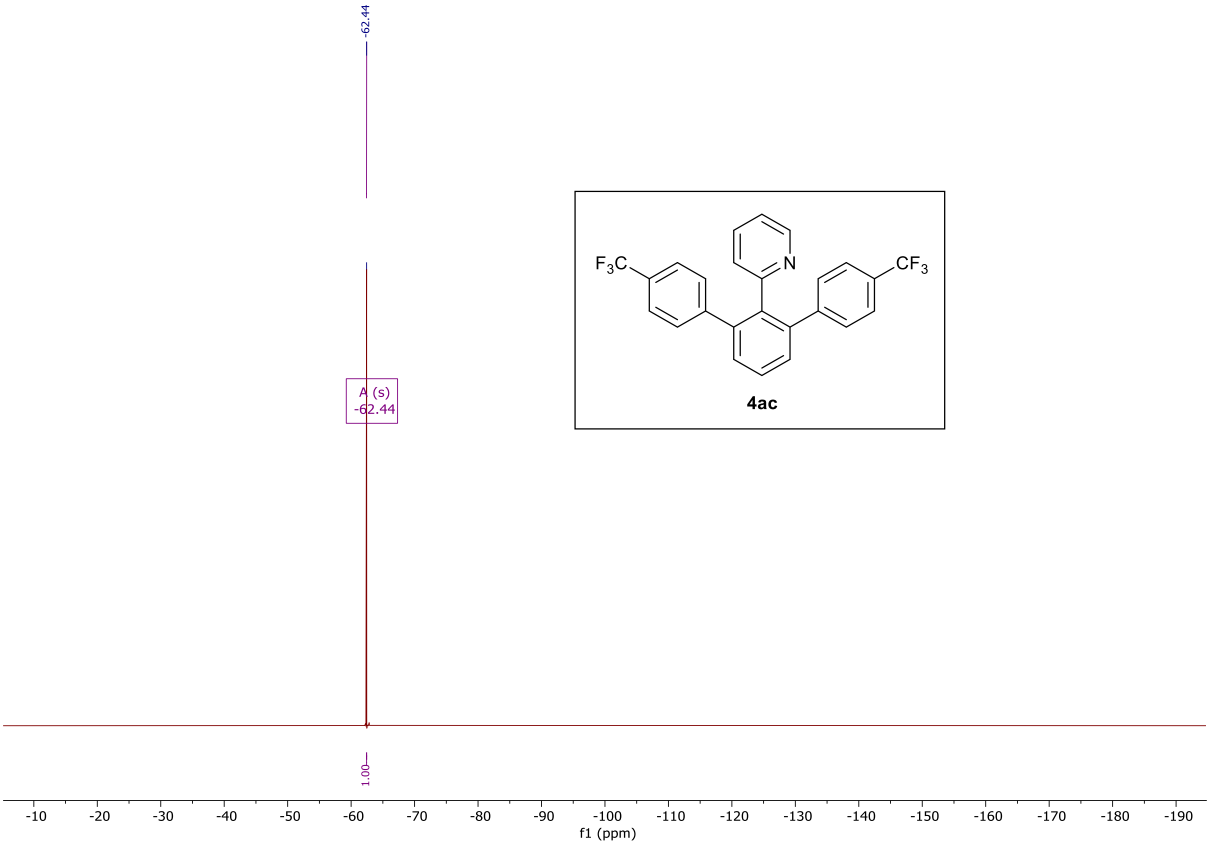

Figure S32. ^{1}H NMR ($CDCl_3$, 500 MHz) of **3gb**.

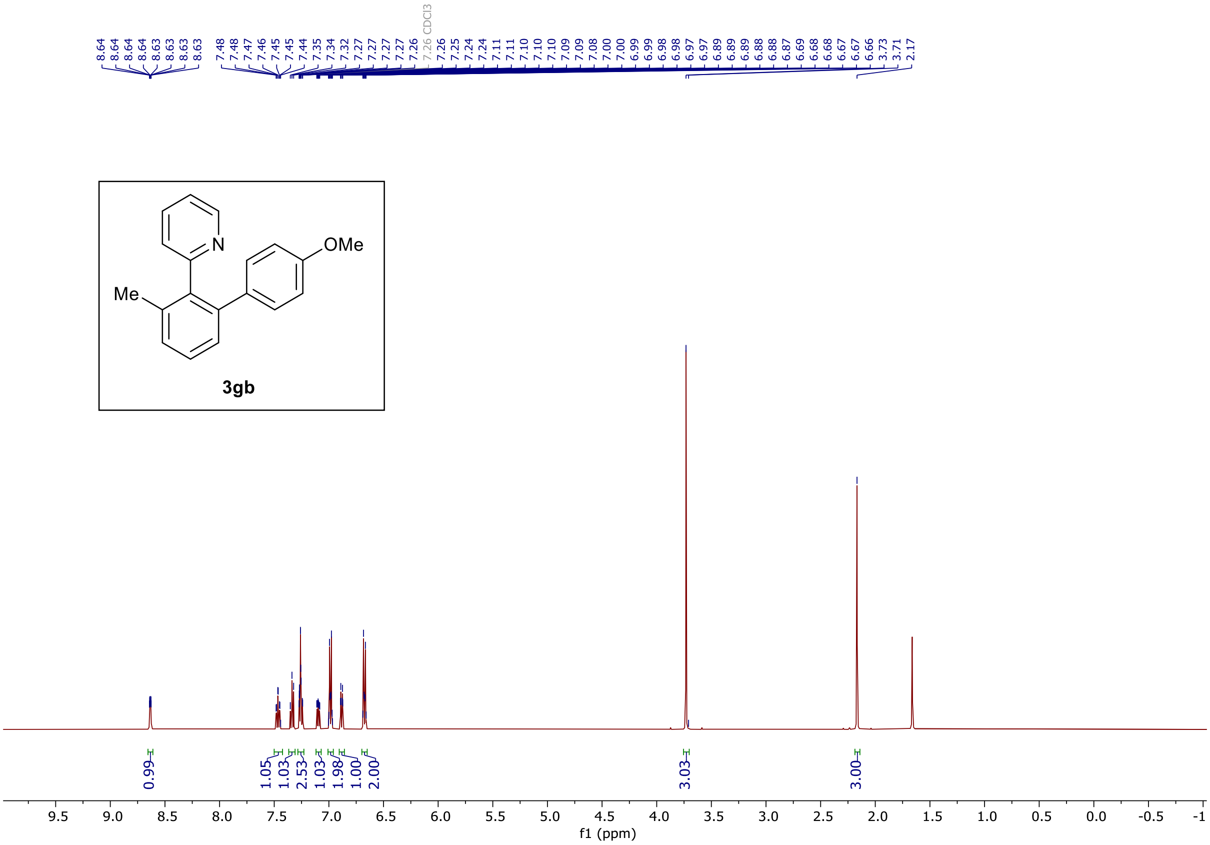

Figure S33. ^{13}C NMR ($CDCl_3$, 126 MHz) of **3gb**.

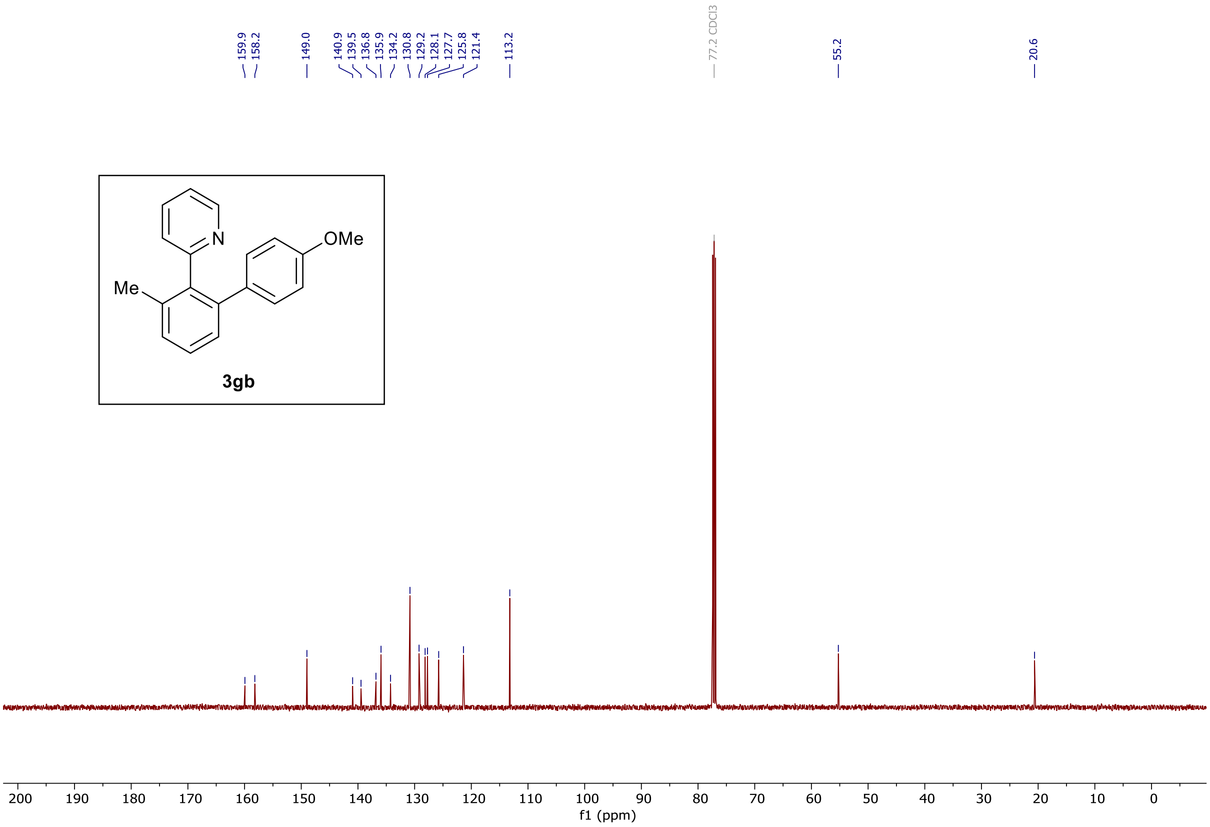

Figure S34. ^{1}H NMR ($CDCl_3$, 500 MHz) of **3gc**.

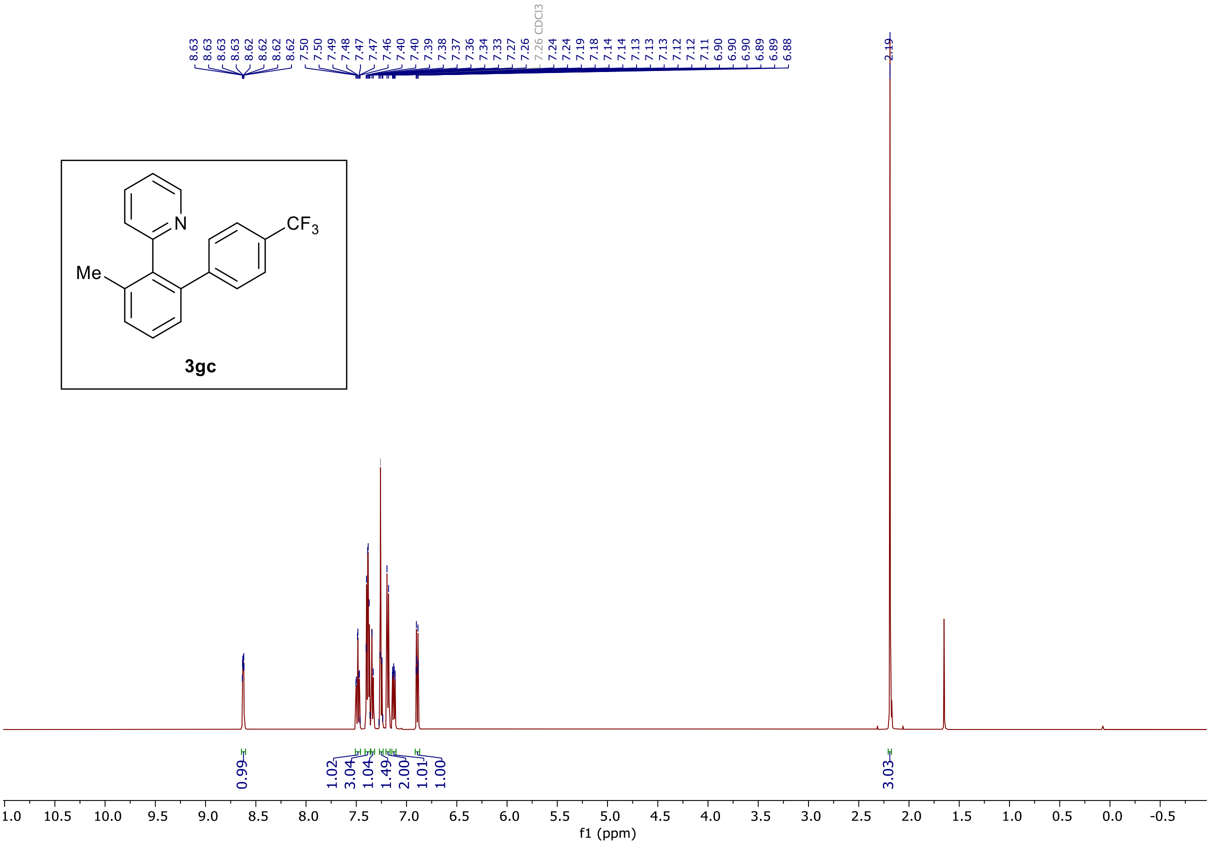

Figure S35. ^{13}C NMR ($CDCl_3$, 126 MHz) of **3gc**.

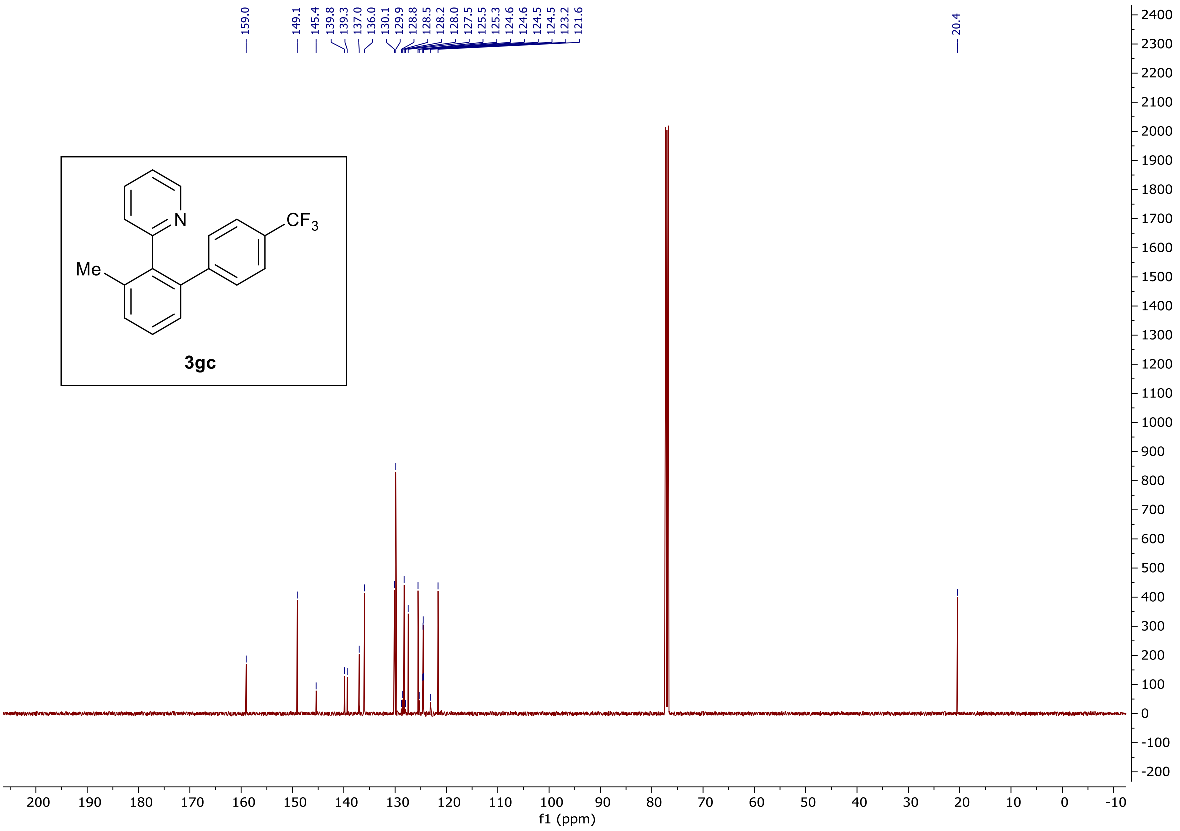

Figure S36. ^{19}F NMR ($CDCl_3$, 471 MHz) of **3gc**.

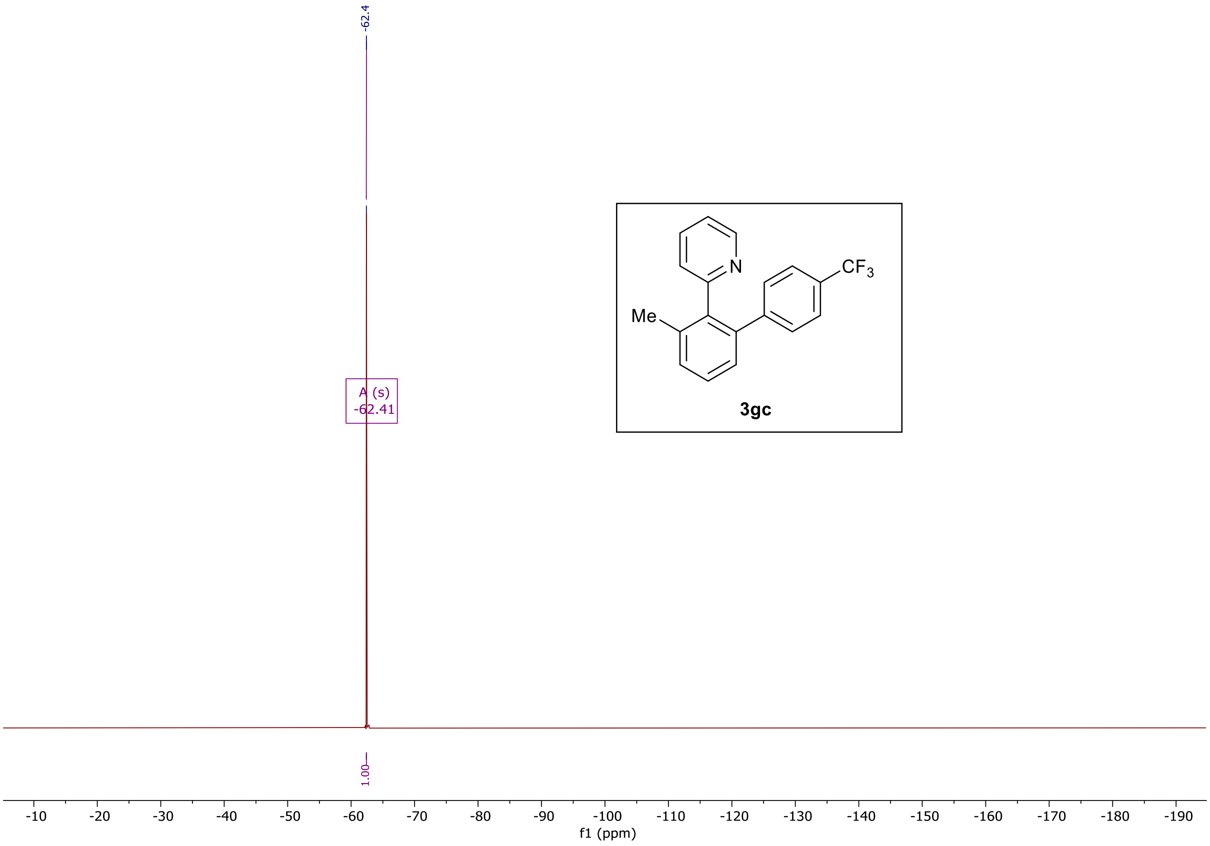

Figure S37. ^{1}H NMR ($CDCl_3$, 400 MHz) of **3gd**.

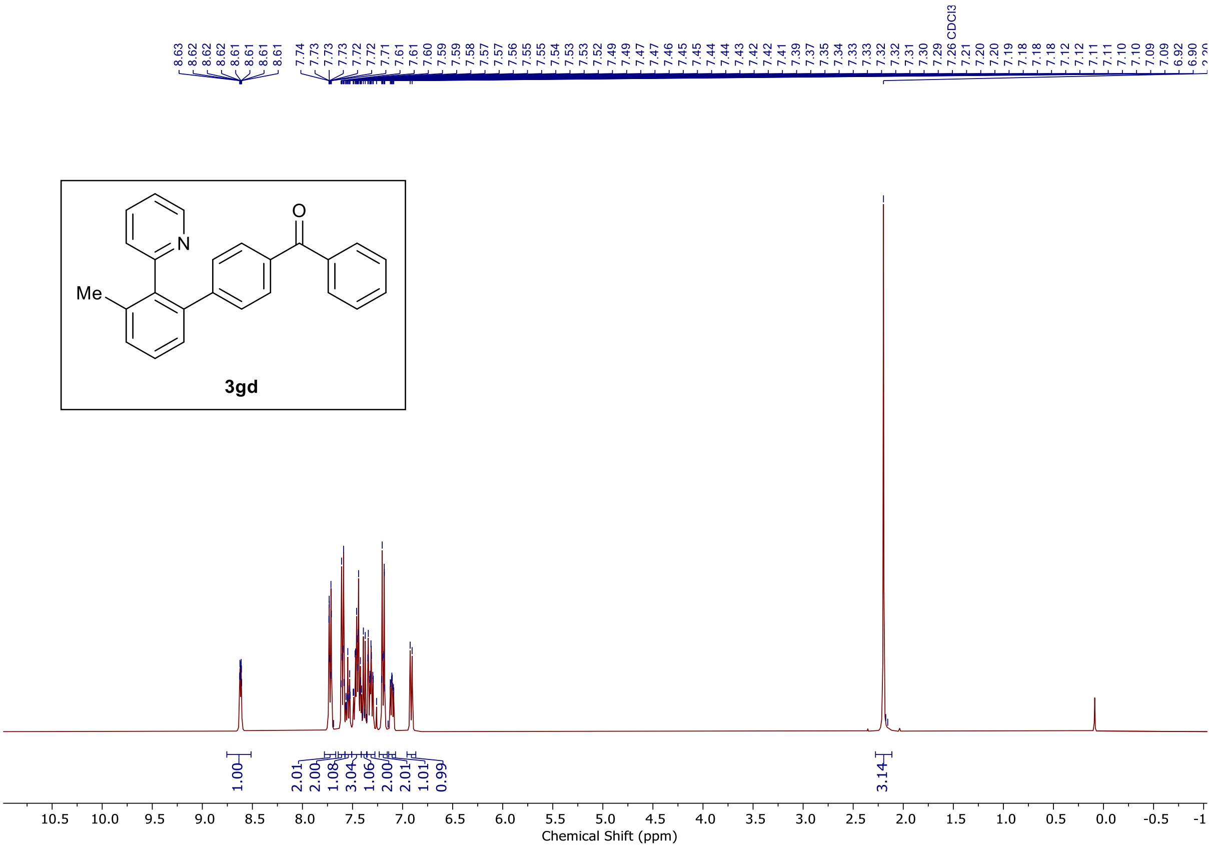

Figure S38. ^{13}C NMR ($CDCl_3$, 101 MHz) of **3gd**.

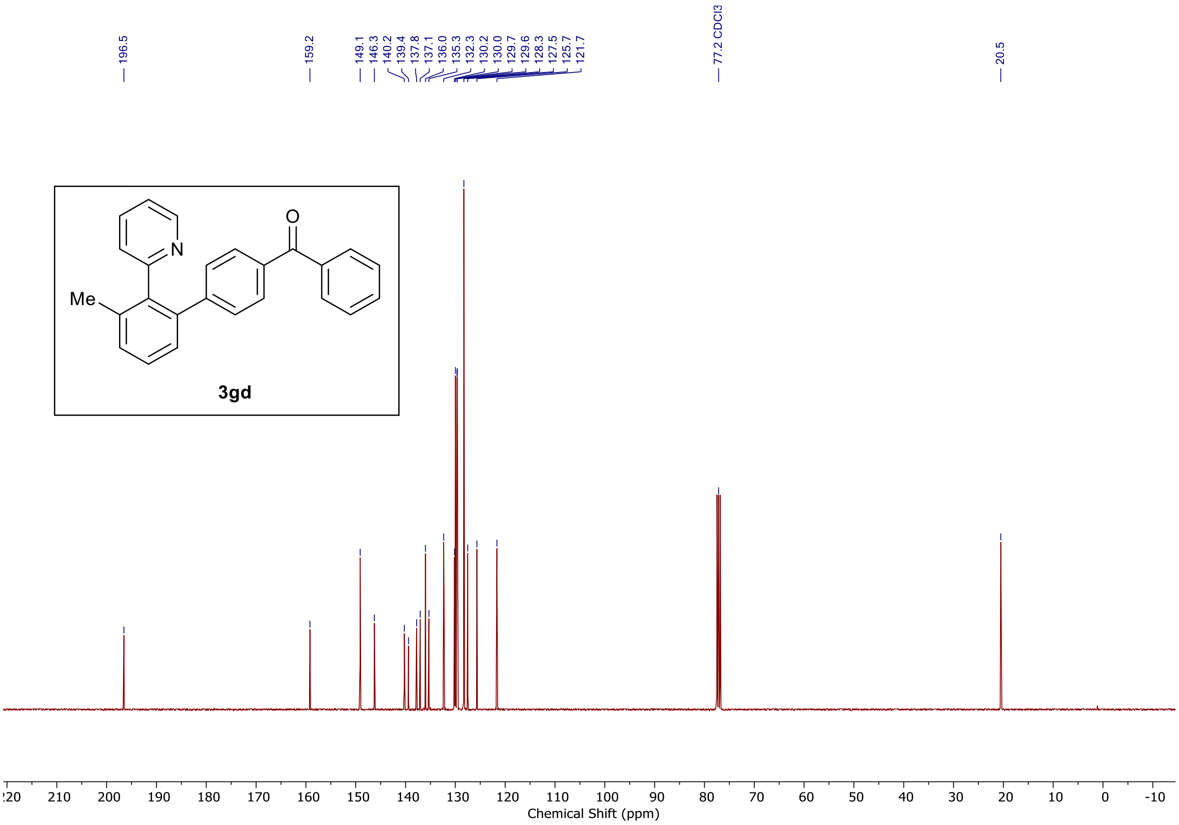

Figure S39. ^{1}H NMR ($CDCl_3$, 400 MHz) of **3ge**.

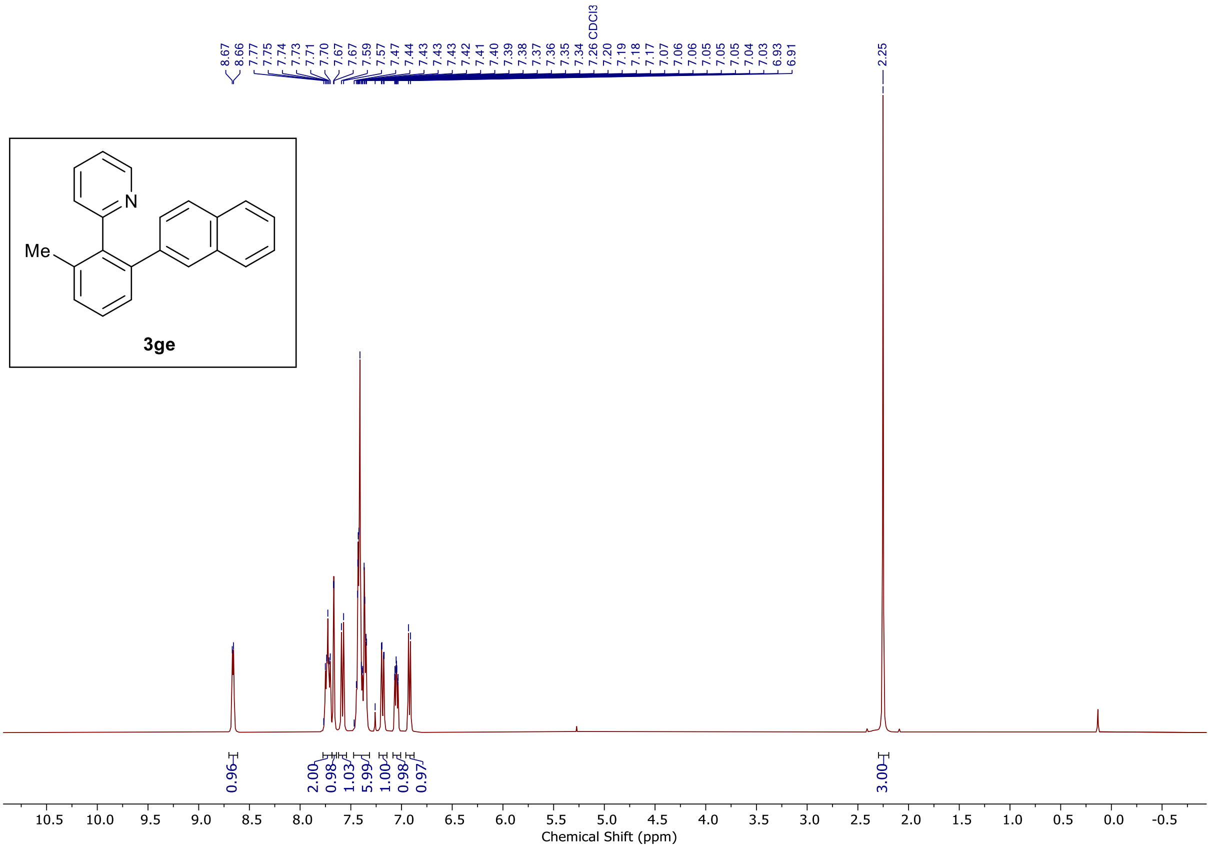

Figure S40. ^{13}C NMR ($CDCl_3$, 101 MHz) of **3ge**.

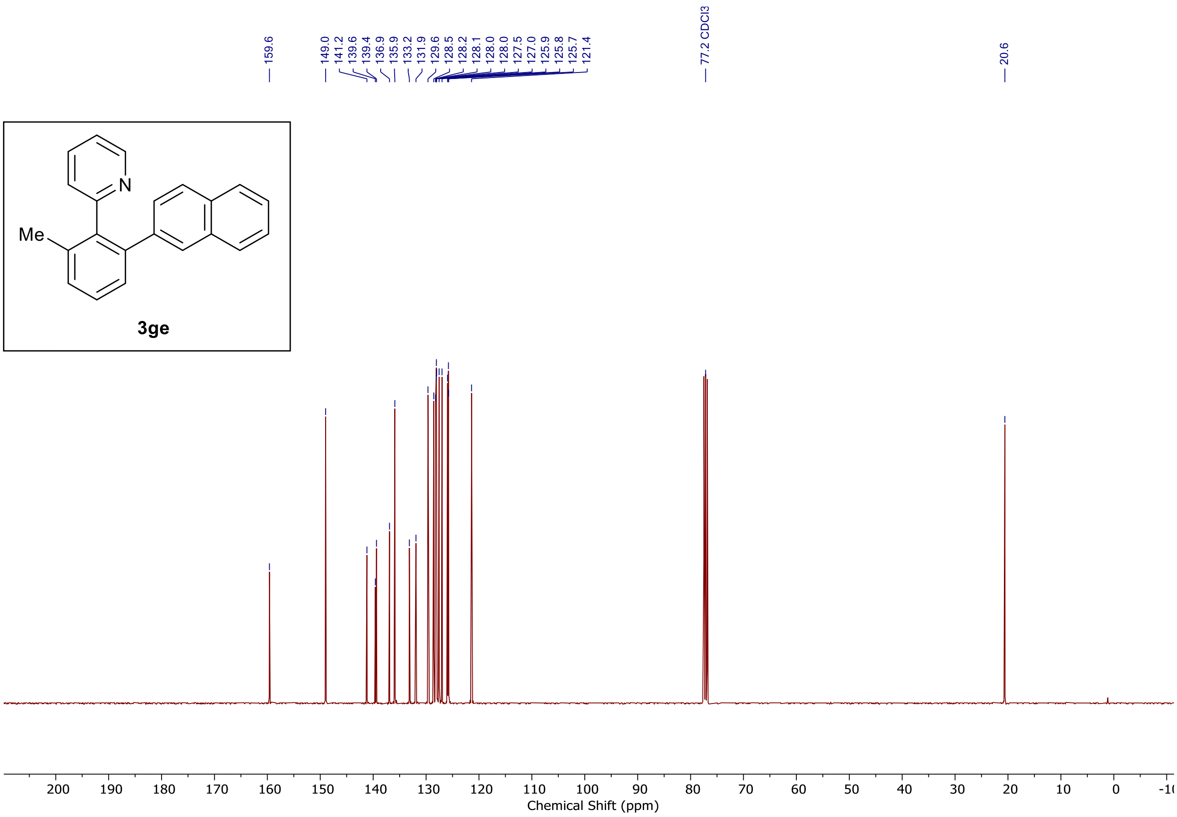

Figure S41. ^{1}H NMR ($CDCl_3$, 400 MHz) of **3gf**.

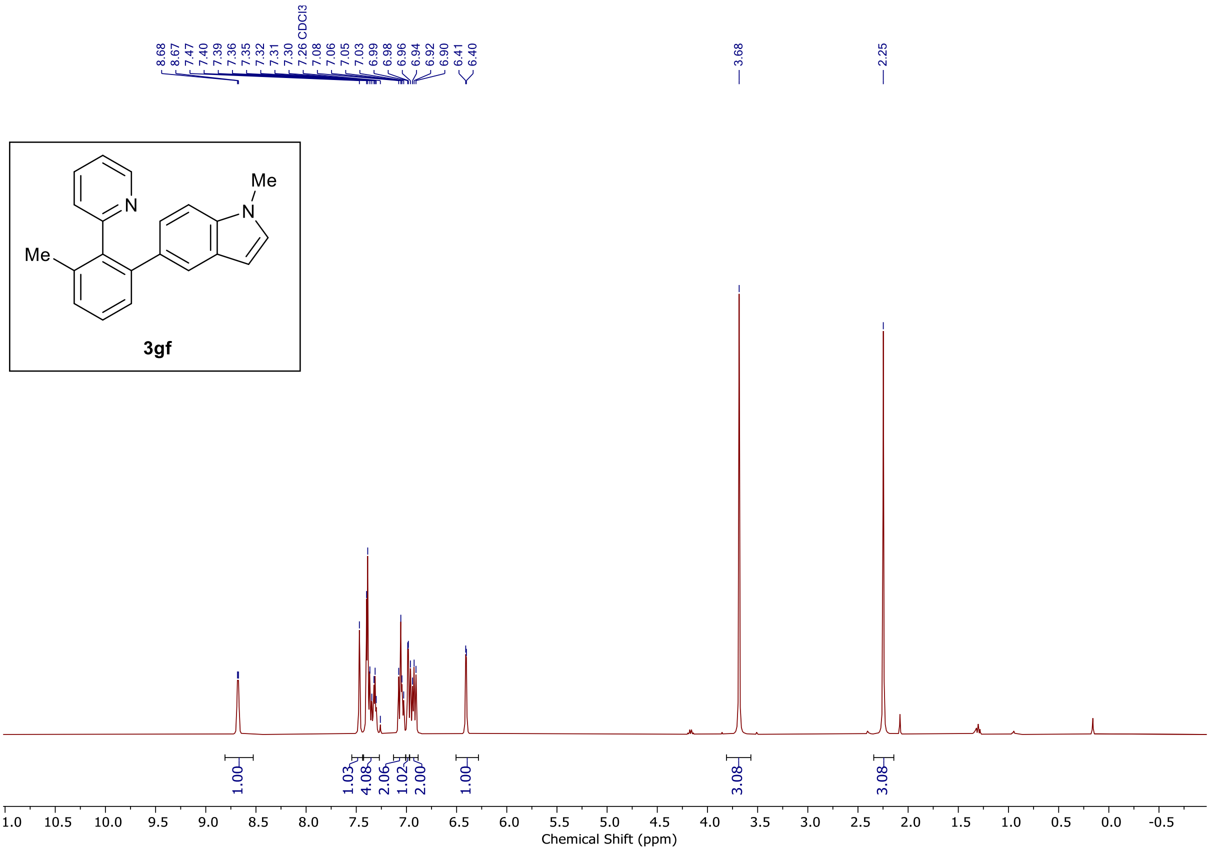

Figure S42. ^{13}C NMR ($CDCl_3$, 101 MHz) of **3gf**.

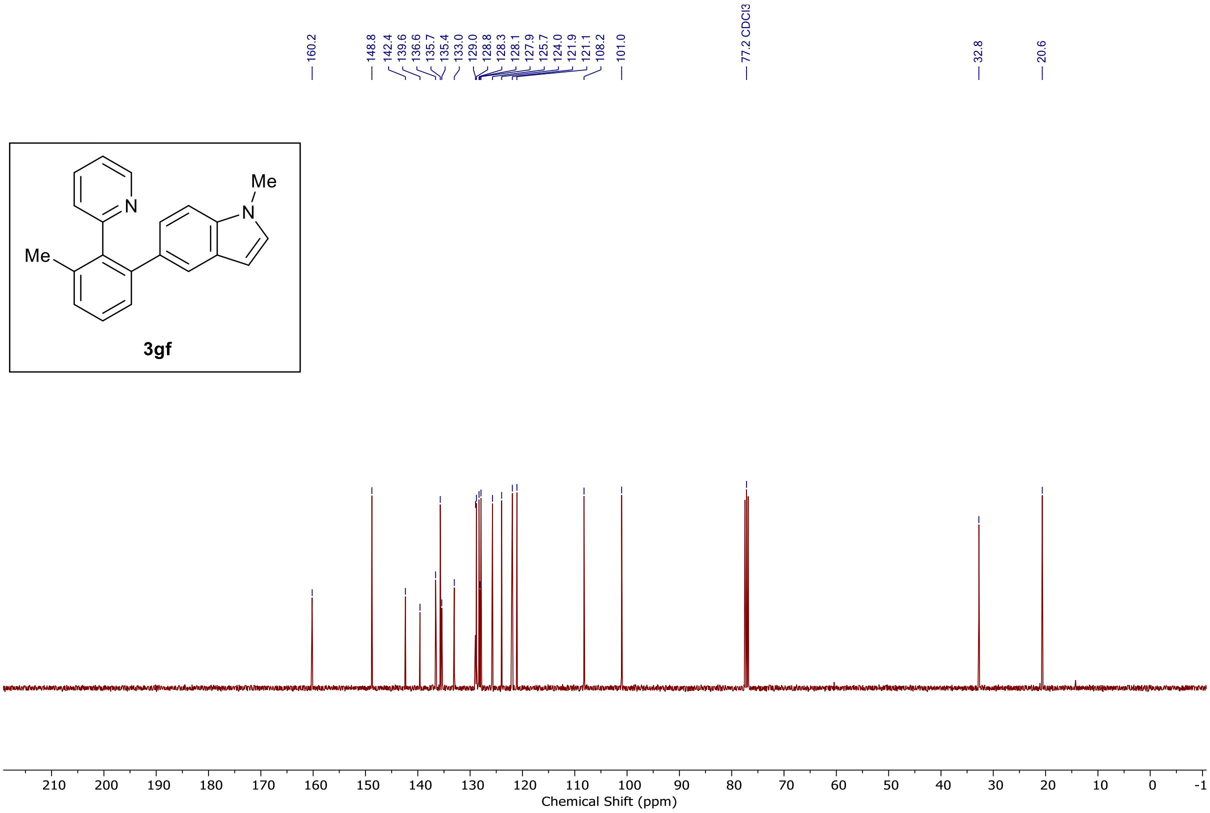

Figure S43. ^{1}H NMR ($CDCl_3$, 400 MHz) of **3gg**.

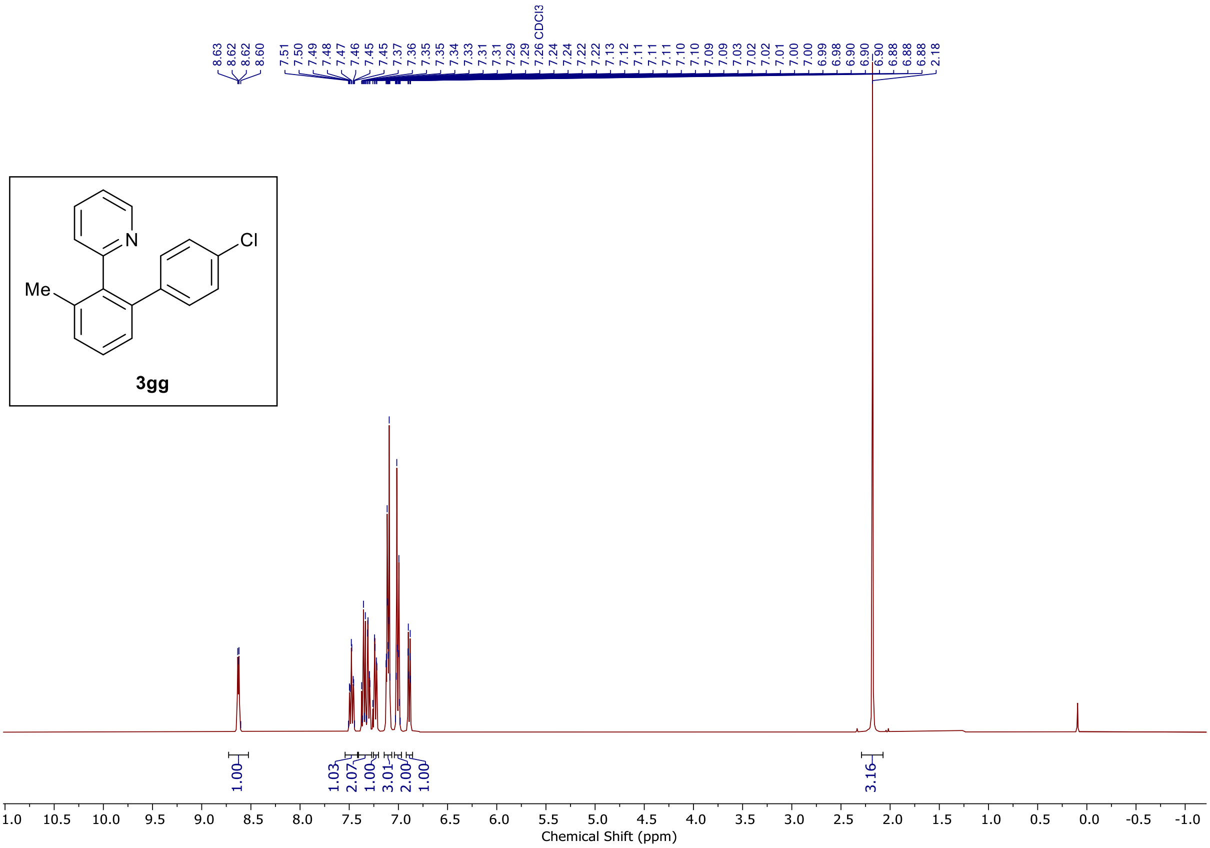

Figure S44. ^{13}C NMR ($CDCl_3$, 101 MHz) of **3gg**.

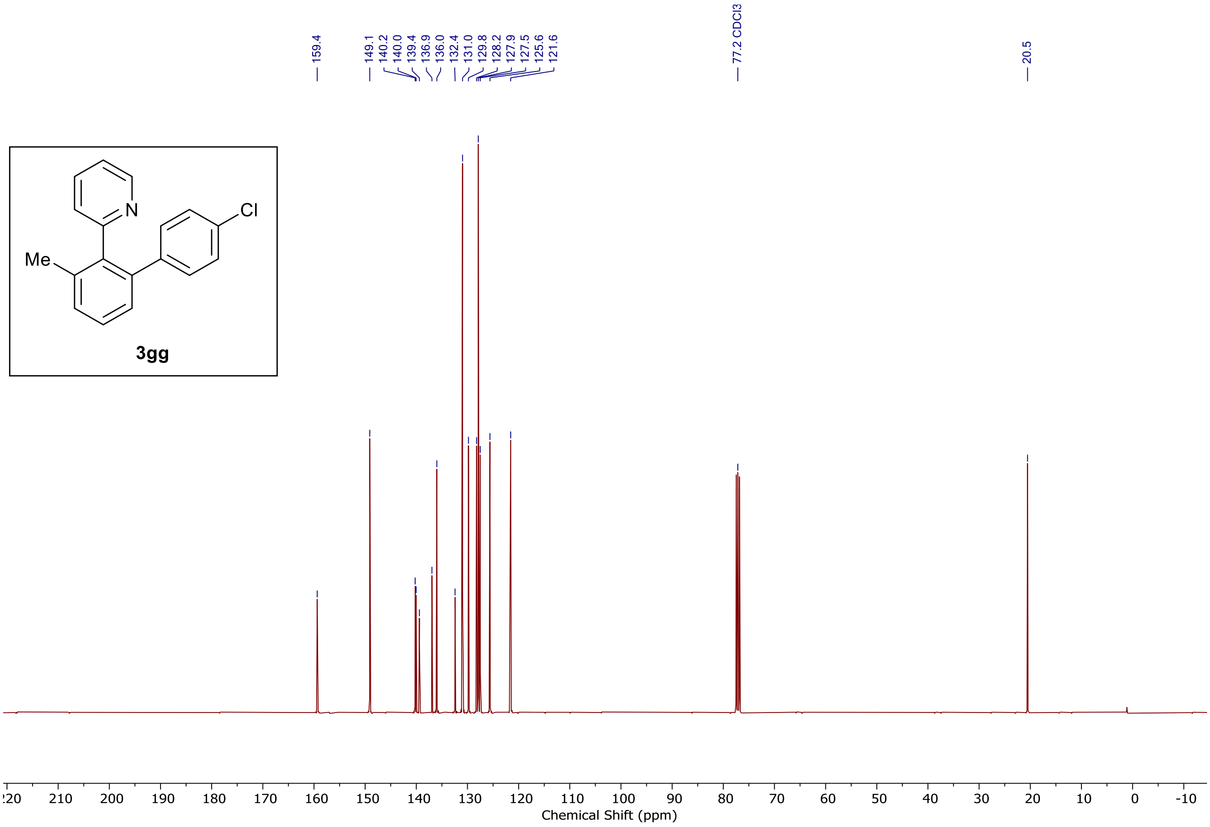

Figure S45. ^{1}H NMR ($CDCl_3$, 500 MHz) of **3gh**.

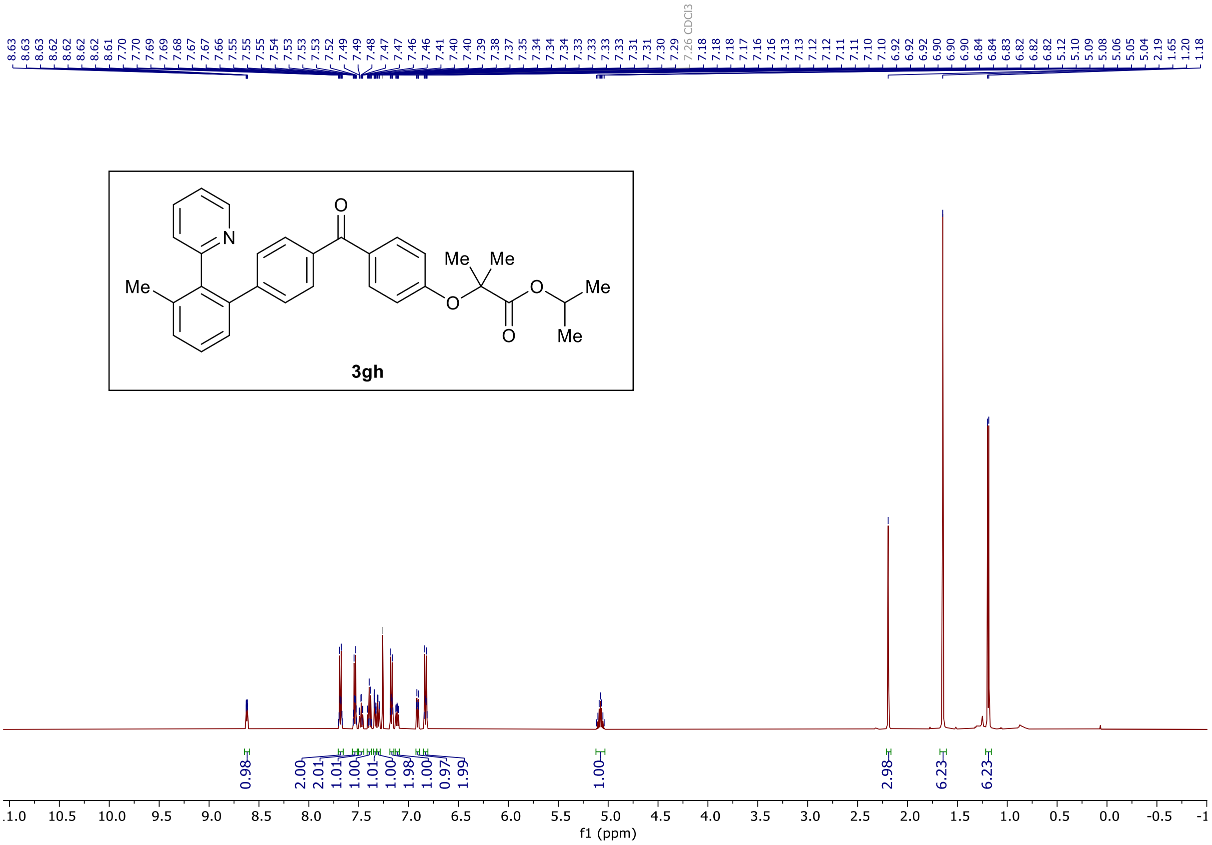

Figure S46. ^{13}C NMR ($CDCl_3$, 126 MHz) of **3gh**.

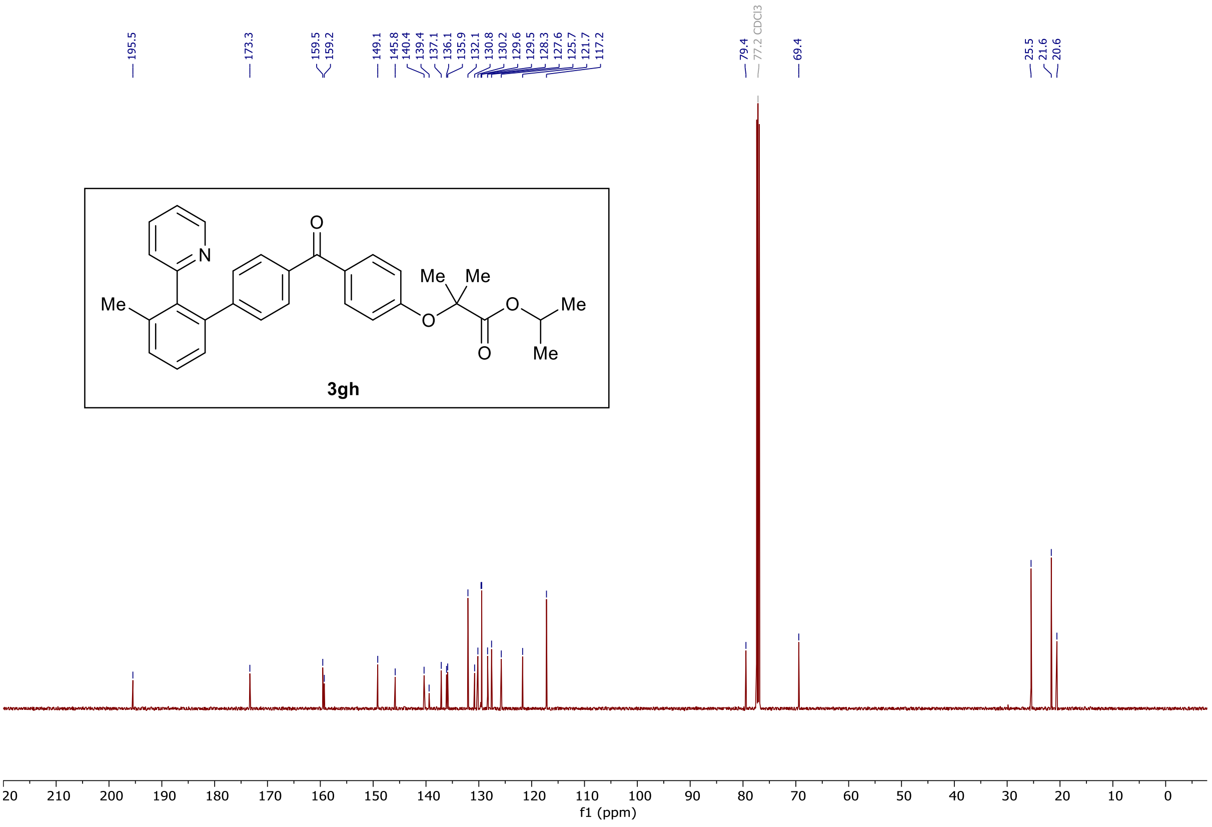

Figure S47. ^{1}H NMR ($CDCl_3$, 400 MHz) of **3gi**.

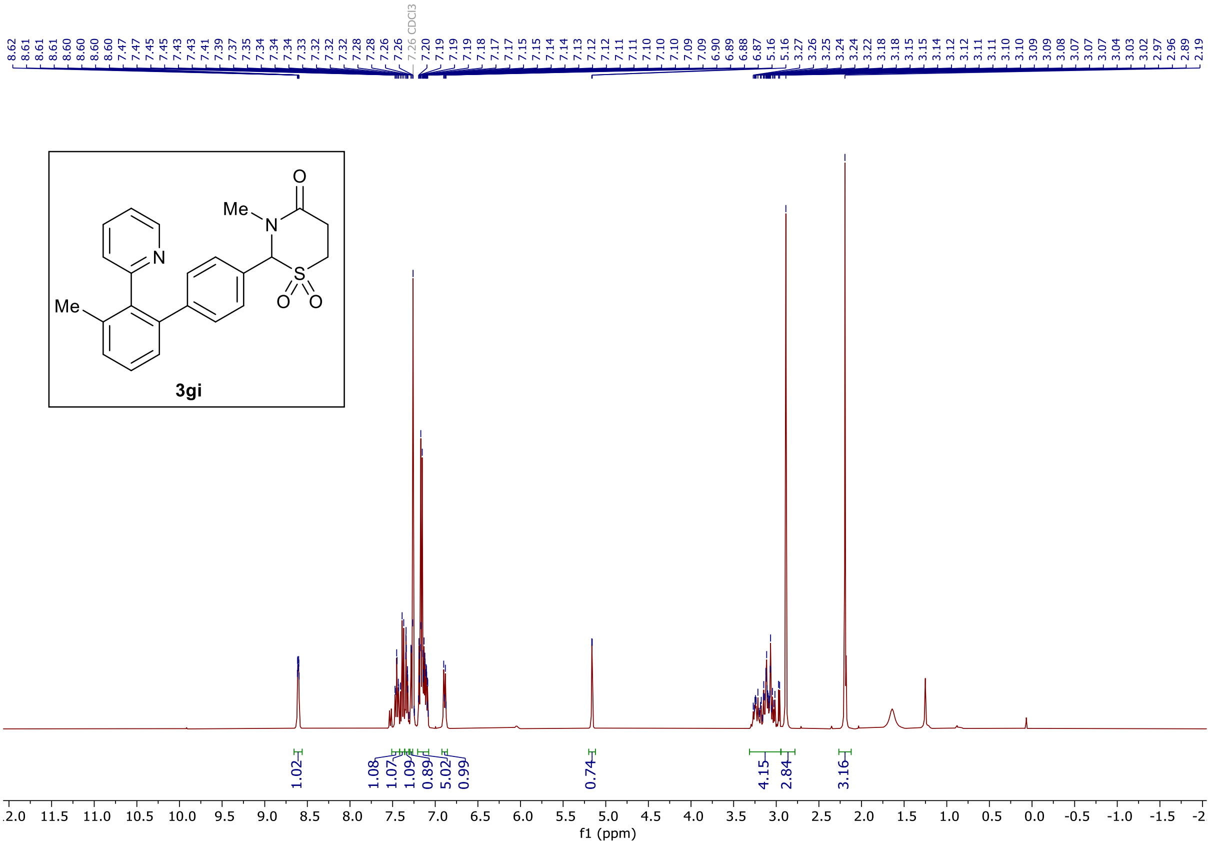

Figure S48. ^{13}C NMR ($CDCl_3$, 101 MHz) of **3gi**.

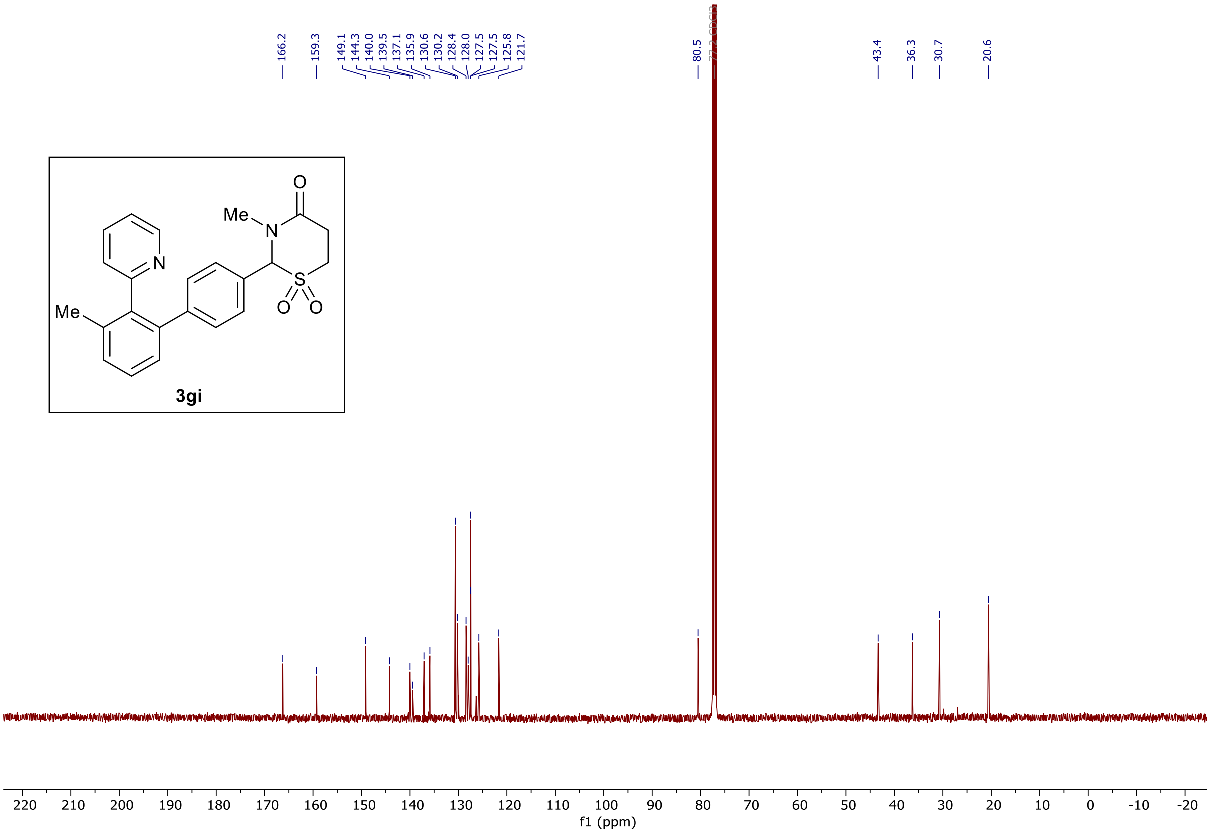

Figure S49. ^{1}H NMR ($CDCl_3$, 400 MHz) of **3gj**.

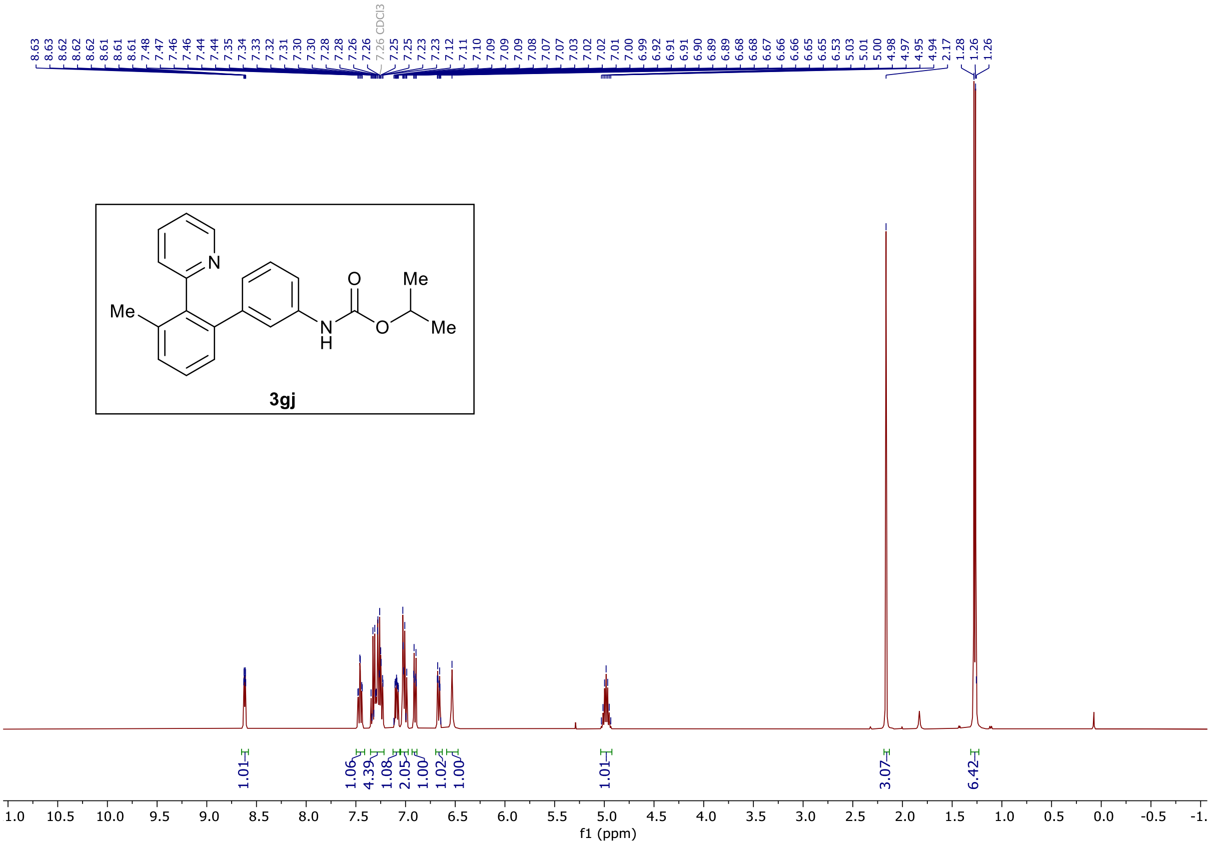

Figure S50. ^{13}C NMR ($CDCl_3$, 126 MHz) of **3gj**.

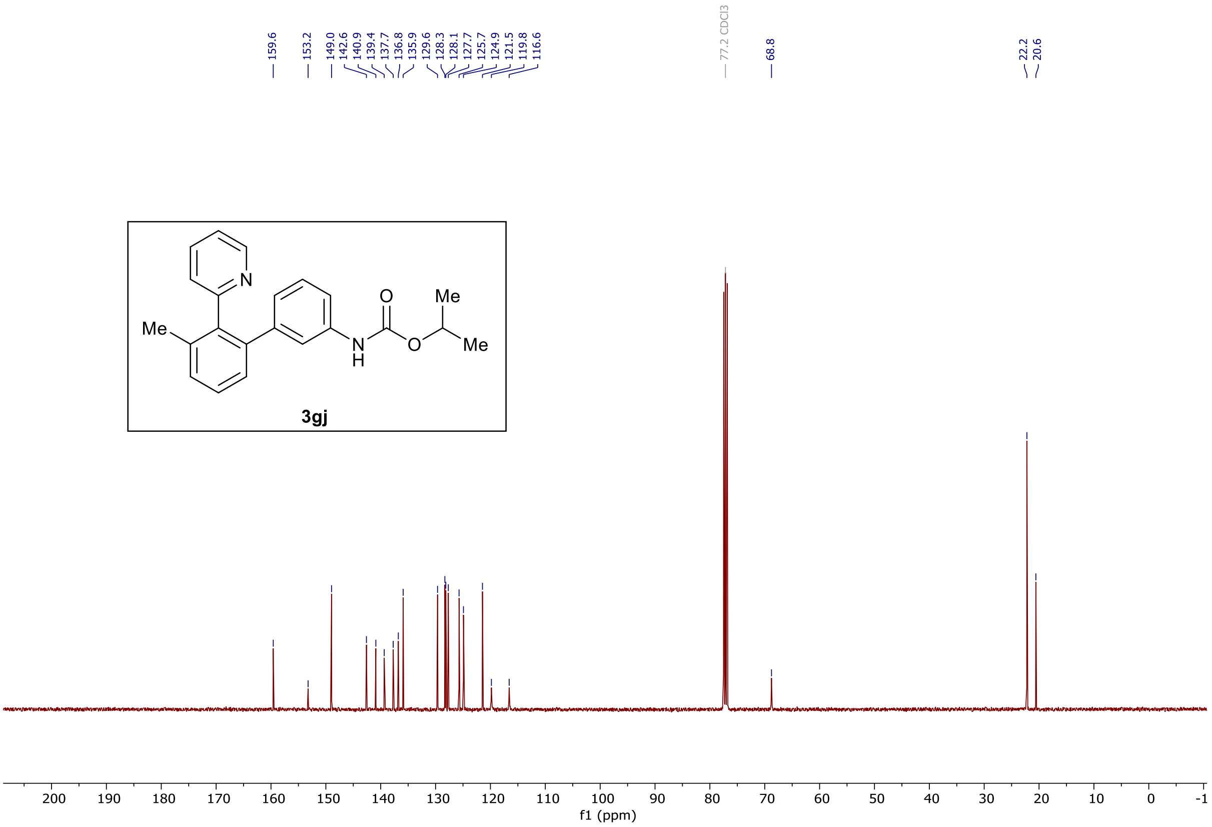

Figure S51. ^{1}H NMR ($CDCl_3$, 400 MHz) of **4ha**.

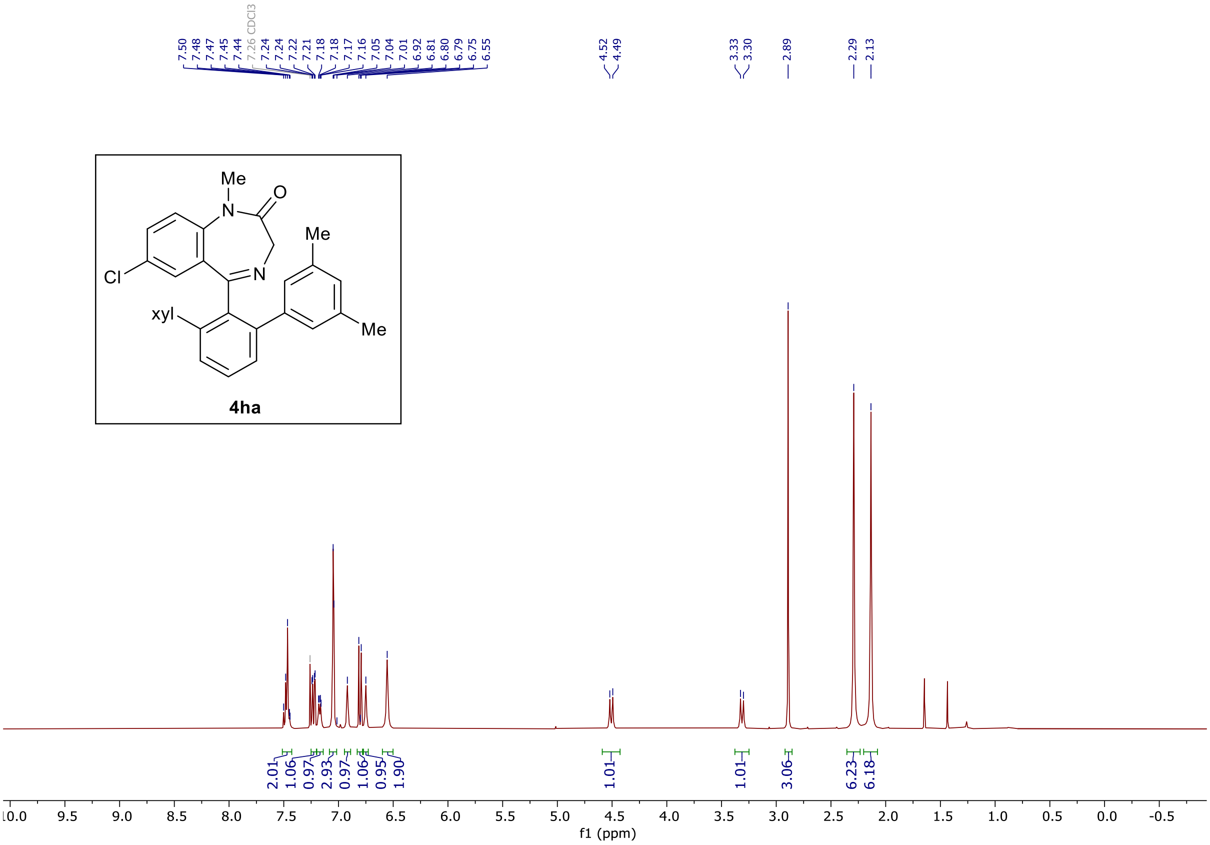

Figure S52. ^{13}C NMR ($CDCl_3$, 101 MHz) of **4ha**.

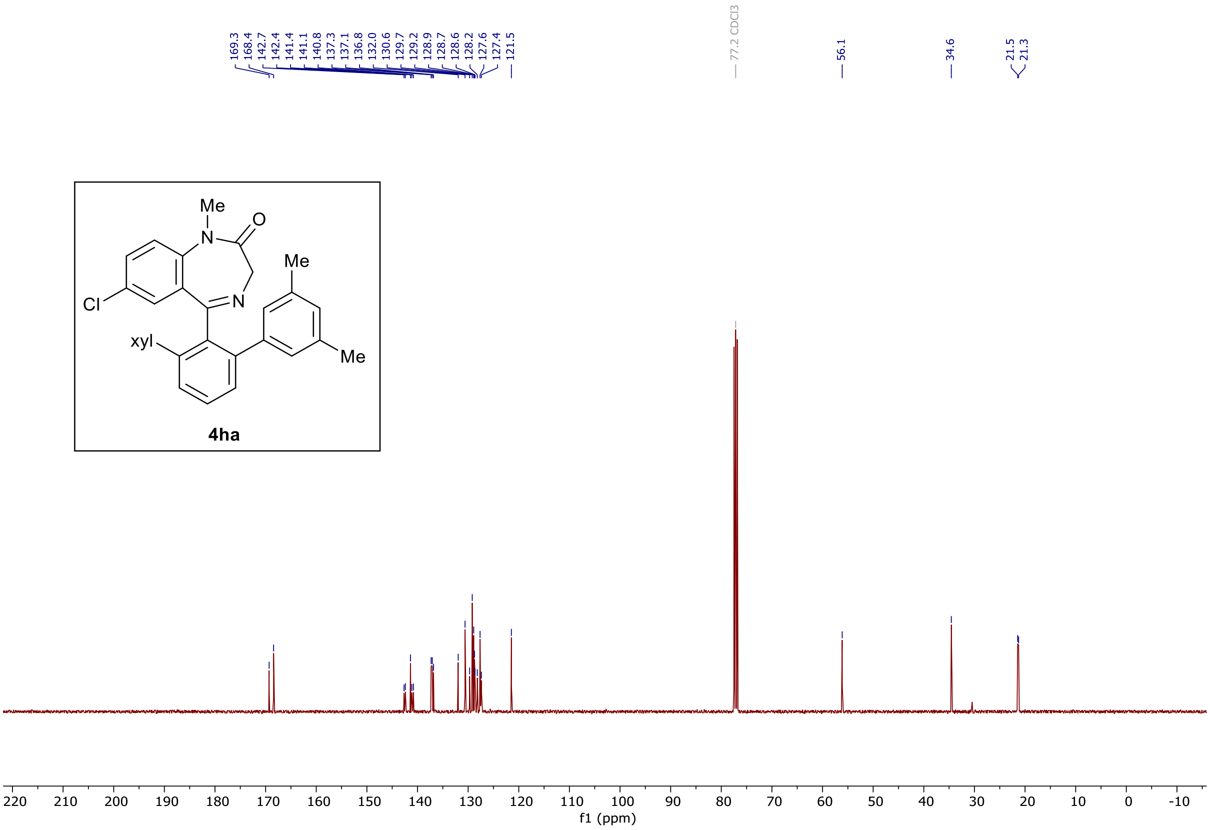

Figure S53. ^{1}H NMR ($CDCl_3$, 400 MHz) of **3ia**.

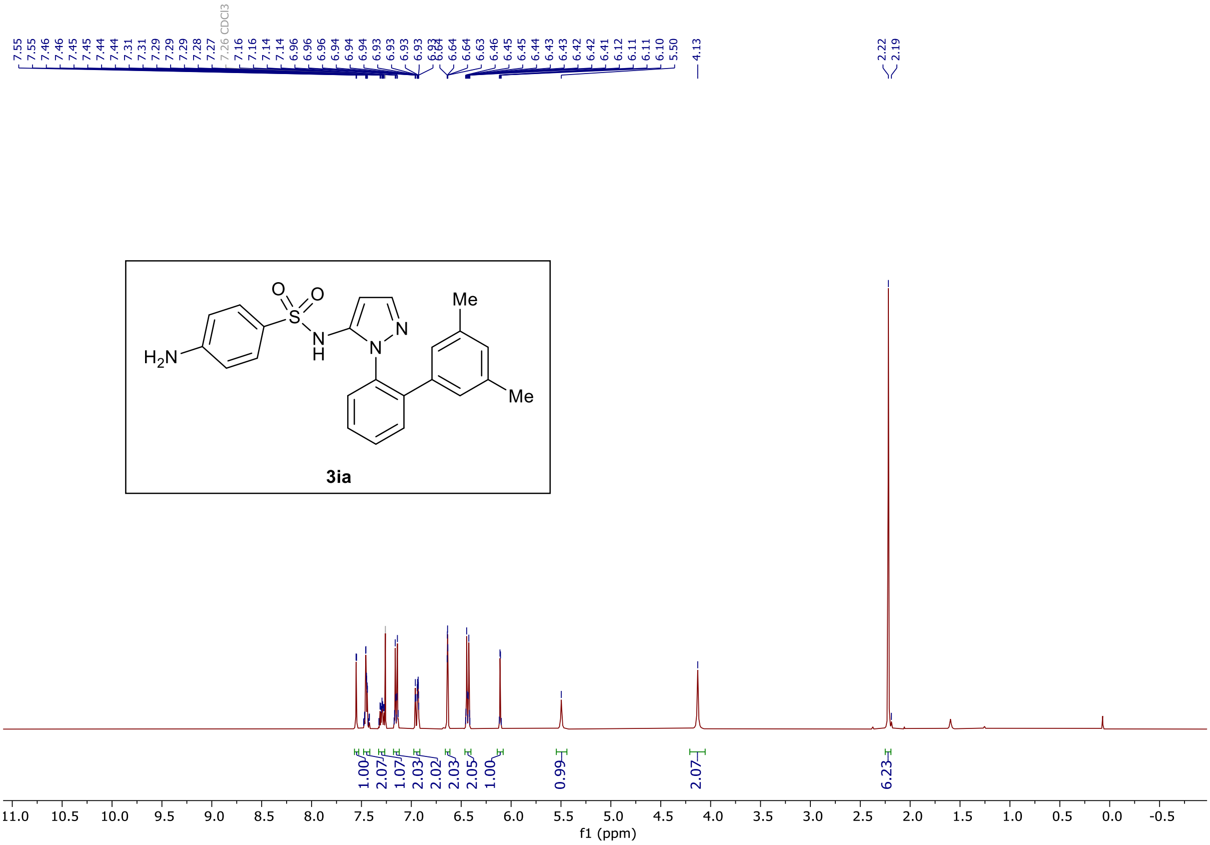

Figure S54. ^{13}C NMR ($CDCl_3$, 101 MHz) of **3ia**.

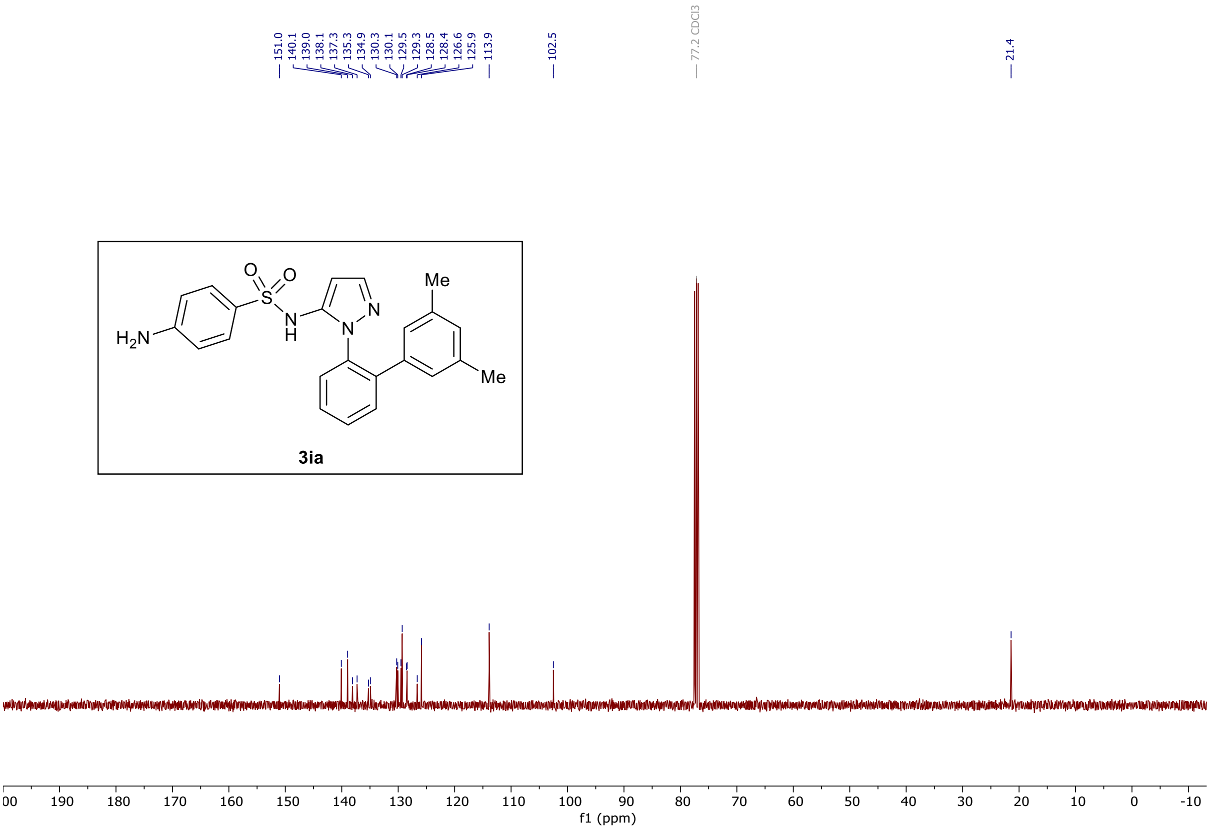

Figure S55. ^{1}H NMR ($CDCl_3$, 400 MHz) of **4ia**.

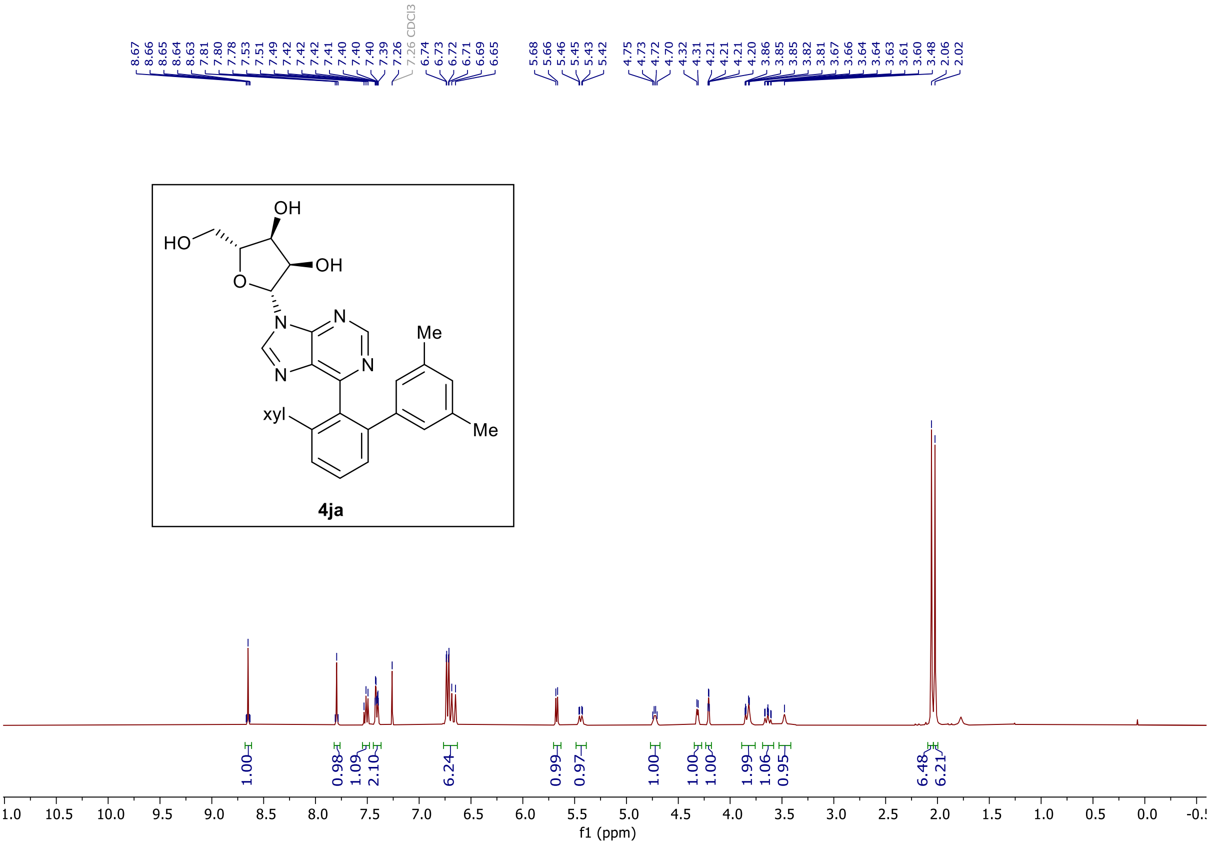

Figure S56. ^{13}C NMR ($CDCl_3$, 101 MHz) of **4ia**.

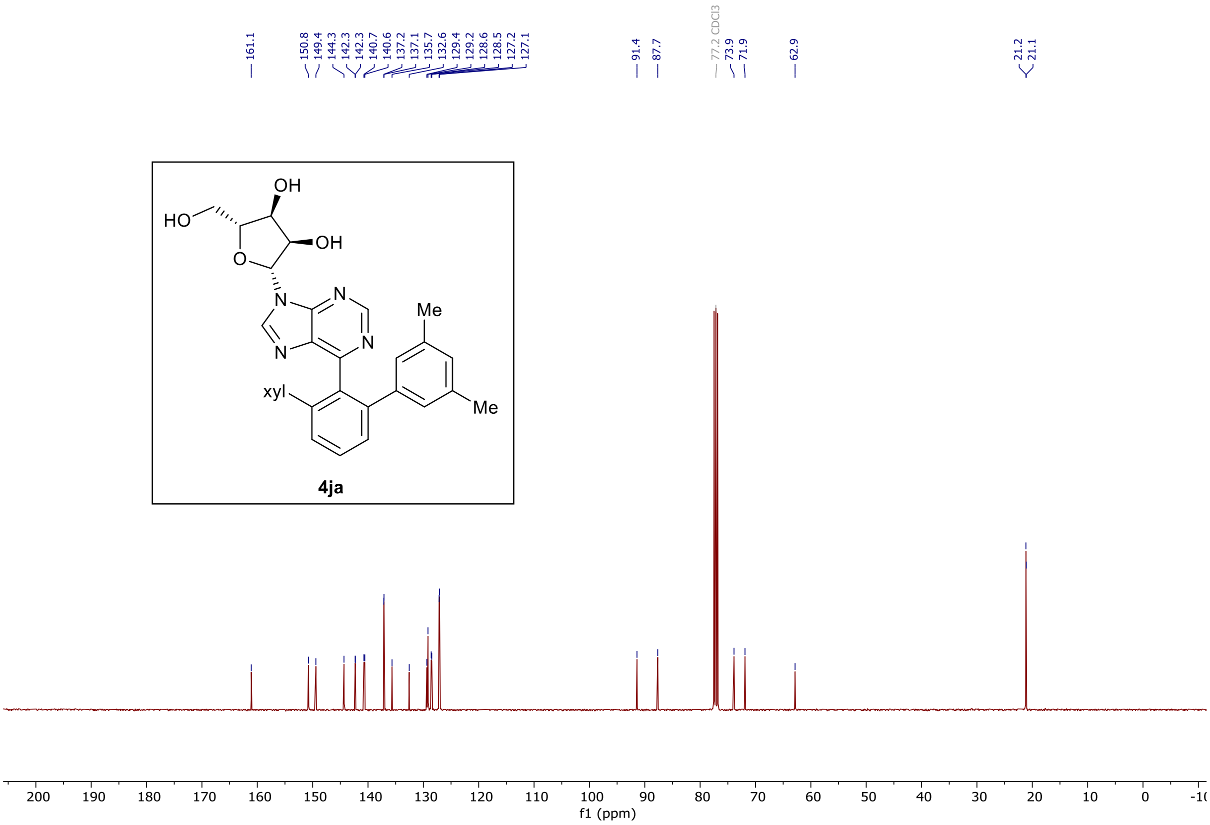

11. References

1. M. Trose, F. Lazreg, M. Lesieur and C. S. J. Cazin, *J. Org. Chem.,* 2015, **80**, 9910−9914.
2. A. J. Paterson, C. J. Heron, C. L. McMullin, M. F. Mahon, N. J. Press and C. G. Frost, *Org. Biomol. Chem.*, 2017, **15**, 5993–6000.
3. J. Park and S. Chang, *Angew. Chem., Int. Ed.*, 2015, **54**, 14103–14107.
4. E. Odella, S. J. Mora, B. L. Wadsworth, M. T. Huynh, J. J. Goings, P. A. Liddell, T. L. Groy, M. Gervaldo, L. E. Sereno, D. Gust, T. A. Moore, G. F. Moore, S. Hammes-Schiffer and Ana L. Moore, *J. Am. Chem. Soc.*, 2018, **140**, 15450–15460.
5. C. Amiable, J. Paoletti, A. Haouz, A. Padilla, G. Labesse, P.–A. Kaminski and S. Pochet, *Eur. J. Med. Chem.*, 2014, **85**, 418–437.
6. M. Simonetti, D. M. Cannas, X. Just-Baringo, I. J. Vitorica-Yrezabal and I. Larrosa, *Nat. Chem.*, 2018, **10**, 724–731.
7. Z. Rong, M. Luo and X. Zeng, *Org. Lett.*, 2019, **21**, 6869–6873.
8. L. Jian, H.–Y. He, J. Huang, Q.–H. Wu, M.–L. Yuan, H.–Y. Fu, X.–L. Zheng, H. Chen and R.–X. Li, *RSC Adv*., 2017, **7**, 23515–23522.
9. L. Ackermann, R. Vicente and A. Althammer, *Org. Lett.*, 2008, **10**, 2299–2302.
10. J. Kim, K. Shin, S. Jin, D. Kim and S. Chang, *J. Am. Chem. Soc.*, 2019, **141**, 4137−4146.
11. Z. Qi and X. Li, *Angew. Chem, Int. Ed.,* 2013, **52**, 8995–9000.
